# Metabolic Phenotyping Study of Mouse Brain Following Microbiome Disruption by *C. difficile* Colonization

**DOI:** 10.3390/metabo12111039

**Published:** 2022-10-28

**Authors:** Olga Deda, Melina Kachrimanidou, Emily G. Armitage, Thomai Mouskeftara, Neil J. Loftus, Ioannis Zervos, Ioannis Taitzoglou, Helen Gika

**Affiliations:** 1 Laboratory of Forensic Medicine & Toxicology, Department of Medicine, Aristotle University of Thessaloniki, 54124 Thessaloniki, Greece; 2 Biomic AUTh, Center for Interdisciplinary Research and Innovation (CIRI-AUTH), Balkan Center B1.4, 10th km Thessaloniki-Thermi Rd., GR 57001 Thessaloniki, Greece; 3 1st Laboratory of Microbiology, Department of Medicine, Aristotle University of Thessaloniki, 54124 Thessaloniki, Greece; 4 Shimadzu Corporation, Manchester M17 1GP, UK; 5 Laboratory of Animal Physiology, Faculty of Veterinary Medicine, School of Health Sciences, Aristotle University of Thessaloniki, 54124 Thessaloniki, Greece

**Keywords:** *Clostridioides difficile*, mice, antibiotics, FMT, metabolic profiling, metabolomics, LC-MS, GC-MS, brain

## Abstract

*Clostridioides difficile* infection (CDI) is responsible for an increasing number of cases of post-antibiotic diarrhea worldwide, which has high severity and mortality among hospitalized elderly patients. The disruption of gut microbiota due to antibacterial medication facilitates the intestinal colonization of *C. difficile*. In the present study, a murine model was used to investigate the potential effects of antibiotic administration and subsequent colonization by *C. difficile*, as well as the effects of three different 10-day treatments (metronidazole, probiotics, and fecal microbiota transplantation), on the brain metabolome for the first time. Four different metabolomic-based methods (targeted HILIC-MS/MS, untargeted RP-LC-HRMS/MS, targeted GC-MS/MS, and untargeted GC-MS) were applied, resulting in the identification of 217 unique metabolites in the brain extracts, mainly glycerophospholipids, glycerolipids, amino acids, carbohydrates, and fatty acids. Univariate and multivariate statistical analysis revealed that CDI, as well as the subsequent treatments, altered significantly several brain metabolites, probably due to gut dysbiosis, and affected the brain through the gut–brain axis. Notably, none of the therapeutic approaches completely restored the brain metabolic profile to the original, healthy, and non-infected phenotype, even after 10 days of treatment.

## 1. Introduction

Antibiotics are useful for fighting or preventing bacterial infections, but they can also disrupt gut microbiota [1]. This imbalance can reduce resistance and allow the colonization of opportunistic pathogens in intestinal lumen, including *Clostridioides difficile* [2,3], an obligate anaerobic, spore-forming, gram-positive bacillus found in mammals [4]. When colonization occurs, *C. difficile* (CD) causes a wide spectrum of gastrointestinal infections, ranging from asymptomatic or mild cases to severe pseudo-membranous colitis, toxic megacolon, and even death [5]. The growing interest in *C. difficile* infection (CDI) comes from the particularly high number of cases and resulting deaths worldwide. For example, the United States is facing a heavy burden, with the number of cases and deaths per year amounting to 500,000 and 30,000, respectively [1,6].

The main risk factor for developing CDI is the frequent use of antibiotics, such as clindamycin, cephalosporins, penicillin, and, more recently, fluoroquinolones [2], used to treat nosocomial infections (NIs) [7]. Colonization of *C. difficile* disrupts the equilibrium of gut bacteria, and thus, dysbiosis is established. There is increasing evidence that gut dysbiosis is correlated with some of the more common mental health conditions, such as depression, anxiety, obsessive-compulsive disorder (OCD), schizophrenia, bipolar disorder (BP), and dementia [8,9]. CDI has also been linked to mental health conditions [10,11,12], such as Autism Spectrum Disorders (ASD), Parkinson’s disease, Alzheimer’s disease, and Multiple sclerosis. The mechanism behind this correlation is the gut–brain axis, in which the gut microbiome either communicates with the brain directly through vagal nerves or communicates indirectly through gut symbiome-derived metabolites involved in endocrine signaling and immune system activation, as well as neural and metabolic signaling [9]. This can trigger several psychiatric conditions [13], indicating that brain biochemistry and metabolism can be affected by dysbiotic conditions. Antibiotics, such as metronidazole and vancomycin, are usually used in the first line of therapy for primary CDI [3,4]. Unfortunately, about one-third of patients may experience recurrence since antibiotic treatment alone usually fails to cure the disease triggered by CDI [6].

Probiotics have been extensively studied as prevention agents rather than treatment agents for CDI [14,15]. Very recently, a published study investigating the effect of probiotics alone or in combination with vancomycin and metronidazole on fecal metabolism indicated that high doses of *Bifidobacterium breve* YH68 administrated alone or in combination with these antibiotics offered the most promising results regarding the treatment and final survival rate of CDI in mice [4]. Τhe positive effect of the probiotic intervention was attributed to an increase in the relative abundance of beneficial strains of gut microbiota that antagonize and reduce the numbers of *C. difficile* and therefore affect the fecal metabolome, leading to elevated levels of secondary bile acids and butyric acid, and reduced levels of primary bile acids and indoles [4]. Promising results have been obtained after a single dose of *Lactobacillus reuteri,* which has been administered for both the prevention and treatment of CDI in a murine model [16]. Although scientific data encourage the use of probiotics in order to restore eubiosis, clinicians have been hesitant to adopt probiotic therapies in the clinical practice of CDI management and treatment because of doubts about the duration of the offered protection [17].

Fecal microbiota transplantation (FMT) aims to restore the normal composition of gut microbiota and has recently been recognized as a highly efficacious therapy in the fight against the indefinite cycles of recurrent CDI (R-CDI) syndrome [6,18], but its mechanism of action still requires thorough investigation. However, this powerful therapeutic tool is also accompanied by certain drawbacks related to the use of FMT in clinical practice, including, among others, the invasive administration and the risk of transmission of infection from donor to recipient [19]. Therefore, a deeper understanding of the FMT mechanism and exploiting the knowledge to develop efficient and safe microbiome therapies should be a major clinical priority for the management of CDI.

In the present study, a non-toxigenic bacterial pathogen (*Clostridioides difficile*) was used to further disrupt the vulnerable gut microbiome of previously antibiotic-treated mice with the aim of investigating the effect of gut microbiome disruption on the brain metabolome. As mentioned above, the gut–brain axis involves a bi-directional communication of multiple pathways, including neural, immune-related, endocrine, and metabolic signaling [20]. Thus, dysbiosis caused by antibiotic administration and subsequent colonization of *C. difficile* spores can have an impact on brain metabolism. Brain tissue extracts from infected mice and then treated with metronidazole (G1), probiotics (G2), or fecal microbiota transplantation (FMT) (G3) were studied in comparison to uninfected mice (G5) and to infected but untreated mice (G4) using a metabolomics approach. Metabolic profiling by both untargeted and targeted methods was implemented to explore the effects of bacterial infection and treatment on the gut–brain axis. Probiotic administration was selected as it is a mild means for restoring the gut microbiome, while FMT is an interesting alternative to antibiotic treatment. To our knowledge, this is the first study of the impact of gut microbiome disruption through bacterial infection and treatment with antibiotics, probiotics, or FMT on the brain metabolome.

## 2. Materials and Methods

### 2.1. Reagents and Materials

Methanol (MeOH), Methoxyamine hydrochloride (MeOX), *N*-Methyl-*N*-(trimethylsilyl) trifluoroacetamide (MSTFA), trimethylchlorosilane (TMCS), and pyridine anhydrous were purchased from Sigma-Aldrich (Merck, Darmstadt, Germany). Internal standards 4-phenylbutyric, myristic acid-d27, and injection standard *N*-pentadecane were also obtained from Sigma-Aldrich (Merck, Darmstadt, Germany). Acetonitrile, Methyl-tert-butyl-ester (MTBE), and ammonium formate were obtained from CHEM-LAB NV (Zedelgem, Belgium). Deionized water was obtained from a Milli-Q ultra-pure-grade water system (Millipore, Bedford, MA, USA). LC-MS grade acetonitrile and water used in the RP-HRMS/MS analysis were purchased from Romil Ltd., Cambridge, UK. Metabolite standards and formic acid used in the RP-LC-HRMS/MS analysis were purchased from Sigma-Aldrich (Gillingham, UK). Antibiotics, including metronidazole, vancomycin, kanamycin, gentamycin, colistin, and clindamycin, were purchased from Sigma–Aldrich (St. Louis, MO, USA).

### 2.2. C. difficile Spore Purification

A non-toxigenic (*tcdA^-^ tcdB^-^ cdtA^-^ cdtB^-^) C. difficile* strain [21] was cultured onto a Columbia blood agar plate and incubated anaerobically at 37 °C for 48 h [22]. The following day, the inoculum was added to 40 mL autoclaved Clospore liquid medium culture prepared as described by Perez et al., 2011 [23]. The 16-day incubation took part at 37 °C under anaerobic conditions [24]. Harvesting and purification of spores was performed by 3 cycles of 20 min centrifugation at 10,000× *g*. Subsequently, the formed pellet was washed at least 3 times with sterile deionized water and stored at 4 °C for a short period. At the beginning of the in vivo experiment, *C. difficile* spores were heated in a water bath at 65 °C for 20 min to kill vegetative cells. To achieve the desired density (2000 spores/20 μL), spores were further diluted in sterile water. Before dilution, the spores were counted microscopically after Schaeffer–Fulton endospore staining [25]. Viable spores were also enumerated for cross-checking of the desired dose by plating colony-forming units (c.f.u.) mL^−1^ on taurocholate, cefoxitin, cycloserine, and fructose agar (TCCFA) after thermal shocking [22] and anaerobic incubation for 48 h at 37 °C. Verification of *C. difficile* colonization was performed by culturing homogenized mouse fecal samples on TCCFA plates. After homogenization with sterile PBS and vigorous vortexing, fecal samples were plated on TCCFA plates after 1h treatment with 50% ethanol. All procedures were performed under biosafety level II conditions.

### 2.3. Animal Experiment

The animal study took place in the Laboratory of Development–Breeding of Animal Models and Biomedical Research, School of Health Science, Aristotle University of Thessaloniki, Greece. All procedures were conducted in accordance with the 2010/63/EU Directive and Presidential Decree No. 56/2013 of Greek legislation for the care and use of laboratory animals and were approved by the Department of Rural Economy and Veterinary Medicine, Prefecture of Central Macedonia, Hellenic Republic [Protocol number: 634754(2485)].

Fifty C57BL/6 male mice were bred under a regulated 12 h light/12 h dark cycle and controlled temperature (22–25 °C) and humidity (50%) conditions, receiving autoclaved food, water, and bedding. At their twelfth week of age, the animals were divided into five groups housed in group cages of five animals each (*n* = 5) following an acclimatization period of one week before the initiation of the treatment. Then, for four days, mice of groups G1 to G4 were exposed to an antibiotic cocktail containing metronidazole 0.215 mg mL^−1^, vancomycin 0.045 mg mL^−1^, kanamycin 0.4 mg mL^−1^, gentamycin 0.035 mg mL^−1^ and colistin 850 U mL^−1^ via their drinking water.

On the sixth day, after one day of no intervention, a single intraperitoneal injection of clindamycin 10 mg kg^−1^ was administrated [26,27]. The next day, the four animal groups (G1, G2, G3, and G4) were challenged with the non-toxigenic (TCDA^-^, TCDB^-^) bacterial pathogen (*Clostridioides difficile*) through their drinking water, which was prepared by suspending *C. difficile* spores (10^4^ spores mL^−1^) for 24 hours [24]. The animals in the fifth group (G5, *n* = 10) were not treated with antibiotics and did not come in contact with the pathogen (control group). Verification of *C. difficile* colonization in the mice of G1–G4 was performed by culturing homogenized mouse fecal samples, as described in Section 2.2.

Three days after infection with *C. difficile*, animals in groups G1, G2, and G3 underwent different treatments, each for a period of 10 days via their daily drinking water, while the fourth infected group (G4, *n* = 10) remained untreated. The treated animals received either metronidazole (50 mg/kg/day) (G1, *n* = 10) [28], or a commercially available probiotic product containing 5 × 10^9^ viable strains: *L. casei, B. lactis, B. longum, B. bifidum, L. salivarius, L. bulgaricus, L. plantarum, L. rhamnosus, L. acidophilus, S. thermophiles, Lactococcus lactis* (G2, *n* = 10) or were subjected to Fecal Microbiota Transplantation (FMT) by receiving 10% fecal water prepared from the fecal samples of controls (400 mg/mL) diluted in sterile PBS [29] (G3, *n* = 10). Τhe water consumption was measured and recorded daily for every group of mice, and no statistically significant differences were observed.

**Figure 1:**
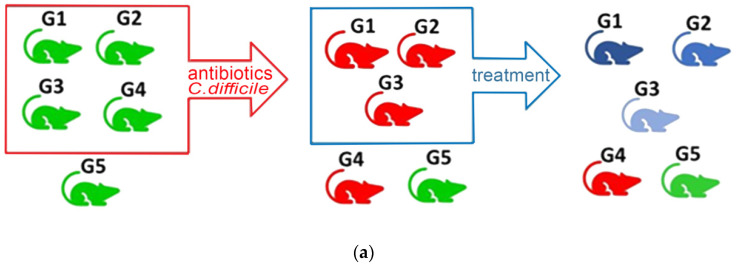

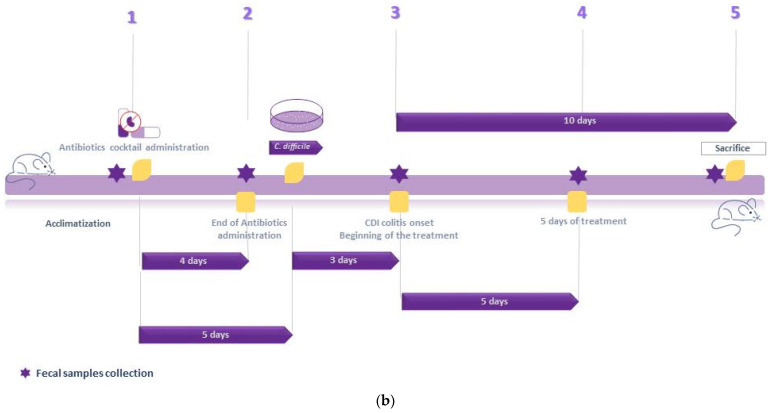
Graphical illustration of (**a**) the studied animals and (**b**) the timeline of the experimental design. From five groups of mice, four (G1, G2, G3, G4) were treated with a cocktail of antibiotics and then challenged with *C. difficile*. Then, three of them (G1, G2, G3) were subjected to therapeutic treatments MTR, probiotic, and FMT (G1, G2, and G3, respectively).

The health status of the mice was recorded daily throughout the experiment, and their body weights were recorded weekly. Fecal samples were collected at all critical time points, while tissues were collected postmortem after each mouse was sacrificed by cervical dislocation. The collected brain tissues were washed with saline solution, frozen in liquid nitrogen, and stored at −80 °C. All procedures (bedding changes, infection, and sample collection) were performed under a laminar flow hood and using appropriate personal protective equipment by trained lab animal science technicians and veterinarians. The laminar flow hood work area was sterilized between experimental treatments to prevent potential cross-contamination by spores. A schematic illustration of the experimental design, including the respective timelines, is given in Figure 1.

### 2.4. Metabolites’ Extraction

Brain tissues were left to thaw at room temperature and then homogenized using a Bead mill Homogenizer (BEAD RUPTOR ELITE, Omni International, Kennesaw, Georgia). Two extracts were obtained for hydrophilic and lipophilic metabolites using a two-step procedure.

First, tissues were weighed (360.82 mg ± 40.22) and transferred to 1.5-mL tubes containing 1.0 mm zirconium oxide beads. A solvent mixture of MeOH—IPA—H_2_O, 1:1:2 (*v*/*v*/*v*) in a ratio of tissue weight/solvent volume of 1:3 (*w*_br_/*v*_sol_) was added, and then the samples were vortexed, sonicated, and homogenized (3 cycles of 30 s, at a speed of 6.00 m/s). The homogenates were centrifuged for 20 min at 10,000× *g* and the supernatant hydrophilic extracts were collected and divided into 4 aliquots for different analyses; 200 μL were retrieved for HILIC-MS/MS analysis and 100 μL for GC-MS analysis. All aliquots were evaporated to dryness (SpeedVac, Eppendorf Austria GmbH, Wien, Austria).

In the second step, the pellet residues remaining in the 1.5 mL tubes containing zirconium oxide beads were further extracted for lipophilic metabolites. A solvent mixture of MTBE—MeOH 3:1 (*v*/*v*) was added to the dry pellet in a proportion of tissue weight/solvent volume 1:3 (*w*_br_/*v*_sol_) and then, the samples were vortexed for 20 min. After centrifugation for 30 min at 10,000× *g*, 600 μL of the supernatant extracts were transferred to 1.5 mL Eppendorf tubes and evaporated to dryness by Speedvac for RP-LC-HRMS/MS analysis.

The same extraction procedure was performed for the preparation of procedural blank samples, and finally, all evaporated sample extracts were kept at −80 °C until the analysis.

### 2.5. Sample Preparation and Analysis

For the analysis of hydrophilic extracts, 3 different methods were applied: untargeted GC-MS, targeted GC-MS/MS and targeted HILIC-MS/MS. The targeted GC-MS/MS method focused on 52 organic acid metabolites, while HILIC-MS/MS was applied to capture information on a certain set of 110 hydrophilic metabolites. In addition, an untargeted RP-LC-HRMS/MS method was applied for the global metabolic profiling of the combined hydrophilic and lipophilic extracts. In every case, QC samples were prepared by mixing equal volumes of the final extract and were analyzed every ten samples (or every five for RP-LC-HRMS/MS).

#### 2.5.1. GC-MS Analysis

For the GC-MS analysis, 10 μL of myristic acid-d27 (IS, 100 μg/mL) and 10 μL 4-phenylbutyric acid (IS, 100 μg/mL) were added to each 100 μL extract before evaporation. The dried residues were reconstituted in 65 μL of anhydrous pyridine, 2% MeOX, following incubation at 70 °C for 2 h. Then, the samples were allowed to cool at room temperature, and 125 μL MSTFA, 1% TMCS were added and incubated for 1 h at 70 °C for the formation of TMS derivatives. Subsequently, 10 μL of injection standard (N-pentadecane, 100 μg/mL) were added and the samples were split into 2 vials for the analysis in targeted and untargeted mode.

Analysis was performed on an EVOQ 456 GC-TQ-MS system (Bruker, Billerica, MA, USA) equipped with a CTC automatic sampler and PTV injector, controlled by Compass Hystar software. A 30 m HP-5 MS UI (Agilent J&W) column (0.25 mm, ID of 0.25 μm) was used, onto which 1 μL of sample was injected in splitless mode. The carrier gas was helium (99.999%), used at a flow rate of 1.5 mL/min. For the untargeted analysis, the initial inlet temperature was 110 °C for 1 min and then increased to 250 °C with a 720 °C/min rate, where it was held for 12 min. The temperature returned to the initial conditions for the remaining 23 min of the run. The column temperature was set to 70 °C for the initial 5 min before increasing to 100 °C (5 °C/min), then to 200 °C (10 °C/min), where it was held for 2 min and finally reaching 320 °C (15 °C/min) with a hold for 5 min. The total analysis time was 36 min. Electron ionization (EI) was applied, and ion source and transfer line temperatures were set to 230 °C and 250 °C respectively. Mass spectra were acquired over the range of 50–600 amu in full scan mode, with a solvent delay of 5.9 min.

#### 2.5.2. GC-MS/MS

For the targeted analysis, a method previously developed by our group [30] was applied. Mass spectrometer (MS) detection in MRM mode was performed, providing data for 52 organic acids in a total analysis time of 32.5 min EVOQ 456 GC-TQ-MS system, as above. The inlet and oven conditions are provided in detail in the previously published method [30].

#### 2.5.3. HILIC-MS/MS Analysis

Before the analysis, the dried residues were reconstituted in 70 μL of acetonitrile-water, 95:5 (*v*/*v*) and vortex-mixed. A hydrophilic interaction liquid chromatography (HILIC)—MS/MS method [31] was applied, focusing on 110 key hydrophilic metabolites. The separation was performed on an ACQUITY UPLC H-Class system using an ACQUITY UPLC BEH Amide column (Waters Ltd., Elstree, UK). A Xevo TQD mass spectrometer (Waters Corporation, Millford, MA, USA) was operated in MRM mode in both positive and negative electrospray ionization (ESI) modes. The mobile phase consisted of (A) acetonitrile-water, 95:5 (*v*/*v*) and (B) acetonitrile-water, 30:70 (*v*/*v*), both containing 10 mM ammonium formate at pH 6.

#### 2.5.4. RP-LC-HRMS/MS Analysis

Dried hydrophilic and lipophilic extracts were reconstituted in 200 µL methanol, vortex-mixed and shaken at 720 rpm for 45 min. The two extracts were subsequently combined, and then these samples were centrifuged at 16,000× *g* for 20 min. The supernatants were transferred to LC-MS vials.

The samples were analyzed in positive and negative ESI modes using data-independent acquisition (DIA)-MS/MS based on a previously published method [32]. The RP-LC-HRMS/MS system was comprised of a Nexera X2 LC coupled to an LCMS-9030 quadrupole-time of flight (Q-TOF) high resolution accurate mass MS system (Shimadzu Corporation, Kyoto, Japan). Chromatographic separations were performed using an ACQUITY UPLC BEH C18 column (1.7 µm, 2.1 × 100 mm, Waters Ltd., Elstree, UK) with a 35 min binary gradient of Solvent A (water with 0.1% formic acid) and Solvent B (acetonitrile with 0.1% formic acid). The method acquired a single TOF MS scan (*m*/*z* 65–1000) followed by 27 DIA-MS/MS mass scans over a mass range of *m*/*z* 40–1000. Each DIA-MS/MS mass scan had a precursor isolation width of 35 Da and a collision energy spread of 5–55 V, resulting in a cycle time of <1 s. This allowed the collection of fragmentation data for all masses in the spectra across the entire LC gradient.

### 2.6. Data Analysis

GC-MS untargeted data were initially processed with AMDIS software to achieve chromatographic peak deconvolution and identification. NIST (mainlib) and FIEHN libraries were used for the identification, applying simple mode with a minimum match factor of 50%. Peak areas of the compounds extracted by AMDIS were calculated using the Gavin3 script in MATLAB. RP-LC-HRMS/MS untargeted data were analyzed using LabSolutions Insight software (v3.8). GC-MS/MS data were processed using Bruker MSWS8 software, and peak areas were considered. HILIC-MS/MS data analysis was performed by TargetLynx (v4.1) (Waters, Milford, MA, USA), and peak areas were obtained.

For untargeted data (GC-MS and RP-LC-MRMS/MS), the metabolites that met the criteria of presence in 80% of the analyzed samples were considered. The quality of the data was also assessed through clustering of quality control (QC samples) in principal components analysis (PCA). In addition, a value of coefficient of variation (CV) of less than 30% in the QC samples was considered a threshold for all obtained metabolites.

For RP-LC-HRMS/MS data, an in-house built MS/MS library of metabolites and lipids commonly detected with this analytical method was used to identify metabolites. Library MS/MS spectra were acquired from authentic reference material where available.

For metabolites detected and identified by more than one of the four analytical methods (targeted HILIC-MS/MS, untargeted RP-LC-HRMS/MS, targeted GC-MS/MS and untargeted GC-MS), data from the most applicable method for that analyte was considered based on retention time and ionization. The final list of identified metabolites (Table S1) was considered for statistical analysis using both univariate and multivariate approaches.

Multivariate statistical analysis was performed using SIMCA 13.0.3 (UMETRICS AB, Umea, Sweden). Principal components analysis (PCA) was conducted, and the data were further processed by orthogonal-partial least squares discriminant analysis (OPLS-DA). Biomarker evaluation and statically significant features were highlighted by VIP (Variable Importance for the Projection) value ≥ 1.5; *p*(corr.) value ≥ ± 0.5 in UV scaling. Model quality was assessed by goodness of fit in the X (R2X) and Y (R2Y) variables and predictability (Q2YCV) and CV ANOVA analysis. Univariate statistical analysis was performed in Office365 MS Excel for the calculation of the two-tailed *t*-test with an unequal variance algorithm (*p* ≤ 0.05) and for the logarithmic value of fold change (Log2FC) to investigate the impact of each metabolite on the tested hypothesis. Pathway analysis was performed using MetaboAnalyst (V5.0) [33,34].

## 3. Results

Based on the microbiological findings from the fecal samples of G1, G2, G3, and G4 animals (Figure 1), it was verified that there was colonization of *C. difficile*. During the in vivo experiment, the general health of the animals was observed to be unaffected in terms of welfare; all infected mice showed only mild diarrhea. Body weight was monitored, and no statistically significant changes were observed in any animal. Nevertheless, it was ascertained that brain metabolism was perturbed, as the brain metabolic profiles of mice infected with *C. difficile* (G1–G4) were found to be altered in comparison to the controls (G5). 

Brain extracts were analyzed using four different analytical methods: targeted HILIC-LC-MS/MS, untargeted RP-LC-HRMS/MS, targeted GC-MS/MS and untargeted GC-MS. Information on a wide variety of heterogeneous metabolites of brain metabolome could be captured covering important metabolic pathways.

Compilation of data from all four methods and after applying data quality filters (see Section 2.6) resulted in a total of 217 unique metabolites identified in the brain extracts (Table S1). These comprised glycerophospholipids (33%), glycerolipids (12%), amino acids, peptides, and analogues (13%), carbohydrates and carbohydrate conjugates (7%), and fatty acids and conjugates (7%). Univariate and multivariate statistical analyses revealed metabolites that were significantly differentiated in the brain extracts among the studied groups as a result of infection and treatments.

### 3.1. C. difficile Infection

**Figure 2:**
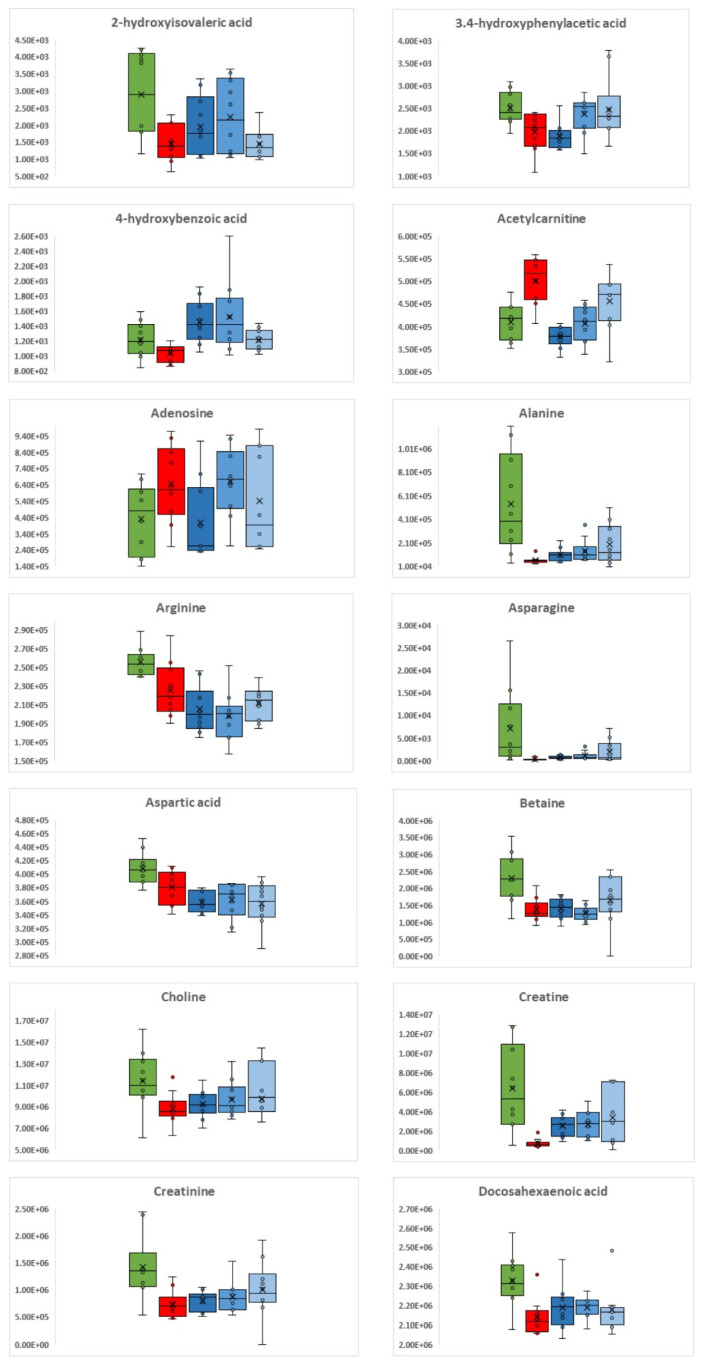

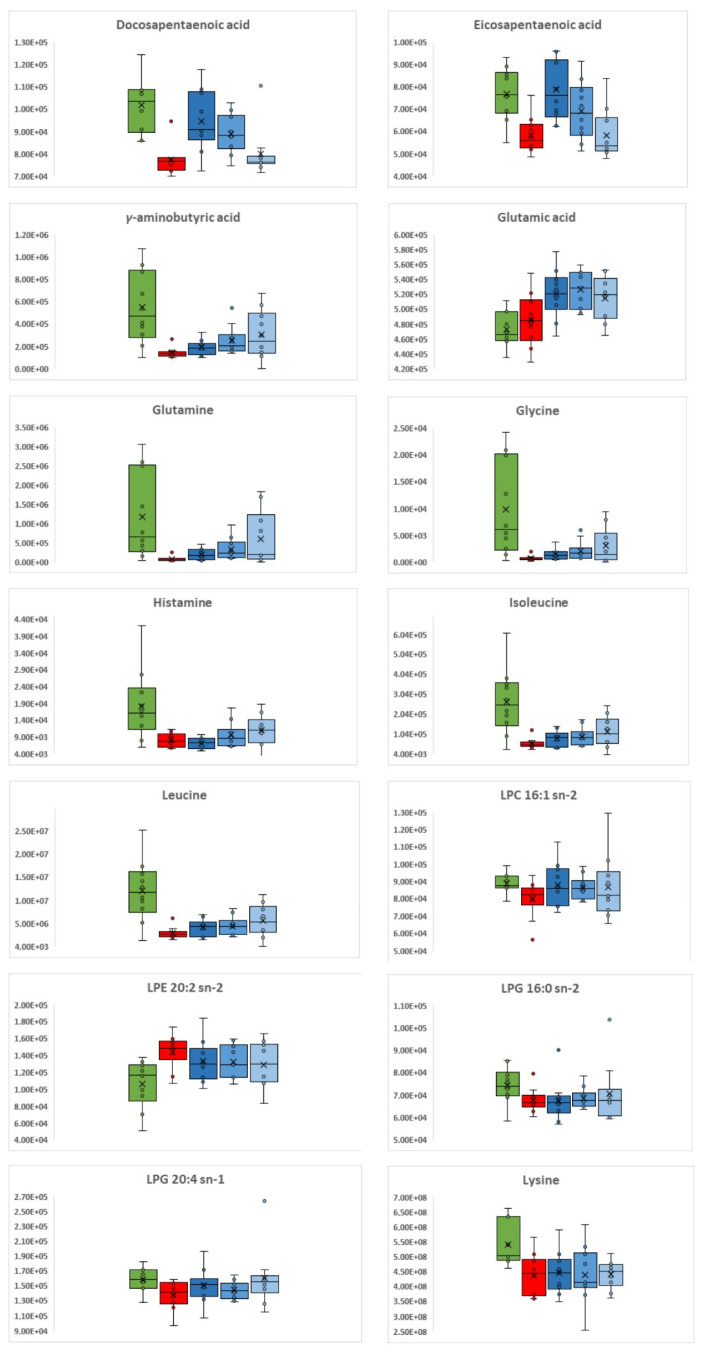

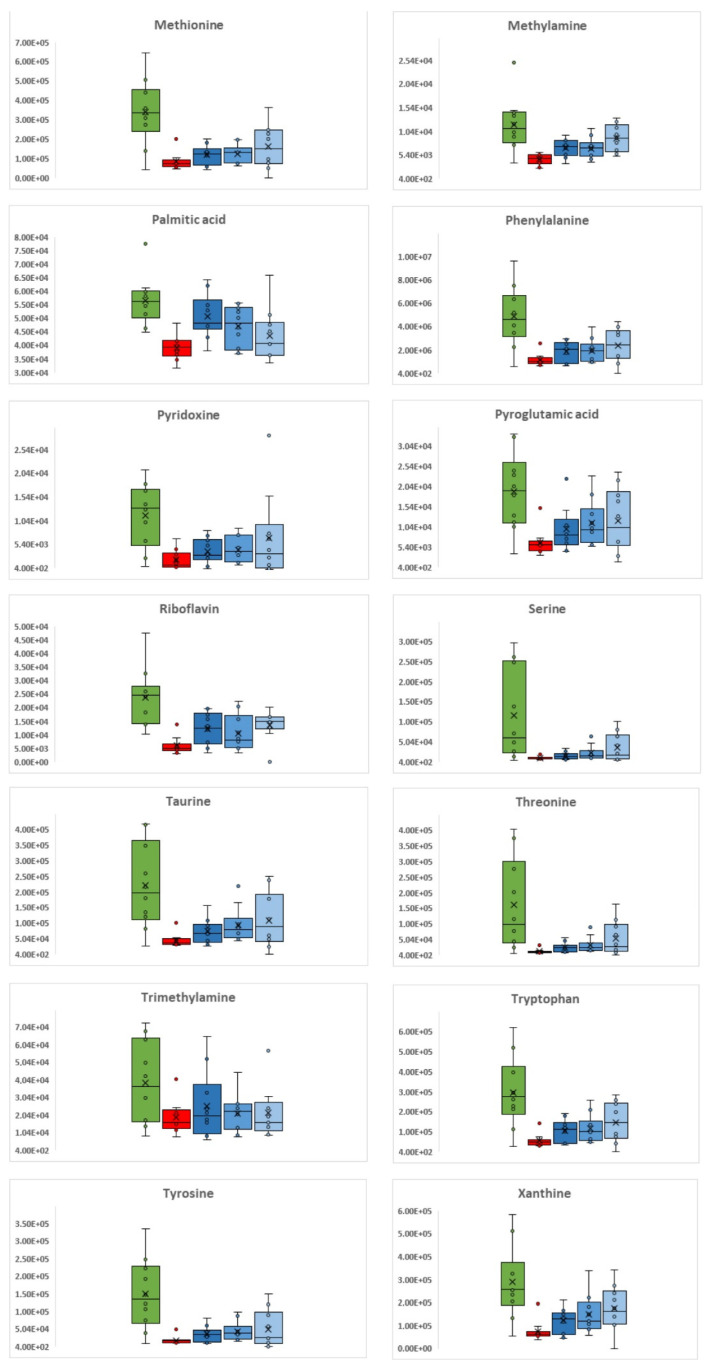
Box plots of 42 representative brain metabolites (alphabetically ordered) that were found to be significantly deregulated after *C. difficile* colonization (G4 in red) in comparison to the control group (G5 in green). The effect of their levels can also be seen in the three groups of treatment, colored in dark blue for metronidazole (G1), in blue for probiotics (G2), and in light blue for FMT (G3).

Comparing the metabolic profiles of the control group (G5) with the group of mice that were infected but not subjected to any therapeutic treatment (G4), approximately 40% (91 out of 217) of the metabolites showed significantly altered levels (*p* ≤ 0.05). Among the metabolites that differed significantly, those with a higher logarithmic value of fold change (|Log2FC|≥ 1), were the most affected with infection. All these metabolites were found to be decreased in the infected mice compared to the controls and mostly comprise amino acids but also vitamins, namely nicotinic acid (which showed the highest decrease), pyridoxine, riboflavin, xanthine, acids such as glyceric, *γ*-aminobutyric and pyroglutamic and amines such as histamine, tryptamine, methylamine, and trimethylamine. This indicates that key compounds in brain metabolism are downregulated. The differentiation in the intensities of the most significantly dysregulated metabolites by the infection with *C. difficile* can be seen in the box plots of Figure 2, where controls (G5) are indicated in green and infected-untreated (G4) in red.

In Table S2, the calculated *p*-values and log2FC for all metabolites detected can be found, given for each pairwise comparison of the five studied groups. In this table, the numbers are highlighted for statistically significant (*p* ≤ 0.05 and |Log2FC| ≥ 1) differentiations.

### 3.2. C. difficile Therapeutic Treatment

When the condition of the animals subjected to therapeutic treatment was considered, it was observed that for G1 (metronidazole), there was continuation of mild diarrhea, while the other two groups were free of symptoms. Alterations in the brain metabolic phenotypes that were captured by the applied methods were observed in all three treatment groups compared to the untreated group (G4); however, the response to each treatment was different. When compared to the infected and untreated group (G4), the highest number of metabolites were significantly altered in the metronidazole-treated group—G1 (60 out of 217), followed by the probiotic treatment group—G2 (48 out of 217). The FMT group—G3 had the lowest number of significantly different metabolites (28/217), but the effects appeared to be more intense, with the Log2FC values being much higher between this treatment and the untreated group (G4) than with any other treatment compared to G4. Regarding the trend, almost all metabolites increased upon treatment, except for trimethylamine in the metronidazole- and probiotic-treated groups (G1 and G2) and 3-hydroxybutyric and adenosine in the metronidazole-treated group (G1). In general, the response observed in G1 and G2, corresponding to antibiotic and probiotic treatments, was more similar to each other in comparison with the response to FMT treatment. There were more common metabolites exhibiting similar trends in G1 and G2, including 1-Monoacylglycerol [MG 14:0 (1)], 1-Monooleoylglycerol [MG 18:1 (1)], 3-hydroxybutyric acid, 4-hydroxybenzoic acid, acetylcarnitine, alanine, asparagine, and creatine. On the other hand, FMT caused increases in different metabolites, such as 2-ketogloutaric acid, 4-hydroxybenzoic acid, and 5-hydroxy indole-3-acetic acid.

Many of the metabolites found to be increased in the three treatment groups were those that were decreased by the infection (G4). This suggests that treatment leads to a reversing trend in many of the metabolites dysregulated by the infection (decrease in G4 in comparison to the control G5). The levels of amino acids were increased by all three treatments relative to the infected and untreated group, with glutamine exhibiting the greatest |Log2FC|. Other metabolites that showed a reversed trend in all treatments were xanthine, creatine, and methylamine. In some cases, only certain treatments were able to reverse the effects of the treatment. For example, docosapentaenoic acid and eicosatrienoic acid, which had a Log2FC decrease of 0.4 with infection (G4 compared to G5), showed an increase only after treatment with metronidazole (G1) or probiotics (G2). The effects of the three treatments on metabolite levels can be seen in Figure 2, where the intensities of the representative metabolites are illustrated as box plots.

When the treated groups were compared to the control group (G5), more brain metabolites were found to differ significantly in the antibiotic and probiotic groups (G1 and G2) than in the FMT group G3 (96 and 87 out of 217, in contrast to 53 out of 217 for G3). This indicates that the FMT group’s brain metabolic phenotype is the more similar one to the controls 10 days after treatment, suggesting that the respective intervention may be more effective at restoring the healthy phenotype. Around seventy percent of the differentiated metabolites are decreased in groups G1, G2, and G3 compared to controls (G5).

Many altered metabolites were common in the three treated groups, including serine, glycine, arginine, and inosine, with nicotinic acid characteristically being the one with the most noteworthy log2FC decrease in all three groups. In Figure 2, where representative metabolites from those that showed a significant alteration with infection are plotted, the reversing trend in the treated animals for these metabolites can be seen. It is important to point out that several metabolites, such as arginine, betaine, choline, glutamine, and glycine, decreased due to the infection, and this effect was markedly reversed in the G3 group. Although this was also observed in groups G1 and G2, the effect was not as pronounced.

**Figure 3:**
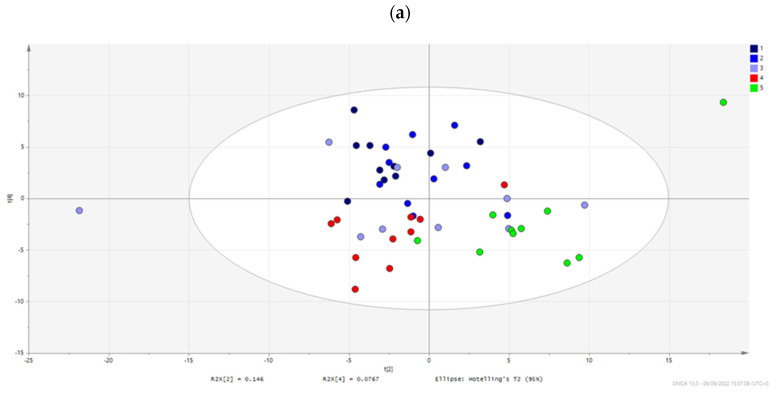

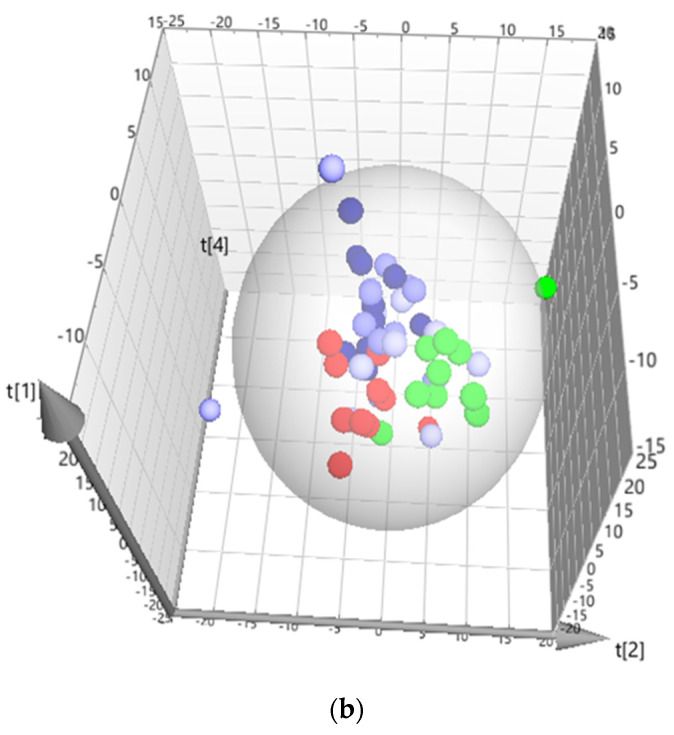
2D (**a**) and 3D (**b**) PCA score plots of the model constructed for all studied groups. Infected mice (G4), colored in red, are clustered separately from controls (G5), which are colored in green. The three treatment groups are shown in dark blue (G1), blue (G2), and light blue (G3).

The approach of multivariate statistics was also used for the evaluation of the data to reveal the potential biological significance of the studied interventions. The constructed unsupervised principal component analysis (PCA) score plot (Figure 3) showed a tendency in the clustering of the samples according to the treatment, which was more pronounced when data were analyzed by supervised orthogonal partial least squares (OPLS), as presented in Figure S1.

**Figure 4:**
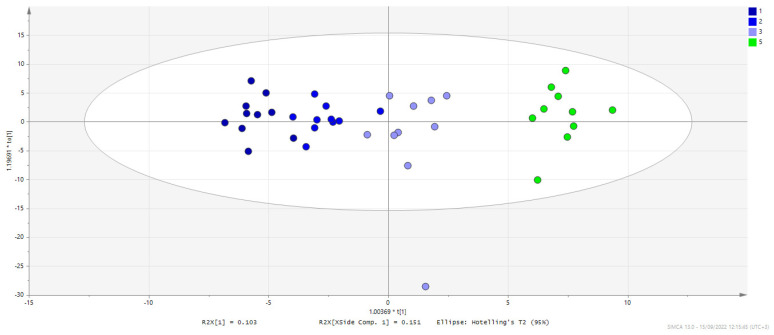
Score plot showing the projection of samples from the three treated groups G1 (in dark blue), G2 (in blue), G3 (in light blue), and the controls G5 (in green) based on the constructed OPLS model. Brain samples of metronidazole and probiotic treatment are shown to cluster on the left side of the eclipse, whereas FMT samples are plotted closer to the control group, projected into the right end.

Based on PCA, it can be observed that G4 is clustered away from G5 (controls), indicating a differentiation of the metabolic phenotype with the *C. difficile* infection, as shown also by univariate statistical analysis. Therapeutic treatments do not seem to be clearly distinguished based on the acquired metabolome. When supervised models are constructed, a trend is revealed, i.e., the three groups are separately clustered, while FTM treatment is the one that drives the metabolic profile closer to the controls, whereas the G1 cluster is in the far end from G5. This can be seen in Figure 4, where the treated groups (in blue) and controls (in green) are plotted. However, it is clear that no treatment fully recovered the brain metabolic profile to the healthy control phenotype (G5), which indicates a remaining disrupted metabolism 10 days after therapeutic treatment in all cases. Pairwise discriminant analysis indicated differentiation between each treatment group (metronidazole-G1, probiotic-G2, or FMT-G3 and the uninfected control group-G5), with the greatest difference arising from the metronidazole treatment (Figure 4 and Table 1). Table S3 details the extracted p(corr) and VIP (Variable Importance for the Projection) values from each statistically significant pairwise comparison based on OPLS-DA; VIP values ≥ 1.50 are marked in red. Table S4 summarizes the most significant features from both univariate and multivariate analyses, highlighting metabolites that meet the criteria of *p* value ≤ 0.05 and VIP value ≥ 1.5.

## 4. Discussion

Both bacterial infection and treatments for bacterial infection can have a profound effect on the gut microbiome, which may in turn affect the brain metabolome via gut–brain axis communication. Herein, the effects of CDI and three common treatments on the brain metabolome in a murine model are discussed for the first time. We investigated the metabolic perturbations induced by infection and evaluated the therapeutic effects of the first-line prescriptive antibiotic treatment, probiotics, and FMT as the most promising treatment in our CDI murine model.

Over the last decade, CDI pathology has been explored using metabolomics applied mainly to fecal samples [1,5,22,35,36,37,38,39], and to a smaller extent to urine [40] and gastrointestinal tract tissues [41]. The purpose was to find biomarkers of primary and recurrent CDI in patients and animal models and to provide evidence of metabolic perturbation induced by *C. difficile* colonization [20,22,35,36].

In many studies, the association of primary and secondary bile acid composition in fecal samples with the CDI mechanism has been explored [3,6,18,22,42,43,44].

Murine studies have demonstrated that CDI decreases the levels of several amino acids and increases metabolic products, such as 5-aminovalerate, since *C. difficile* metabolizes a plethora of carbon substrates, including proline, glycine, and branched-chain amino acids (BCAAs), via Stickland fermentation [36].

It has also been shown that after antibiotic administration, *C. difficile* exploits the abundance of primary bile acid taurocholate and carbon sources such as mannitol, fructose, sorbitol, raffinose, and stachyose for its germination and growth [22].

In a human study, a distinct fecal metabolome of hospitalized patients with CD diarrhea has been reported as sphingosine, chenodeoxycholic acid, phenylalanine, lysophosphatidylcholine (C16:0), and propylene glycol stearate being increased, while fatty amide, glycochenodeoxycholic acid, tyrosine, linoleyl carnitine, and sphingomyelin were found to decrease in CDI patients [5].

**Figure 5:**
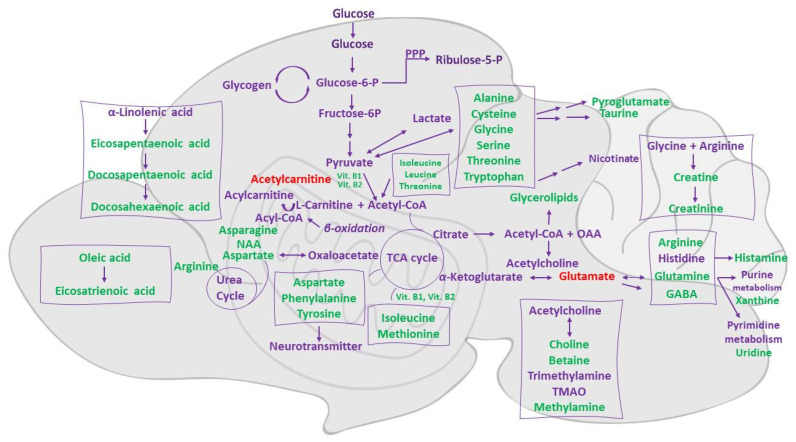
Graphical illustration of dysregulated metabolites in the brain and related biochemical pathways after *C. difficile* colonization. Metabolites in green decreased and in red increased. Thiamine (Vit B1) and Riboflavin (Vit B2).

In the present study, a comparison of brain metabolic profiles with and without CDI indicated statistically significant decreases in a number of metabolites with important roles in a healthy phenotype, as described below. Many amino acids, such as leucine, valine, glutamic acid, aspartic acid, and tyrosine, were found to be decreased (Figure 5). The BCAAs leucine, isoleucine, and valine are major nitrogen donors supplying glutamate synthesis. Glutamate and aspartate are the main excitatory neurotransmitters in the brain [45] and the first is the precursor of *γ*-aminobutyric acid (GABA) [46], while glycine is the major inhibitory neurotransmitter. Serotonin (a tryptophan-derived product), histamine, and the metabolic products of tyrosine (dopamine and norepinephrine) are neurotransmitters with significant roles in brain function. The plasma levels of glutamine (a precursor of glutamate and aspartate), of BCAAs, and of serine (a precursor of glycine and D-serine) affect neurotransmission via glutamate, aspartate, and glycine synthesis and the competitive transport of tryptophan and tyrosine across the blood–brain barrier [47].

In addition, important vitamins, such as pyridoxine and riboflavin, were decreased. Pyridoxine is a cofactor of the enzymes required for the synthesis of GABA in the brain [48]. Along with riboflavin, it restores dopamine levels and reduces oxidative stress in the brains of rats [49]. Taurine, an important neurodevelopmental modulator with numerous cellular functions that has shown a protective role against glutamate-mediated neuronal cell death [50,51] was also decreased. All of the above indicate that significant metabolic pathways for brain function were affected (Figure S2).

It has been suggested that CDI perturbs the gut microbiome and consequently disrupts the metabolism of primary bile acids to secondary bile acids, which are crucial for germination and growth of *C. difficile,* and that these can be normalized with FMT treatment. Thus, FMT has been proposed as a promising treatment for refractory recurrent CDI, which usually follows antibiotic administration. This, however, has only been investigated in fecal samples. For example, in post-FMT fecal samples of CDI patients, mostly secondary bile acids were detected, in contrast to high concentrations of primary bile acids and bile salts in pre-FMT patient’s samples [6].

In our study, it was shown that FMT provides a brain metabolome closer to the controls in comparison to the other two treatments. Interestingly, between the two conservative treatments, FMT was shown to “restore” the brain metabolome more efficiently than probiotics. This could be due to a wider variety of bacteria being imported into the gastrointestinal track of the animals via FMT, in comparison to the limited number of strains (eleven) by probiotic treatment. In addition, it seems that FMT leads to a more distinct metabolic profile compared to the other two therapies when their effect on infected mice was studied. Alterations in characteristic metabolites, such as N-acetyl aspartate and LPGs, were observed that were not altered by the other treatments. Interestingly, the effect of probiotics has shown a similar trend to that of metronidazole. In any case, however, it should be stressed that none of the approaches seems to bring back the equilibrium in the brain metabolome, and further studies are needed in this direction.

Confirming our initial hypothesis, the alterations of gut symbiome triggered by the interventions (data not shown here will be reported in a following manuscript) were probably responsible for brain differentiated metabolites through the gut–brain metabolic interaction. To our knowledge, this is the first study to describe the effect of CDI in the brain metabolome, building on previous knowledge from published data on fecal samples to better understand the effects of CDI and its treatments. The origin of differentiated metabolites in fecal samples due to CDI does not necessarily match those in the brain metabolome since there are enzymatic and physiological differences. We aim to investigate this metabolic interaction in a forthcoming article, where we will discuss the results of the metabolomics-based analysis of fecal samples collected throughout the experiment (with the same animals), as well as the relations of the affected fecal metabolome with the altered composition of the gut microbial community.

## 5. Conclusions

*Clostridium difficile* colonization after antibiotic administration affected the brain metabolic phenotype by disrupting the equilibrium of the gut microbiota. This may have a profound effect on brain metabolism since the differentiated metabolites are involved in significant biochemical pathways.

Although the administration of metronidazole is used as the standard treatment, did not show to regulate the altered brain metabolome. Probiotics and, even more, fecal microbiota transplantation showed an improving effect on the brain metabolic phenotypes, although none of the three applied treatments was able to fully restore the healthy metabolome.

The mechanism of action of the antibiotic-induced *C. difficile* infection is still challenging; thus, further studies are required for the elucidation of its effect on the brain metabolome through the gut–brain interaction.

## Figures and Tables

**Table 1:** Characteristics of the constructed unsupervised and supervised models.

Model	Type	N	R2X(cum)	R2Y(cum)	Q2(cum)	CV ANOVA
1,2,3,4,5	PCA-X	50	0.557		0.335	
1,2,3,4,5	OPLS	50	0.481	0.946	0.834	1.139E-08
1,2,3,5	OPLS	40	0.518	0.966	0.898	3.246E-08
1,4	OPLS-DA	20	0.610	0.985	0.854	3.587E-04
1,5	OPLS-DA	20	0.625	0.991	0.909	2.453E-05
2,4	OPLS-DA	20	0.543	0.987	0.789	2.702E-03
2,5	OPLS-DA	20	0.526	0.989	0.832	7.549E-04
3,4	OPLS-DA	20	0.616	0.962	−0.041	1.000E+00
3,5	OPLS-DA	20	0.577	0.972	0.824	9.762E-04
4,5	OPLS-DA	20	0.600	0.990	0.840	5.839E-04

## Data Availability

Not applicable.

## Supplementary Materials

The following supporting information can be downloaded at: https://www.mdpi.com/article/10.3390/metabo12111039/s1, Table S1: Unique metabolites identified in brain extracts. Compound names, chemical formulae, method of detection, metabolomics standards initiative (MSI) level of identification, and chemical taxonomy (class and sub-class) are given for each. In the case where a metabolite was detected by more than one method, the most representative method for that compound was chosen for reporting and statistical analysis; Table S2: Calculated *p*-values and Log2FC values from pairwise comparisons of each treatment group G1: metronidazole; G2: probiotics; G3: fecal microbiota transplantation with either the *C. difficile* infected and untreated group G4 or the uninfected and untreated control group G5.Values highlighted in red correspond to *p* ≤ 0.05 or |Log2FC| ≥ 1; Table S3: Extracted p(corr) and VIP values from each statistically significant pairwise comparison based on OPLS discriminant analysis. VIP values ≥ 1.50 are marked in red; Table S4a,b: Statistically significant metabolites that met the criteria of *p* value ≤ 0.05 and VIP value ≥ 1.5 from univariate and multivariate statistics for pairwise comparisons of the studied groups. In bold are compounds in common ((a) comparisons 1v4, 2v4, 4v5 and (b) comparisons 1v5, 2v5, 3v5). Figure S1. OPLS score plot of the model constructed for the five studied groups. Infected mice (G4) are colored in red, and controls (G5) are colored in green. The three treatment groups are shown in dark blue (G1), blue (G2), and light blue (G3). Figure S2. Metabolic pathways highlighted by MetaboAnalyst 5.0 have been affected by CDI based on the differentiated metabolites in the brain. The *x*-axis represents the values of pathway impact extracted from the pathway topological analysis. The *y*-axis represents the -log of the *p*-value derived from the pathway enrichment analysis. The pathways with higher scores for both values are illustrated on the top right region of the contracted plot.
