# Supplementary material for: Metabolic Phenotyping Study of Mouse Brain Following Microbiome Disruption by C. difficile Colonization
Source: Metabolites. 2022 Oct 28;12(11):1039. doi: 10.3390/metabo12111039 (PMC9696486; doi:10.3390/metabo12111039)
Supplement: Supplementary file 1 [file metabolites-12-01039-s001.zip › metabolites-1972086-supplementary.pdf]

SUPPLEMENTARY MATERIAL

Metabolic phenotyping study of mouse brain following microbiome disruption by *C. Difficile* colonisation

Olga Deda[1,2]*, Melina Kachrimanidou[3], Emily G Armitage[4], Thomai Mouskeftara[1,2], Neil J Loftus[4], Ioannis Zervos[5], Ioannis Taitzoglou[5], Helen Gika[1,2]*

[1]Laboratory of Forensic Medicine & Toxicology, Department of Medicine, Aristotle University of Thessaloniki, 54124 Thessaloniki, Greece
[2] Biomic AUTh, Center for Interdisciplinary Research and Innovation (CIRI-AUTH), Balkan Center B1.4, 10th km Thessaloniki-Thermi Rd, P.O. Box 8318, GR 57001 Thessaloniki, Greece
[3]1st Laboratory of Microbiology, Department of Medicine, Aristotle University of Thessaloniki, 54124 Thessaloniki, Greece
[4]Shimadzu Corporation, Manchester M17 1GP, U.K
[5]Laboratory of Animal Physiology, Faculty of Veterinary Medicine, School of Health Sciences, Aristotle University of Thessaloniki, 54124 Thessaloniki, Greece
*Corresponding Authors: Olga Deda, Helen Gika

Table S1. Unique metabolites identified in brain extracts. Compound names, chemical formulae, method of detection, metabolomics standards initiative (MSI) level of identification and chemical taxonomy (class and sub-class) are given for each. In the case where a metabolite was detected by more than one method, the most representative method for that compound was chosen for reporting and statistical analysis.

	Compound	Method	Ionization	RT	Chemical Formula	MSI level identification	Class	Subclass
1	1,5-Anhydroglucitol	GC-MS	EI+	21.9	$C_6H_{12}O_5$	2	Organooxygen compounds	Carbohydrates and carbohydrate conjugates
2	2,3-Butanediol	GC-MS	EI+	9.3	$C_4H_{10}O_2$	2	Organooxygen compounds	Alcohols and polyols
3	2-Hydroxybutyric acid	GC-MS/MS	EI+	8.6	$C_4H_8O_3$	1	Hydroxy acids and derivatives	Alpha hydroxy acids and derivatives
4	2-Ketoglutaric acid	GC-MS/MS	EI+	20.1	$C_5H_6O_5$	1	Keto acids and derivatives	Gamma-keto acids and derivatives
5	2-Hydroxyisovaleric acid	GC-MS/MS	EI+	9.5	$C_5H_{10}O_3$	1	Fatty Acyls	Fatty acids and conjugates
6	3,4-Dihydroxyphenylacetic acid	GC-MS/MS	EI+	25.5	$C_8H_8O_4$	1	Phenols	Benzenediols
7	3-Hydroxy-3-methylglutaric acid	GC-MS/MS	EI+	20.6	$C_6H_{10}O_5$	1	Fatty Acyls	Fatty acids and conjugates
8	3-Hydroxybutyric acid	GC-MS/MS	EI+	9.4	C4H8O_3	1	Hydroxy acids and derivatives	Beta hydroxy acids and derivatives
9	3-Methyl-2-oxovaleric acid	GC-MS/MS	EI+	9.9	$C_6H_{10}O_3$	1	Keto acids and derivatives	Short-chain keto acids and derivatives
10	4-Hydroxybenzoic acid	GC-MS/MS	EI+	20.9	$C_7H_6O_3$	1	Benzene and substituted derivatives	Benzoic acids and derivatives
11	4-Hydroxyphenyllactic acid	GC-MS/MS	EI+	26.9	$C_9H_{10}O_4$	1	Phenylpropanoic acids	
12	5-Hydroxyindole-3-acetic	GC-MS/MS	EI+	31.5	$C_{10}H_9NO_3$	1	Indoles and derivatives	Indolyl carboxylic acids and derivatives
13	5-Methylthioadenosine	RP-LC-HRMS/MS	ESI+	2.6	$C_{11}H_{15}N_5O_3S$	1	5'-deoxyribonucleosides	5'-deoxy-5'-thionucleosides
14	Acetylcarnitine	RP-LC-	ESI+	0.6	$C_9H_{17}NO_4$	1	Fatty Acyls	Fatty acid esters

		HRMS/MS						
15	Aconitic acid	GC-MS/MS	EI+	23.9	$C_6H_6O_6$	1	Carboxylic acids and derivatives	Tricarboxylic acids and derivatives
16	Adenosine	RP-LC-HRMS/MS	ESI+	1.3	$C_{10}H_{13}N_5O_4$	1	Purine nucleosides	
17	Adenosine monophosphate	RP-LC-HRMS/MS	ESI+	0.6	$C_{10}H_{14}N_5O_7P$	1	Purine nucleotides	Purine ribonucleotides
18	Adrenic acid	RP-LC-HRMS/MS	ESI+	12.7	$C_{22}H_{36}O_2$	2	Fatty Acyls	Fatty acids and conjugates
19	Alanine	HILIC-MS/MS	ESI+	16.0	$C_3H_7NO_2$	1	Carboxylic acids and derivatives	Amino acids, peptides, and analogues
20	alpha-D-Lyxopyranose	GC-MS	EI+	22.2	$C_5H_{10}O_5$	2	Organooxygen compounds	Carbohydrates and carbohydrate conjugates
21	alpha-Linolenic acid	GC-MS	EI+	27.2	$C_{18}H_{30}O_2$	2	Fatty Acyls	Lineolic acids and derivatives
22	alpha-Tocopherol	GC-MS	EI+	31.3	$C_{29}H_{50}O_2$	2	Prenol lipids	Quinone and hydroquinone lipids
23	Arabinitol	GC-MS	EI+	20.4	$C_5H_{12}O_5$	2	Organooxygen compounds	Carbohydrates and carbohydrate conjugates
24	Arachidonic acid	GC-MS	EI+	26.3	$C_{20}H_{32}O_2$	2	Fatty Acyls	Fatty acids and conjugates
25	Arachidonoylcarnitine	RP-LC-HRMS/MS	ESI+	6.7	$C_{27}H_{46}NO_4$	2	Fatty Acyls	Fatty acid esters
26	Arginine	RP-LC-HRMS/MS	ESI+	0.5	$C_6H_{14}N_4O_2$	1	Carboxylic acids and derivatives	Amino acids, peptides, and analogues
27	Asparagine	HILIC-MS/MS	ESI+	18.2	$C_4H_8N_2O_3$	1	Carboxylic acids and derivatives	Amino acids, peptides, and analogue
28	Aspartic acid	RP-LC-HRMS/MS	ESI+	0.6	$C_4H_7NO_4$	1	Carboxylic acids and derivatives	Amino acids, peptides, and analogues
29	Azelaic acid	GC-MS/MS	EI+	25.7	$C_9H_{16}O_4$	1	Fatty Acyls	Fatty acids and conjugates
30	Benzoic acid	GC-MS/MS	EI+	11.5	$C_7H_6O_2$	1	Benzene and substituted derivatives	Benzoic acids and derivatives
31	beta-Arabinopyranose	GC-MS	EI+	23.8	$C_5H_{10}O_5$	2	Organooxygen compounds	Carbohydrates and carbohydrate conjugates
32	Betaine	HILIC-MS/MS	ESI+	12.8	$C_5H_{12}NO_2$	1	Carboxylic acids and derivatives	Amino acids, peptides, and analogues
33	Carnitine	RP-LC-HRMS/MS	ESI+	0.6	$C_7H_{15}NO_3$	1	Organonitrogen compounds	Quaternary ammonium salts
34	Cholesterol	GC-MS	EI+	31.4	$C_{27}H_{46}O$	2	Steroids and steroid derivatives	Cholestane steroids
35	Choline	HILIC-MS/MS	ESI+	7.0	$C_5H_{14}NO$	1	Organonitrogen compounds	Quaternary ammonium salts
36	Citramalic acid	GC-MS/MS	EI+	17.6	$C_5H_8O_5$	1	Fatty Acyls	Fatty acids and conjugates
37	Citric acid	GC-MS/MS	EI+	25.5	$C_6H_8O_7$	1	Carboxylic acids and derivatives	Tricarboxylic acids and derivatives
38	Citrulline	RP-LC-HRMS/MS	ESI+	0.6	$C_6H_{13}N_3O_3$	1	Carboxylic acids and derivatives	Amino acids, peptides, and analogues

39	Creatine	HILIC-MS/MS	ESI+	16.3	$C_4H_9N_3O_2$	1	Carboxylic acids and derivatives	Amino acids, peptides, and analogues
40	Creatinine	HILIC-MS/MS	ESI+	4.8	$C_4H_7N_3O$	1	Carboxylic acids and derivatives	Amino acids, peptides, and analogues
41	Cysteine	GC-MS	EI+	18.5	$C_3H_7NO_2S$	2	Carboxylic acids and derivatives	Amino acids, peptides, and analogues
42	Cytidine	RP-LC-HRMS/MS	ESI+	0.9	$C_9H_{13}N_3O_5$	1	Pyrimidine nucleosides	
43	Cytidine monophosphate	RP-LC-HRMS/MS	ESI-	0.6	$C_9H_{14}N_3O_8P$	1	Pyrimidine nucleotides	Pyrimidine ribonucleotides
44	Cytosine	RP-LC-HRMS/MS	ESI+	0.6	$C_4H_5N_3O$	1	Diazines	Pyrimidines and pyrimidine derivatives
45	D-Erythrose	GC-MS	EI+	23.0	$C_4H_8O_4$	2	Organooxygen compounds	Carbohydrates and carbohydrate conjugates
46	D-Fructose	GC-MS	EI+	22.4	$C_6H_{12}O_6$	2	Organooxygen compounds	Carbohydrates and carbohydrate conjugates
47	D-Fucitol	GC-MS	EI+	20.6	$C_6H_{14}O_5$	2	Organooxygen compounds	Carbohydrates and carbohydrate conjugates
48	D-Glucose	GC-MS	EI+	22.9	$C_6H_{12}O_6$	2	Organooxygen compounds	Carbohydrates and carbohydrate conjugates
49	Dimethylamine	HILIC-MS/MS	ESI+	8.2	C_2H_7N	1	Organonitrogen compounds	Amines
50	D-Lyxose	GC-MS	EI+	20.1	$C_6H_{10}O_7$	2	Organooxygen compounds	Carbohydrates and carbohydrate conjugates
51	D-Mannitol	GC-MS	EI+	23.4	$C_6H_{14}O_6$	2	Organooxygen compounds	Carbohydrates and carbohydrate conjugates
52	Docosahexaenoic acid	RP-LC-HRMS/MS	ESI+	10.5	$C_{22}H_{32}O_2$	1	Fatty Acyls	Fatty acids and conjugates
53	Docosapentaenoic acid	RP-LC-HRMS/MS	ESI+	11.3	$C_{22}H_{34}O_2$	2	Fatty Acyls	Fatty acids and conjugates
54	Docosenamide	RP-LC-HRMS/MS	ESI+	15.6	$C_{22}H_{43}NO$	2	Fatty Acyls	Fatty amides
55	Eicosapentaenoic acid	RP-LC-HRMS/MS	ESI+	9.7	$C_{20}H_{30}O_2$	2	Fatty Acyls	Fatty acids and conjugates
56	Eicosatrienoic acid	RP-LC-HRMS/MS	ESI+	11.9	$C_{20}H_{34}O_2$	2	Fatty Acyls	Fatty acids and conjugates
57	Ethanolamine	GC-MS	EI+	14.1	C_2H_7NO	2	Organonitrogen compounds	Amines
58	Fumaric acid	GC-MS/MS	EI+	14.3	$C_4H_4O_4$	1	Carboxylic acids and derivatives	Dicarboxylic acids and derivatives
59	Galactose	GC-MS	EI+	25.6	$C_6H_{12}O_6$	2	Organooxygen compounds	Carbohydrates and carbohydrate conjugates
60	Glutamic acid	RP-LC-HRMS/MS	ESI+	0.6	$C_5H_9NO_4$	1	Carboxylic acids and derivatives	Amino acids, peptides, and analogues
61	Glutamine	HILIC-MS/MS	ESI+	17.8	$C_5H_{10}N_2O_3$	1	Carboxylic acids and derivatives	Amino acids, peptides, and analogues

62	Glutaric acid	GC-MS	EI+	18.7	$C_5H_8O_4$	2	Carboxylic acids and derivatives	Dicarboxylic acids and derivatives
63	Glyceric acid	GC-MS/MS	EI+	14.1	$C_3H_6O_4$	1	Organooxygen compounds	Carbohydrates and carbohydrate conjugates
64	Glycerol	GC-MS	EI+	29.5	$C_3H_8O_3$	2	Organooxygen compounds	Carbohydrates and carbohydrate conjugates
65	Glycine	HILIC-MS/MS	ESI+	17.0	$C_2H_5NO_2$	1	Carboxylic acids and derivatives	Amino acids, peptides, and analogues
66	Glycolic acid	GC-MS/MS	EI+	7.2	$C_2H_4O_3$	1	Hydroxy acids and derivatives	Alpha hydroxy acids and derivatives
67	Guanosine	RP-LC-HRMS/MS	ESI+	1.3	$C_{10}H_{13}N_5O_5$	1	Purine nucleosides	
68	Histamine	HILIC-MS/MS	ESI+	13.7	$C_5H_9N_3$	1	Organonitrogen compounds	Amines
69	Histidine	RP-LC-HRMS/MS	ESI+	0.52	$C_6H_9N_3O_2$	1	Carboxylic acids and derivatives	Amino acids, peptides, and analogues
70	Homovallinic acid	GC-MS/MS	EI+	24.2	$C_9H_{10}O_4$	1	Phenols	Methoxyphenols
71	Hypoxanthine	HILIC-MS/MS	ESI+	4.8	$C_5H_4N_4O$	1	Imidazopyrimidines	Purines and purine derivatives
72	Inosine	RP-LC-HRMS/MS	ESI+	1.4	$C_{10}H_{12}N_4O_5$	1	Purine nucleosides	
73	Isoleucine	HILIC-MS/MS	ESI+	13.3	$C_6H_{13}NO_2$	1	Carboxylic acids and derivatives	Amino acids, peptides, and analogues
74	Lactic acid	GC-MS/MS	EI+	6.9	$C_3H_6O_3$	1	Hydroxy acids and derivatives	Alpha hydroxy acids and derivatives
75	Leucine	HILIC-MS/MS	ESI+	13.4	$C_6H_{13}NO_2$	1	Hydroxy acids and derivatives	Alpha hydroxy acids and derivatives
76	LPC 14:0 sn-1	RP-LC-HRMS/MS	ESI+	5.9	$C_{22}H_{46}NO_7P$	2	Glycerophospholipids	Glycerophosphocholines
77	LPC 14:0 sn-2	RP-LC-HRMS/MS	ESI+	5.6	$C_{22}H_{46}NO_7P$	2	Glycerophospholipids	Glycerophosphocholines
78	LPC 16:0 sn-1	RP-LC-HRMS/MS	ESI+	7.5	$C_{24}H_{50}NO_7P$	2	Glycerophospholipids	Glycerophosphocholines
79	LPC 16:0 sn-2	RP-LC-HRMS/MS	ESI+	7.1	$C_{24}H_{50}NO_7P$	2	Glycerophospholipids	Glycerophosphocholines
80	LPC 16:1 sn-1	RP-LC-HRMS/MS	ESI+	6.3	$C_{24}H_{48}NO_7P$	2	Glycerophospholipids	Glycerophosphocholines
81	LPC 16:1 sn-2	RP-LC-HRMS/MS	ESI+	6.0	$C_{24}H_{48}NO_7P$	2	Glycerophospholipids	Glycerophosphocholines
82	LPC 18:0 sn-1	RP-LC-HRMS/MS	ESI+	9.3	$C_{26}H_{54}NO_7P$	2	Glycerophospholipids	Glycerophosphocholines
83	LPC 18:0 sn-2	RP-LC-HRMS/MS	ESI+	8.9	$C_{26}H_{54}NO_7P$	2	Glycerophospholipids	Glycerophosphocholines
84	LPC 18:1 sn-1	RP-LC-HRMS/MS	ESI+	7.9	$C_{26}H_{52}NO_7P$	2	Glycerophospholipids	Glycerophosphocholines
85	LPC 18:1 sn-2	RP-LC-	ESI+	7.6	$C_{26}H_{52}NO_7P$	2	Glycerophospholipids	Glycerophosphocholines

		HRMS/MS						
86	LPC 18:2 sn-1	RP-LC-HRMS/MS	ESI+	6.8	$C_{26}H_{50}NO_7P$	2	Glycerophospholipids	Glycerophosphocholines
87	LPC 18:2 sn-2	RP-LC-HRMS/MS	ESI+	6.6	$C_{26}H_{50}NO_7P$	2	Glycerophospholipids	Glycerophosphocholines
88	LPC 20:0 sn-1	RP-LC-HRMS/MS	ESI+	11.4	$C_{28}H_{58}NO_7P$	2	Glycerophospholipids	Glycerophosphocholines
89	LPC 20:0 sn-2	RP-LC-HRMS/MS	ESI+	11.0	$C_{28}H_{58}NO_7P$	2	Glycerophospholipids	Glycerophosphocholines
90	LPC 20:1 sn-1	RP-LC-HRMS/MS	ESI+	9.6	$C_{28}H_{56}NO_7P$	2	Glycerophospholipids	Glycerophosphocholines
91	LPC 20:1 sn-2	RP-LC-HRMS/MS	ESI+	9.3	$C_{28}H_{56}NO_7P$	2	Glycerophospholipids	Glycerophosphocholines
92	LPC 20:4 sn-1	RP-LC-HRMS/MS	ESI+	6.8	$C_{28}H_{50}NO_7P$	2	Glycerophospholipids	Glycerophosphocholines
93	LPC 20:4 sn-2	RP-LC-HRMS/MS	ESI+	6.6	$C_{28}H_{50}NO_7P$	2	Glycerophospholipids	Glycerophosphocholines
94	LPC 20:5 sn-1	RP-LC-HRMS/MS	ESI+	6.1	$C_{28}H_{48}NO_7P$	2	Glycerophospholipids	Glycerophosphocholines
95	LPC 20:5 sn-2	RP-LC-HRMS/MS	ESI+	5.8	$C_{28}H_{48}NO_7P$	2	Glycerophospholipids	Glycerophosphocholines
96	LPC 22:4 sn-1	RP-LC-HRMS/MS	ESI+	8.1	$C_{30}H_{54}NO_7P$	2	Glycerophospholipids	Glycerophosphocholines
97	LPC 22:4 sn-2	RP-LC-HRMS/MS	ESI+	7.8	$C_{30}H_{54}NO_7P$	2	Glycerophospholipids	Glycerophosphocholines
98	LPC 22:6 sn-1	RP-LC-HRMS/MS	ESI+	6.8	$C_{30}H_{50}NO_7P$	2	Glycerophospholipids	Glycerophosphocholines
99	LPC 22:6 sn-2	RP-LC-HRMS/MS	ESI+	6.6	$C_{30}H_{50}NO_7P$	2	Glycerophospholipids	Glycerophosphocholines
100	LPE 16:0 sn-1	RP-LC-HRMS/MS	ESI+	7.4	$C_{21}H_{44}NO_7P$	2	Glycerophospholipids	Glycerophosphoethanolamines
101	LPE 16:0 sn-2	RP-LC-HRMS/MS	ESI+	7.1	$C_{21}H_{44}NO_7P$	2	Glycerophospholipids	Glycerophosphoethanolamines
102	LPE 16:1 sn-1	RP-LC-HRMS/MS	ESI+	6.3	$C_{21}H_{42}NO_7P$	2	Glycerophospholipids	Glycerophosphoethanolamines
103	LPE 16:1 sn-2	RP-LC-HRMS/MS	ESI+	6.0	$C_{21}H_{42}NO_7P$	2	Glycerophospholipids	Glycerophosphoethanolamines
104	LPE 18:0 sn-1	RP-LC-HRMS/MS	ESI+	9.3	$C_{23}H_{48}NO_7P$	2	Glycerophospholipids	Glycerophosphoethanolamines
105	LPE 18:0 sn-2	RP-LC-HRMS/MS	ESI+	8.9	$C_{23}H_{48}NO_7P$	2	Glycerophospholipids	Glycerophosphoethanolamines
106	LPE 18:1 sn-1	RP-LC-HRMS/MS	ESI+	7.8	$C2_3H_{46}NO_7P$	2	Glycerophospholipids	Glycerophosphoethanolamines
107	LPE 18:1 sn-2	RP-LC-HRMS/MS	ESI+	7.5	$C_{23}H_{46}NO_7P$	2	Glycerophospholipids	Glycerophosphoethanolamines

108	LPE 18:2 sn-1	RP-LC-HRMS/MS	ESI+	6.8	$C_{23}H_{44}NO_7P$	2	Glycerophospholipids	Glycerophosphoethanolamines
109	LPE 18:2 sn-2	RP-LC-HRMS/MS	ESI+	6.5	$C_{23}H_{44}NO_7P$	2	Glycerophospholipids	Glycerophosphoethanolamines
110	LPE 20:1 sn-1	RP-LC-HRMS/MS	ESI+	9.6	$C_{25}H_{50}NO_7P$	2	Glycerophospholipids	Glycerophosphoethanolamines
111	LPE 20:1 sn-2	RP-LC-HRMS/MS	ESI+	9.2	$C_{25}H_{50}NO_7P$	2	Glycerophospholipids	Glycerophosphoethanolamines
112	LPE 20:2 sn-1	RP-LC-HRMS/MS	ESI+	8.2	$C_{25}H_{48}NO_7P$	2	Glycerophospholipids	Glycerophosphoethanolamines
113	LPE 20:2 sn-2	RP-LC-HRMS/MS	ESI+	8.0	$C_{25}H_{48}NO_7P$	2	Glycerophospholipids	Glycerophosphoethanolamines
114	LPE 20:4 sn-1	RP-LC-HRMS/MS	ESI+	6.8	$C_{25}H_{44}NO_7P$	2	Glycerophospholipids	Glycerophosphoethanolamines
115	LPE 20:4 sn-2	RP-LC-HRMS/MS	ESI+	6.6	$C_{25}H_{44}NO_7P$	2	Glycerophospholipids	Glycerophosphoethanolamines
116	LPE 22:4 sn-1	RP-LC-HRMS/MS	ESI+	8.0	$C_{27}H_{48}NO_7P$	2	Glycerophospholipids	Glycerophosphoethanolamines
117	LPE 22:4 sn-2	RP-LC-HRMS/MS	ESI+	7.8	$C_{27}H_{48}NO_7P$	2	Glycerophospholipids	Glycerophosphoethanolamines
118	LPE 22:6 sn-1	RP-LC-HRMS/MS	ESI+	6.7	$C_{27}H_{44}NO_7P$	2	Glycerophospholipids	Glycerophosphoethanolamines
119	LPE 22:6 sn-2	RP-LC-HRMS/MS	ESI+	6.5	$C_{27}H_{44}NO_7P$	2	Glycerophospholipids	Glycerophosphoethanolamines
120	LPE P-16:0	RP-LC-HRMS/MS	ESI+	7.9	$C_{21}H_{44}NO_6P$	2	Glycerophospholipids	Glycerophosphoethanolamines
121	LPE P-18:0	RP-LC-HRMS/MS	ESI+	9.8	$C_{23}H_{48}NO_6P$	2	Glycerophospholipids	Glycerophosphoethanolamines
122	LPE P-18:1	RP-LC-HRMS/MS	ESI+	8.3	$C2_3H_{46}NO_6P$	2	Glycerophospholipids	Glycerophosphoethanolamines
123	LPE P-20:0	RP-LC-HRMS/MS	ESI+	12.0	$C_{25}H_{52}NO_6P$	2	Glycerophospholipids	Glycerophosphoethanolamines
124	LPE P-20:1	RP-LC-HRMS/MS	ESI+	10.1	$C_{25}H_{50}NO_6P$	2	Glycerophospholipids	Glycerophosphoethanolamines
125	LPE P-22:1	RP-LC-HRMS/MS	ESI+	12.3	$C_{27}H_{54}NO_6P$	2	Glycerophospholipids	Glycerophosphoethanolamines
126	LPG 16:0 sn-1	RP-LC-HRMS/MS	ESI-	9.0	$C_{22}H_{45}O_9P$	2	Glycerophospholipids	Glycerophosphoglycerols
127	LPG 16:0 sn-2	RP-LC-HRMS/MS	ESI-	8.5	$C_{24}H_{50}NO_7P$	2	Glycerophospholipids	Glycerophosphoglycerols
128	LPG 18:0 sn-1	RP-LC-HRMS/MS	ESI-	12.1	$C_{24}H_{49}O_9P$	2	Glycerophospholipids	Glycerophosphoglycerols
129	LPG 18:0 sn-2	RP-LC-HRMS/MS	ESI-	11.3	$C_{24}H_{49}O_9P$	2	Glycerophospholipids	Glycerophosphoglycerols
130	LPG 18:1 sn-1	RP-LC-	ESI-	9.6	$C_{24}H_{47}O_9P$	2	Glycerophospholipids	Glycerophosphoglycerols

		HRMS/MS						
131	LPG 18:1 sn-2	RP-LC-HRMS/MS	ESI-	9.1	$C_{24}H_{47}O_9P$	2	Glycerophospholipids	Glycerophosphoglycerols
132	LPG 20:4 sn-1	RP-LC-HRMS/MS	ESI-	8.1	$C_{26}H_{45}O_9P$	2	Glycerophospholipids	Glycerophosphoglycerols
133	LPG 20:4 sn-2	RP-LC-HRMS/MS	ESI-	7.8	$C_{26}H_{45}O_9P$	2	Glycerophospholipids	Glycerophosphoglycerols
134	LPG 22:6 sn-1	RP-LC-HRMS/MS	ESI-	8.0	$C28H_{45}O_9P$	2	Glycerophospholipids	Glycerophosphoglycerols
135	LPG 22:6 sn-2	RP-LC-HRMS/MS	ESI-	7.8	$C_{28}H_{45}O_9P$	2	Glycerophospholipids	Glycerophosphoglycerols
136	LPI 16:0 sn-1	RP-LC-HRMS/MS	ESI-	8.0	$C_{25}H_{49}O_{12}P$	2	Glycerophospholipids	Glycerophosphoinositols
137	LPI 16:0 sn-2	RP-LC-HRMS/MS	ESI-	7.5	$C_{25}H_{49}O_{12}P$	2	Glycerophospholipids	Glycerophosphoinositols
138	LPI 18:0 sn-1	RP-LC-HRMS/MS	ESI-	10.5	$C_{27}H_{53}O_{12}P$	2	Glycerophospholipids	Glycerophosphoinositols
139	LPI 18:0 sn-2	RP-LC-HRMS/MS	ESI-	9.9	$C_{27}H_{53}O_{12}P$	2	Glycerophospholipids	Glycerophosphoinositols
140	LPI 18:1 sn-1	RP-LC-HRMS/MS	ESI-	8.5	$C_{27}H_{51}O_{12}P$	2	Glycerophospholipids	Glycerophosphoinositols
141	LPI 18:1 sn-2	RP-LC-HRMS/MS	ESI-	8.1	$C_{27}H_{51}O_{12}P$	2	Glycerophospholipids	Glycerophosphoinositols
142	LPI 20:4 sn-1	RP-LC-HRMS/MS	ESI-	7.3	$C_{29}H_{49}O_{12}P$	2	Glycerophospholipids	Glycerophosphoinositols
143	LPI 20:4 sn-2	RP-LC-HRMS/MS	ESI-	7.0	$C_{29}H_{49}O_{12}P$	2	Glycerophospholipids	Glycerophosphoinositols
144	LPI 22:6 sn-1	RP-LC-HRMS/MS	ESI-	7.2	$C_{31}H_{49}O_{12}P$	2	Glycerophospholipids	Glycerophosphoinositols
145	LPI 22:6 sn-2	RP-LC-HRMS/MS	ESI-	7.0	$C_{31}H_{49}O_{12}P$	2	Glycerophospholipids	Glycerophosphoinositols
146	LPS 18:0 sn-1	RP-LC-HRMS/MS	ESI-	10.0	$C_{24}H_{48}NO_9P$	2	Glycerophospholipids	Glycerophosphoserines
147	LPS 18:0 sn-2	RP-LC-HRMS/MS	ESI-	9.5	$C_{24}H_{48}NO_9P$	2	Glycerophospholipids	Glycerophosphoserines
148	LPS 18:1 sn-1	RP-LC-HRMS/MS	ESI-	8.4	$C_{24}H_{46}NO_9P$	2	Glycerophospholipids	Glycerophosphoserines
149	LPS 18:1 sn-2	RP-LC-HRMS/MS	ESI-	7.9	$C_{24}H_{46}NO_9P$	2	Glycerophospholipids	Glycerophosphoserines
150	Lysine	GC-MS	EI+	21.8	$C_6H_{14}N_2O_2$	2	Carboxylic acids and derivatives	Amino acids, peptides, and analogues
151	Malic acid	GC-MS/MS	EI+	18.1	$C_4H_6O_5$	1	Hydroxy acids and derivatives	Beta hydroxy acids and derivatives
152	Malonic acid	GC-MS/MS	EI+	10.6	$C_3H_4O_4$	1	Carboxylic acids and derivatives	Dicarboxylic acids and derivatives

153	Methionine	HILIC-MS/MS	ESI+	14.0	$C_5H_{11}NO_2S$	1	Carboxylic acids and derivatives	Carboxylic acids and derivatives
154	Methylamine	HILIC-MS/MS	ESI+	10.0	CH_5N	1	Organonitrogen compounds	Amines
155	MG 14:0 (1)	GC-MS	EI+	27.3	$C_{17}H_{34}O_4$	2	Glycerolipids	Monoacylglycerols
156	MG 16:0 (1)	RP-LC-HRMS/MS	ESI+	10.8	$C_{19}H_{38}O_4$	2	Glycerolipids	Monoacylglycerols
157	MG 16:0 (2)	RP-LC-HRMS/MS	ESI+	11.2	$C_{19}H_{38}O_4$	2	Glycerolipids	Monoacylglycerols
158	MG 18:0 (1)	RP-LC-HRMS/MS	ESI+	13.2	$C_{21}H_{42}O_4$	2	Glycerolipids	Monoacylglycerols
159	MG 18:0 (2)	RP-LC-HRMS/MS	EI+	29.5	$C_{21}H_{42}O_4$	2	Glycerolipids	Monoacylglycerols
160	MG 18:1 (1)	RP-LC-HRMS/MS	EI+	29.4	$C_{21}H_{40}O_4$	2	Glycerolipids	Monoacylglycerols
161	MG 18:1 (2)	RP-LC-HRMS/MS	ESI+	11.6	$C_{21}H_{40}O_4$	2	Glycerolipids	Monoacylglycerols
162	MG 18:2 (2)	GC-MS	EI+	29.9	$C_{21}H_{36}O_4$	2	Glycerolipids	Monoacylglycerols
163	MG 20:4 (1)	RP-LC-HRMS/MS	ESI+	9.6	$C_{23}H_{38}O_4$	2	Glycerolipids	Monoacylglycerols
164	MG 20:4 (2)	RP-LC-HRMS/MS	ESI+	9.8	$C_{23}H_{38}O_4$	2	Glycerolipids	Monoacylglycerols
165	MG 22:6 (1)	RP-LC-HRMS/MS	ESI+	9.4	$C_{25}H_{38}O_4$	2	Glycerolipids	Monoacylglycerols
166	MG 22:6 (2)	RP-LC-HRMS/MS	ESI+	9.6	$C_{25}H_{38}O_4$	2	Glycerolipids	Monoacylglycerols
167	Monoisoamylamine	HILIC-MS/MS	ESI+	4.6	$C_5H_{13}N$	1	Organonitrogen compounds	Amines
168	Myo-Inositol	GC-MS	EI+	25.2	$C_6H_{12}O_6$	2	Organooxygen compounds	Alcohols and polyols
169	NAA (N-acetylaspartate)	RP-LC-HRMS/MS	ESI+	0.8	$C_6H_9NO_5$	2	Carboxylic acids and derivatives	Amino acids, peptides, and analogues
170	NAAG (N-acetyl-aspartyl-glutamate)	RP-LC-HRMS/MS	ESI+	1.1	$C_{11}H_{16}N_2O_8$	2	Carboxylic acids and derivatives	Amino acids, peptides, and analogues
171	N-Acetyl-L-glutamic acid	GC-MS	EI+	17.2	$C_7H_{11}NO_5$	2	Carboxylic acids and derivatives	Amino acids, peptides, and analogues
172	N-Acetylneuraminic acid	RP-LC-HRMS/MS	ESI-	0.6	$C_{11}H_{19}NO_9$	1	Organooxygen compounds	Carbohydrates and carbohydrate conjugates
173	Nervonic acid	GC-MS	EI+	29.6	$C_{24}H_{46}O_2$	2	Fatty Acyls	Fatty acids and conjugates
174	Nicotinamide	RP-LC-HRMS/MS	ESI+	0.9	$C_6H_6N_2O$	1	Pyridines and derivatives	Pyridinecarboxylic acids and derivatives
175	Nicotinic acid	HILIC-MS/MS	ESI+	10.5	$C_6H_5NO_2$	1	Pyridines and derivatives	Pyridinecarboxylic acids and derivatives
176	N-oleoyl taurine	RP-LC-HRMS/MS	ESI-	9.6	$C_{20}H_{39}NO_4S$	2	Fatty Acyls	Fatty amides
177	Nonanoic acid	GC-MS	EI+	15.6	$C_9H_{18}O_2$	2	Fatty Acyls	Fatty acids and conjugates

178	Octadecadienoic acid	GC-MS	EI+	25.9	$C_{18}H_{32}O_2$	2	Fatty Acyls	Lineolic acids and derivatives
179	Oleic acid	RP-LC-HRMS/MS	ESI+	12.9	$C_{18}H_{34}O_2$	2	Fatty Acyls	Fatty acids and conjugates
180	Orthophosphate	RP-LC-HRMS/MS	ESI-	0.6	H_3O_4P	1	Non-metal oxoanionic compounds	Non-metal phosphates
181	Palmitic acid	RP-LC-HRMS/MS	ESI+	10.5	$C_{16}H_{32}O_2$	2	Fatty Acyls	Fatty acids and conjugates
182	Palmitoylcarnitine	RP-LC-HRMS/MS	ESI+	7.3	$C_{23}H_{46}NO_4$	2	Fatty Acyls	Fatty acid esters
183	Pantothenate	RP-LC-HRMS/MS	ESI+	2.5	$C_9H_{17}NO_5$	1	Organooxygen compounds	Alcohols and polyols
184	Phenylalanine	HILIC-MS/MS	ESI+	12.6	$C_9H_{11}NO_2$	1	Carboxylic acids and derivatives	Amino acids, peptides, and analogues
185	Phosphocholine	RP-LC-HRMS/MS	ESI+	0.6	$C_5H_{14}NO_4P$	1	Organic phosphoric acids and derivatives	Phosphate esters
186	Phosphorylethanolamine	RP-LC-HRMS/MS	ESI-	0.6	$C_2H_8NO_4P$	1	Organic phosphoric acids and derivatives	Phosphate esters
187	Pimelic acid	GC-MS/MS	EI+	20.5	$C_7H_{12}O_4$	1	Fatty Acyls	Fatty acids and conjugates
188	Pipecolic acid	RP-LC-HRMS/MS	ESI+	0.5	$C_6H_{11}NO_2$	2	Carboxylic acids and derivatives	Amino acids, peptides, and analogues
189	Proline	RP-LC-HRMS/MS	ESI+	0.6	$C_5H_9NO_2$	1	Carboxylic acids and derivatives	Amino acids, peptides, and analogues
190	Putrescine	HILIC-MS/MS	ESI+	21.0	$C_4H_{12}N_2$	1	Organonitrogen compounds	Amines
191	Pyridoxine	HILIC-MS/MS	ESI+	2.0	$C_8H_{11}NO_3$	1	Pyridines and derivatives	Pyridoxines
192	Pyroglutamic acid	HILIC-MS/MS	ESI+	15.0	$C_5H_7NO_3$	1	Carboxylic acids and derivatives	Amino acids, peptides, and analogues
193	Pyruvic acid	GC-MS/MS	EI+	6.6	$C_3H_4O_3$	1	Keto acids and derivatives	Alpha-keto acids and derivatives
194	Riboflavine	HILIC-MS/MS	ESI+	9.6	$C_{17}H_{20}N_4O_6$	1	Pteridines and derivatives	Alloxazines and isoalloxazines
195	Ribose	HILIC-MS/MS	ESI-	4.2	$C_5H_{10}O_5$	1	Organooxygen compounds	Carbohydrates and carbohydrate conjugates
196	S-Adenosyl-L-homocysteine	RP-LC-HRMS/MS	ESI+	1.2	$C_{14}H_{20}N_6O_5S$	1	Lactones	Gamma butyrolactones
197	Serine	HILIC-MS/MS	ESI+	17.9	$C_3H_7NO_3$	1	Carboxylic acids and derivatives	Amino acids, peptides, and analogues
198	Stearic acid	GC-MS	EI+	26.2	$C_{18}H_{36}O_2$	2	Fatty Acyls	Fatty acids and conjugates
199	Succinic acid	RP-LC-HRMS/MS	ESI+	1.0	$C_4H_6O_4$	1	Carboxylic acids and derivatives	Dicarboxylic acids and derivatives
200	Tartaric acid	GC-MS/MS	EI+	21.8	$C_4H_6O_6$	1	Organooxygen compounds	Carbohydrates and carbohydrate conjugates
201	Taurine	HILIC-MS/MS	ESI+	14.4	$C_2H_7NO_3S$	1	Organic sulfonic acids and derivatives	Organosulfonic acids and derivatives
202	Taurocholic acid	RP-LC-	ESI-	3.4	$C_{26}H_{45}NO_7S$	1	Steroids and steroid derivatives	Bile acids, alcohols and derivatives

		HRMS/MS						
203	Thiamine	HILIC-MS/MS	ESI+	11.8	$C_{12}H_{17}N_4OS$	1	Diazines	Pyrimidines and pyrimidine derivatives
204	Threonine	HILIC-MS/MS	ESI+	10.0	$C_4H_9NO_3$	1	Carboxylic acids and derivatives	Amino acids, peptides, and analogues
205	Thymidine	HILIC-MS/MS	ESI+	1.6	$C_{10}H_{14}N_2O_5$	1	Pyrimidine nucleosides	Pyrimidine 2'-deoxyribonucleosides
206	Thymine	HILIC-MS/MS	ESI+	1.2	$C_5H_6N_2O_2$	1	Diazines	Pyrimidines and pyrimidine derivatives
207	Trimethylamine	HILIC-MS/MS	ESI+	5.6	C_3H_9N	1	Organonitrogen compounds	Amines
208	Trimethylamine-n-oxide	HILIC-MS/MS	ESI+	13.0	C_3H_9NO	1	Organonitrogen compounds	Aminoxides
209	Trimethyllysine (N6,N6,N6)	RP-LC-HRMS/MS	ESI+	0.5	$C_9H_{20}N_2O_2$	2	Indoles and derivatives	Tryptamines and derivatives
210	Tryptamine	HILIC-MS/MS	ESI+	4.1	$C_{10}H_{12}N_2$	1	Indoles and derivatives	Tryptamines and derivatives
211	Tryptophan	HILIC-MS/MS	ESI+	12.7	$C_{11}H_{12}N_2O_2$	1	Indoles and derivatives	Indolyl carboxylic acids and derivatives
212	Tyrosine	HILIC-MS/MS	ESI+	14.5	$C_9H_{11}NO_3$	1	Carboxylic acids and derivatives	Amino acids, peptides, and analogues
213	Uracil	GC-MS	EI+	15.4	$C_4H_4N_2O_2$	2	Diazines	Pyrimidines and pyrimidine derivatives
214	Uridine	RP-LC-HRMS/MS	ESI+	1.1	$C_9H_{12}N_2O_6$	1	Pyrimidine nucleosides	
215	Valine	GC-MS	EI+	13.0	$C_5H_{11}NO_2$	2	Carboxylic acids and derivatives	Amino acids, peptides, and analogues
216	Xanthine	HILIC-MS/MS	ESI+	7.4	$C_5H_4N_4O_2$	1	Imidazopyrimidines	Purines and purine derivatives
217	γ-Aminobutyric acid	GC-MS	EI+	18.1	$C_4H_9NO_2$	2	Carboxylic acids and derivatives	Amino acids, peptides, and analogues

Table S2. Calculated *p*-values and Log2FC values from pairwise comparisons of each treatment group G1: metronidazole; G2: probiotics; G3: fecal microbiota transplantation with either the *C. difficile* infected and untreated group G4 or the uninfected and untreated control group G5.Values highlighted in red correspond to $p \leq 0.05$ or $|Log2FC| \geq 1$.

**Comparisons**	**1v4**		**1v5**		**2v4**		**2v5**		**3v4**		**3v5**		**4v5**		
**Compounds**	***p* value**	**Log2FC**	***p* value**	**Log2FC**	***p* value**	**Log2FC**	***p* value**	**Log2FC**	***p* value**	**Log2FC**	***p* value**	**Log2FC**	***p* value**	**Log2FC**	**CV%**
1,5-Anhydroglucitol	2.52E-01	1.88E-01	6.39E-01	-6.76E-02	8.40E-02	2.72E-01	9.02E-01	1.66E-02	1.36E-01	2.16E-01	7.50E-01	-3.96E-02	7.40E-02	-2.55E-01	6.12E+00
2,3-Butanediol	2.23E-02	1.03E-01	1.78E-02	1.03E-01	6.25E-01	3.21E-02	6.18E-01	3.22E-02	5.33E-01	3.06E-02	5.19E-01	3.07E-02	9.99E-01	7.25E-05	1.73E+01
2-Hydroxybutyric acid	7.50E-02	-3.60E-01	1.87E-03	-5.61E-01	1.81E-01	-2.63E-01	7.54E-03	-4.63E-01	2.22E-01	3.75E-01	5.25E-01	1.74E-01	2.99E-01	-2.01E-01	1.92E+01
2-Ketoglutaric acid	1.59E-01	2.36E-01	6.10E-01	1.11E-01	2.62E-01	2.76E-01	5.86E-01	1.50E-01	4.08E-02	5.56E-01	1.35E-01	4.31E-01	5.92E-01	-1.26E-01	2.35E+01
2-Hydroxyisovaleric acid	1.51E-01	4.22E-01	6.91E-02	-5.66E-01	5.69E-02	6.19E-01	2.29E-01	-3.68E-01	9.90E-01	-2.87E-03	2.95E-03	-9.90E-01	3.86E-03	-9.88E-01	1.19E+01

3,4-Dihydroxyphenylacetic acid	5.81E-01	-6.81E-02	5.11E-04	-4.09E-01	4.61E-02	2.65E-01	4.69E-01	-7.53E-02	6.52E-02	3.27E-01	9.28E-01	-1.30E-02	8.29E-03	-3.41E-01	1.33E+01
3-Hydroxy-3-methylglutaric acid	3.70E-02	-2.42E-01	5.93E-02	-3.21E-01	6.65E-01	5.63E-02	8.93E-01	-2.26E-02	4.47E-01	9.74E-02	9.10E-01	1.85E-02	6.12E-01	-7.89E-02	2.04E+01
3-Hydroxybutyric acid	4.55E-04	-6.97E-01	5.69E-06	-5.87E-01	2.22E-02	-3.91E-01	1.13E-02	-2.81E-01	7.41E-01	-6.76E-02	8.09E-01	4.29E-02	4.51E-01	1.10E-01	1.97E+01
3-Methyl-2-oxovaleric acid	6.29E-03	-4.67E-01	1.37E-02	-4.39E-01	3.70E-01	-1.41E-01	4.90E-01	-1.14E-01	5.82E-01	-9.27E-02	7.10E-01	-6.52E-02	8.80E-01	2.75E-02	1.98E+01
4-Hydroxybenzoic acid	4.13E-04	4.89E-01	5.33E-02	2.61E-01	4.89E-03	5.56E-01	7.68E-02	3.28E-01	5.93E-03	2.32E-01	9.67E-01	4.27E-03	4.85E-02	-2.28E-01	3.07E+01
4-Hydroxyphenyllactic acid	1.08E-02	3.75E-01	1.99E-02	3.05E-01	1.35E-01	4.46E-01	1.87E-01	3.76E-01	3.04E-01	5.69E-01	3.53E-01	4.99E-01	6.22E-01	-6.99E-02	1.05E+01
5-Hydroxyindole-3-acetic acid	4.66E-02	-2.32E-01	3.91E-04	-3.87E-01	3.08E-01	1.54E-01	9.98E-01	-3.11E-04	1.00E-02	3.10E-01	1.10E-01	1.56E-01	1.90E-01	-1.54E-01	1.89E+01
5-Methylthioadenosine	3.91E-03	2.75E-01	3.31E-07	7.58E-01	1.78E-01	1.25E-01	1.55E-05	6.07E-01	1.30E-01	2.47E-01	8.31E-04	7.29E-01	1.90E-04	4.82E-01	5.61E+00
Acetylcarnitine	1.83E-06	-4.11E-01	2.97E-02	-1.25E-01	2.58E-04	-2.97E-01	8.55E-01	-1.15E-02	9.12E-02	-1.35E-01	6.32E-02	1.50E-01	3.74E-04	2.86E-01	5.86E+00
Aconitic acid	2.85E-01	1.48E-01	7.91E-01	-4.41E-02	3.75E-02	4.30E-01	2.58E-01	2.38E-01	1.89E-01	1.76E-01	9.20E-01	-1.62E-02	2.83E-01	-1.92E-01	6.83E+00
Adenosine	3.82E-02	-6.62E-01	8.22E-01	-8.03E-02	8.93E-01	3.00E-02	2.75E-02	6.11E-01	4.05E-01	-2.55E-01	3.71E-01	3.26E-01	4.55E-02	5.81E-01	3.88E+00
Adenosine monophosphate	2.57E-01	7.23E-02	1.80E-02	2.30E-01	8.27E-01	2.21E-02	1.56E-01	1.80E-01	9.80E-01	3.18E-03	2.85E-01	1.61E-01	7.85E-02	1.58E-01	1.85E+00
Adrenic acid	1.88E-01	1.30E-01	3.12E-01	-1.18E-01	4.52E-01	4.60E-02	4.29E-02	-2.03E-01	2.73E-01	9.33E-02	1.59E-01	-1.55E-01	2.62E-02	-2.49E-01	1.60E+00
Alanine	2.60E-02	8.57E-01	4.52E-03	-2.30E+00	1.96E-02	1.24E+00	8.67E-03	-1.93E+00	2.30E-02	1.69E+00	2.55E-02	-1.47E+00	1.89E-03	-3.16E+00	2.77E+00
alpha-D-Lyxopyranose	1.86E-02	-1.90E-01	7.38E-03	-2.62E-01	6.82E-01	6.43E-02	9.64E-01	-7.17E-03	4.82E-01	9.78E-02	8.53E-01	2.63E-02	2.72E-01	-7.15E-02	1.39E+01
alpha-Linolenic acid	1.75E-01	1.06E-01	8.52E-01	-1.24E-02	2.88E-02	2.41E-01	2.04E-01	1.23E-01	2.63E-01	1.04E-01	8.67E-01	-1.39E-02	6.09E-02	-1.18E-01	2.49E+00
alpha-Tocopherol	1.86E-01	-1.05E-01	4.53E-01	-5.62E-02	3.84E-01	1.02E-01	2.03E-01	1.50E-01	2.65E-01	1.58E-01	1.52E-01	2.07E-01	5.29E-01	4.85E-02	6.78E+00
Arabinitol	8.73E-01	1.22E-02	6.98E-01	-2.91E-02	4.07E-01	9.98E-02	6.19E-01	5.85E-02	3.59E-01	8.59E-02	6.26E-01	4.46E-02	4.76E-01	-4.13E-02	4.17E+00
Arachidonic acid	3.34E-03	-1.86E-01	5.48E-04	-2.55E-01	5.45E-01	-4.92E-02	1.69E-01	-1.18E-01	5.03E-01	5.55E-02	8.73E-01	-1.35E-02	2.80E-01	-6.90E-02	1.07E+00
Arachidonoylcarnitine	9.19E-05	3.49E-01	1.50E-01	1.17E-01	7.64E-03	2.47E-01	8.72E-01	1.44E-02	3.36E-02	2.70E-01	7.52E-01	3.77E-02	2.39E-02	-2.33E-01	4.05E+00
Arginine	1.16E-01	-1.33E-01	4.17E-05	-3.13E-01	3.61E-02	-1.90E-01	1.08E-05	-3.70E-01	2.02E-01	-9.36E-02	1.36E-05	-2.74E-01	9.88E-03	-1.80E-01	3.64E+00
Asparagine	1.69E-02	9.06E-01	2.86E-02	-3.25E+00	2.72E-02	1.48E+00	3.87E-02	-2.67E+00	4.15E-02	2.38E+00	8.70E-02	-1.77E+00	2.18E-02	-4.15E+00	1.40E+01
Aspartic acid	2.94E-02	-8.60E-02	2.18E-05	-1.89E-01	1.49E-01	-6.86E-02	7.73E-04	-1.71E-01	8.39E-02	-9.20E-02	6.35E-04	-1.95E-01	1.97E-02	-1.03E-01	3.82E+00
Azelaic acid	8.71E-01	2.27E-02	1.50E-01	1.80E-01	4.74E-02	3.07E-01	2.55E-03	4.64E-01	5.02E-02	2.43E-01	5.54E-04	4.01E-01	2.78E-01	1.57E-01	1.11E+01
Benzoic acid	1.23E-01	-2.02E-01	1.04E-02	-2.41E-01	9.18E-01	1.43E-02	8.18E-01	-2.50E-02	6.12E-01	9.28E-02	7.39E-01	5.34E-02	7.62E-01	-3.94E-02	9.86E+00
beta-Arabinopyranose	7.59E-02	3.12E-01	9.07E-01	2.89E-02	2.00E-01	4.65E-01	6.25E-01	1.82E-01	7.78E-01	5.06E-02	3.90E-01	-2.33E-01	2.29E-01	-2.83E-01	4.77E+00
Betaine	8.41E-01	3.03E-02	1.61E-03	-7.14E-01	3.56E-01	-1.32E-01	2.79E-04	-8.76E-01	2.96E-01	2.65E-01	5.96E-02	-4.79E-01	1.42E-03	-7.44E-01	7.26E+00
Carnitine	4.22E-06	1.43E-01	3.88E-05	1.18E-01	2.84E-01	3.61E-02	7.35E-01	1.11E-02	1.67E-01	6.64E-02	3.77E-01	4.14E-02	2.93E-01	-2.50E-02	2.92E+00
Cholesterol	1.10E-01	9.04E-02	2.36E-02	1.37E-01	1.80E-01	7.58E-02	4.24E-02	1.22E-01	9.21E-01	7.16E-03	4.73E-01	5.36E-02	3.52E-01	4.64E-02	4.23E+00
Choline	5.07E-01	6.65E-02	3.69E-02	-3.02E-01	2.29E-01	1.36E-01	1.10E-01	-2.33E-01	5.18E-01	1.40E-01	2.94E-01	-2.29E-01	1.74E-02	-3.69E-01	2.20E+00
Citramalic acid	9.29E-01	1.15E-02	3.92E-02	-3.59E-01	1.31E-02	3.66E-01	9.75E-01	-4.96E-03	1.26E-01	2.38E-01	4.43E-01	-1.33E-01	2.81E-02	-3.71E-01	1.89E+01
Citric acid	7.48E-01	5.04E-02	1.01E-02	-3.88E-01	1.96E-02	3.80E-01	6.58E-01	-5.84E-02	1.48E-01	2.28E-01	1.29E-01	-2.10E-01	4.16E-03	-4.38E-01	6.09E+00
Citrulline	2.21E-01	-1.67E-01	9.64E-03	-3.60E-01	1.14E-02	-3.69E-01	2.43E-04	-5.61E-01	3.75E-01	-1.17E-01	1.97E-02	-3.09E-01	1.66E-01	-1.92E-01	8.49E+00
Creatine	1.77E-04	1.71E+00	1.54E-02	-1.32E+00	5.26E-04	1.80E+00	2.13E-02	-1.23E+00	8.54E-03	2.14E+00	9.15E-02	-8.88E-01	7.91E-04	-3.02E+00	2.46E+00

Creatinine	5.08E-01	1.26E-01	5.93E-03	-8.13E-01	2.72E-01	2.45E-01	1.76E-02	-6.94E-01	1.60E-01	4.50E-01	1.20E-01	-4.89E-01	3.70E-03	-9.40E-01	4.78E+00
Cysteine	9.78E-01	-2.53E-03	6.51E-01	4.37E-02	1.51E-01	2.17E-01	9.44E-02	2.63E-01	6.22E-01	4.61E-02	3.54E-01	9.23E-02	5.42E-01	4.62E-02	1.37E+01
Cytidine	2.34E-01	1.60E-01	2.58E-04	5.93E-01	8.34E-01	2.78E-02	2.22E-03	4.60E-01	3.81E-01	1.13E-01	3.44E-04	5.45E-01	1.22E-02	4.32E-01	4.66E+00
Cytidine monophosphate	9.03E-01	1.19E-02	6.12E-02	-1.83E-01	6.41E-01	-3.72E-02	6.80E-03	-2.32E-01	6.65E-01	4.26E-02	1.17E-01	-1.53E-01	4.98E-02	-1.95E-01	5.07E+00
Cytosine	3.19E-01	1.25E-01	6.44E-04	5.33E-01	8.75E-01	1.93E-02	3.60E-03	4.27E-01	4.36E-01	8.95E-02	5.05E-04	4.98E-01	8.59E-03	4.08E-01	9.12E+00
D-Erythrose	3.56E-03	-3.02E-01	9.15E-01	2.03E-02	8.76E-03	-1.87E-01	4.34E-01	1.35E-01	2.35E-03	-1.80E-01	3.92E-01	1.43E-01	3.93E-02	3.22E-01	8.47E+00
D-Fructose	3.70E-03	4.96E-01	4.15E-01	1.32E-01	7.78E-02	2.92E-01	6.70E-01	-7.23E-02	3.21E-01	1.62E-01	2.53E-01	-2.02E-01	4.87E-02	-3.64E-01	3.86E+00
D-Fucitol	2.94E-01	-4.90E-02	1.25E-02	-2.33E-01	5.38E-01	3.34E-02	9.79E-02	-1.51E-01	4.45E-01	3.01E-02	6.80E-02	-1.54E-01	3.05E-02	-1.84E-01	8.62E+00
D-Glucose	9.50E-01	-6.29E-03	3.47E-01	-1.11E-01	6.17E-01	9.84E-02	9.76E-01	-6.11E-03	6.74E-01	7.37E-02	8.63E-01	-3.09E-02	2.93E-01	-1.05E-01	1.17E+01
Dimethylamine	1.33E-03	-4.93E-01	8.02E-05	-7.54E-01	1.65E-02	-3.83E-01	7.13E-04	-6.44E-01	4.45E-01	1.33E-01	4.75E-01	-1.27E-01	1.03E-01	-2.61E-01	1.79E+01
D-Lyxose	3.84E-01	3.54E-02	1.22E-01	-2.14E-01	9.99E-01	-6.62E-05	8.67E-02	-2.49E-01	8.02E-01	1.79E-02	1.22E-01	-2.31E-01	8.43E-02	-2.49E-01	4.91E+00
D-Mannitol	6.00E-01	4.10E-02	6.76E-01	-4.64E-02	1.21E-01	1.82E-01	4.84E-01	9.48E-02	5.07E-01	5.84E-02	8.04E-01	-2.91E-02	4.48E-01	-8.74E-02	4.18E+00
Docosahexaenoic acid	2.61E-01	3.58E-02	2.31E-02	-8.78E-02	1.38E-01	3.44E-02	6.20E-03	-8.92E-02	4.09E-01	2.65E-02	1.43E-02	-9.71E-02	1.38E-03	-1.24E-01	7.27E-01
Docosapentaenoic acid	2.02E-03	2.92E-01	2.26E-01	-1.06E-01	3.18E-03	2.05E-01	1.40E-02	-1.94E-01	5.22E-01	4.90E-02	5.01E-04	-3.50E-01	2.40E-05	-3.99E-01	1.76E+00
Docosenamide	3.21E-02	4.31E-01	2.52E-01	2.24E-01	5.04E-01	1.10E-01	5.80E-01	-9.73E-02	1.28E-01	2.95E-01	6.49E-01	8.79E-02	1.58E-01	-2.07E-01	1.18E+00
Eicosapentaenoic acid	6.17E-03	3.21E-01	4.27E-01	-7.75E-02	2.10E-02	2.40E-01	8.84E-02	-1.59E-01	2.38E-01	1.09E-01	2.87E-03	-2.90E-01	2.62E-05	-3.99E-01	2.65E+00
Eicosatrienoic acid	6.91E-04	4.32E-01	7.41E-01	3.50E-02	3.90E-02	2.46E-01	1.80E-01	-1.52E-01	1.00E+00	6.18E-05	1.95E-03	-3.97E-01	6.21E-04	-3.97E-01	3.18E+00
Ethanolamine	9.63E-01	2.91E-03	5.24E-01	-4.29E-02	6.11E-01	4.66E-02	9.93E-01	8.15E-04	4.33E-01	-5.76E-02	1.87E-01	-1.03E-01	4.85E-01	-4.58E-02	1.82E+01
Fumaric acid	7.10E-01	-6.06E-02	2.40E-02	-3.93E-01	5.38E-01	-1.05E-01	1.64E-02	-4.37E-01	6.41E-01	8.75E-02	1.86E-01	-2.45E-01	8.71E-02	-3.32E-01	1.02E+01
Galactose	2.56E-02	8.23E-01	4.59E-01	2.46E-01	1.91E-01	3.38E-01	4.08E-01	-2.39E-01	1.33E-01	5.50E-01	9.38E-01	-2.70E-02	3.98E-02	-5.77E-01	3.02E+00
Glutamic acid	3.64E-02	9.92E-02	1.60E-03	1.37E-01	7.73E-03	1.16E-01	8.86E-05	1.54E-01	6.97E-02	8.23E-02	3.21E-03	1.20E-01	3.53E-01	3.82E-02	3.34E+00
Glutamine	1.93E-02	1.40E+00	1.42E-02	-2.58E+00	1.14E-02	2.14E+00	3.38E-02	-1.84E+00	2.93E-02	3.01E+00	1.89E-01	-9.67E-01	6.58E-03	-3.98E+00	3.14E+00
Glutaric acid	2.47E-01	-5.89E-02	4.04E-01	4.10E-02	7.85E-01	-2.15E-02	3.32E-01	7.84E-02	7.79E-01	1.77E-02	7.57E-02	1.18E-01	4.82E-02	9.99E-02	4.91E+00
Glyceric acid	5.54E-02	2.79E-01	7.53E-02	-1.33E+00	8.88E-03	4.31E-01	9.79E-02	-1.18E+00	1.25E-02	6.45E-01	1.50E-01	-9.65E-01	4.89E-02	-1.61E+00	1.57E+01
Glycerol	5.58E-01	-4.19E-02	2.16E-03	-1.92E-01	4.77E-01	-5.81E-02	4.80E-03	-2.08E-01	8.47E-01	-1.32E-02	4.72E-03	-1.63E-01	1.67E-02	-1.50E-01	1.34E+01
Glycine	3.90E-02	1.03E+00	8.39E-03	-2.68E+00	3.18E-02	1.51E+00	1.44E-02	-2.21E+00	4.66E-02	2.01E+00	3.42E-02	-1.71E+00	4.42E-03	-3.71E+00	6.92E+00
Glycolic acid	1.95E-01	-2.22E-01	4.56E-02	-1.38E+00	7.07E-01	-5.81E-02	6.23E-02	-1.22E+00	7.18E-01	6.82E-02	8.31E-02	-1.09E+00	7.29E-02	-1.16E+00	1.98E+01
Guanosine	3.28E-02	2.23E-01	1.30E-03	2.83E-01	4.94E-01	8.26E-02	1.71E-01	1.42E-01	1.31E-01	2.16E-01	3.85E-02	2.75E-01	5.92E-01	5.96E-02	1.55E+01
Histamine	2.59E-01	-1.87E-01	4.32E-03	-1.35E+00	2.61E-01	2.56E-01	2.78E-02	-9.09E-01	7.96E-02	4.54E-01	6.89E-02	-7.11E-01	8.56E-03	-1.16E+00	1.48E+01
Histidine	7.42E-01	1.98E-02	4.78E-03	-1.98E-01	1.74E-01	-9.24E-02	2.02E-04	-3.10E-01	9.07E-01	8.10E-03	7.00E-03	-2.10E-01	3.77E-03	-2.18E-01	6.86E+00
Homovallinic acid	1.40E-01	-1.73E-01	3.19E-02	-2.28E-01	2.37E-01	1.33E-01	4.28E-01	7.85E-02	1.15E-01	2.10E-01	1.98E-01	1.55E-01	5.11E-01	-5.46E-02	7.20E+00
Hypoxanthine	1.14E-01	2.08E-01	3.20E-02	-3.98E-01	3.32E-02	2.98E-01	8.74E-02	-3.07E-01	2.04E-01	2.88E-01	1.70E-01	-3.18E-01	4.22E-03	-6.06E-01	3.99E+00
Inosine	1.07E-03	1.70E-01	1.84E-07	3.29E-01	1.01E-01	8.37E-02	4.74E-05	2.43E-01	3.60E-02	1.62E-01	3.06E-04	3.22E-01	3.95E-03	1.60E-01	4.01E+00
Isoleucine	1.07E-01	5.36E-01	3.07E-03	-1.71E+00	7.32E-02	6.61E-01	4.52E-03	-1.58E+00	3.66E-02	1.05E+00	1.95E-02	-1.19E+00	9.57E-04	-2.25E+00	7.30E+00

Lactic acid	1.33E-01	-1.59E-01	7.66E-05	-4.04E-01	3.05E-01	-1.03E-01	2.41E-04	-3.48E-01	7.94E-01	3.01E-02	3.47E-02	-2.15E-01	3.17E-02	-2.45E-01	1.19E+01
Leucine	1.03E-01	4.93E-01	2.10E-03	-1.52E+00	7.83E-02	5.71E-01	2.88E-03	-1.44E+00	3.79E-02	9.37E-01	1.67E-02	-1.08E+00	5.18E-04	-2.02E+00	3.10E+00
LPC 14:0 sn-1	4.60E-01	8.99E-02	5.13E-02	2.06E-01	7.06E-01	3.55E-02	1.82E-02	1.52E-01	9.69E-01	4.48E-03	2.00E-01	1.21E-01	2.17E-01	1.16E-01	3.77E+00
LPC 14:0 sn-2	5.67E-01	6.01E-02	4.85E-02	1.85E-01	7.16E-01	3.06E-02	1.62E-02	1.56E-01	4.44E-01	-7.18E-02	4.55E-01	5.34E-02	1.23E-01	1.25E-01	5.83E+00
LPC 16:0 sn-1	6.69E-01	2.65E-02	1.30E-01	8.34E-02	5.75E-01	-3.22E-02	6.01E-01	2.47E-02	9.65E-01	-2.71E-03	3.11E-01	5.42E-02	3.01E-01	5.69E-02	6.21E+00
LPC 16:0 sn-2	9.26E-01	-3.48E-03	8.34E-01	5.70E-03	2.82E-01	3.65E-02	4.34E-02	4.57E-02	7.82E-01	-1.04E-02	9.65E-01	-1.20E-03	7.89E-01	9.18E-03	4.63E+00
LPC 16:1 sn-1	2.47E-01	1.39E-01	7.34E-01	-3.55E-02	4.43E-01	7.49E-02	2.41E-01	-9.93E-02	6.30E-01	6.50E-02	3.73E-01	-1.09E-01	1.34E-01	-1.74E-01	3.87E+00
LPC 16:1 sn-2	1.29E-01	1.43E-01	8.51E-01	-1.34E-02	1.28E-01	1.11E-01	3.36E-01	-4.49E-02	3.24E-01	1.19E-01	7.15E-01	-3.72E-02	2.80E-02	-1.56E-01	4.14E+00
LPC 18:0 sn-1	7.76E-01	-1.21E-02	2.70E-01	4.47E-02	2.10E-01	-4.74E-02	7.80E-01	9.35E-03	2.44E-01	-5.93E-02	9.58E-01	-2.55E-03	2.04E-01	5.67E-02	9.41E+00
LPC 18:0 sn-2	7.35E-01	2.95E-02	8.16E-01	1.57E-02	6.53E-01	3.27E-02	6.93E-01	1.90E-02	6.75E-01	3.84E-02	7.36E-01	2.46E-02	8.54E-01	-1.37E-02	2.41E+00
LPC 18:1 sn-1	9.66E-01	1.82E-03	6.35E-01	1.78E-02	9.14E-01	-4.36E-03	7.41E-01	1.16E-02	7.48E-01	-1.32E-02	9.39E-01	2.78E-03	6.86E-01	1.60E-02	6.88E+00
LPC 18:1 sn-2	5.40E-01	3.10E-02	4.32E-01	3.17E-02	5.14E-01	2.67E-02	3.14E-01	2.74E-02	7.19E-01	2.12E-02	6.64E-01	2.19E-02	9.87E-01	6.89E-04	1.13E+00
LPC 18:2 sn-1	5.94E-01	6.21E-02	4.71E-01	6.62E-02	6.23E-01	4.50E-02	3.79E-01	4.91E-02	5.80E-01	6.84E-02	4.72E-01	7.26E-02	9.64E-01	4.11E-03	4.15E+00
LPC 18:2 sn-2	7.08E-01	3.29E-02	4.74E-01	5.68E-02	3.29E-01	6.37E-02	1.02E-01	8.76E-02	6.01E-01	5.17E-02	4.11E-01	7.56E-02	7.17E-01	2.39E-02	3.50E+00
LPC 20:0 sn-1	6.76E-01	3.36E-02	4.35E-01	5.49E-02	6.22E-01	-3.94E-02	7.92E-01	-1.81E-02	4.15E-01	-8.78E-02	5.04E-01	-6.65E-02	7.37E-01	2.13E-02	2.94E+00
LPC 20:0 sn-2	7.01E-01	3.26E-02	2.63E-01	1.09E-01	9.20E-01	-7.98E-03	4.54E-01	6.87E-02	2.47E-01	-1.20E-01	7.03E-01	-4.31E-02	3.51E-01	7.67E-02	4.22E+00
LPC 20:1 sn-1	9.87E-01	-1.67E-03	9.88E-01	-1.23E-03	7.35E-01	-3.17E-02	6.43E-01	-3.12E-02	9.35E-01	-9.81E-03	9.26E-01	-9.37E-03	9.96E-01	4.39E-04	3.29E+00
LPC 20:1 sn-2	9.28E-01	7.83E-03	3.85E-01	-6.89E-02	7.34E-01	-2.47E-02	1.24E-01	-1.01E-01	9.02E-01	1.37E-02	5.49E-01	-6.30E-02	2.74E-01	-7.67E-02	1.81E+00
LPC 20:4 sn-1	4.04E-01	6.36E-02	1.24E-01	8.98E-02	5.56E-01	3.73E-02	9.74E-02	6.34E-02	5.14E-01	4.80E-02	1.71E-01	7.42E-02	6.80E-01	2.61E-02	1.80E+00
LPC 20:4 sn-2	3.39E-01	8.17E-02	7.41E-02	1.44E-01	2.90E-01	6.55E-02	1.59E-02	1.28E-01	5.34E-01	5.43E-02	1.55E-01	1.16E-01	3.67E-01	6.21E-02	2.16E+00
LPC 20:5 sn-1	8.56E-03	3.95E-01	2.62E-01	1.37E-01	1.83E-01	1.57E-01	3.19E-01	-1.00E-01	8.41E-01	2.35E-02	2.99E-02	-2.34E-01	4.20E-02	-2.57E-01	7.57E+00
LPC 20:5 sn-2	9.00E-04	4.22E-01	1.15E-01	1.59E-01	1.06E-01	1.86E-01	4.51E-01	-7.60E-02	9.78E-01	4.16E-03	6.98E-02	-2.58E-01	4.64E-03	-2.62E-01	1.49E+01
LPC 22:4 sn-1	5.05E-01	8.18E-02	2.71E-01	1.17E-01	9.84E-01	2.01E-03	6.10E-01	3.71E-02	5.12E-01	8.42E-02	2.89E-01	1.19E-01	7.38E-01	3.51E-02	4.32E+00
LPC 22:4 sn-2	8.70E-01	1.56E-02	3.77E-01	7.62E-02	9.70E-01	-2.68E-03	3.09E-01	5.79E-02	6.67E-01	4.64E-02	2.89E-01	1.07E-01	4.64E-01	6.06E-02	2.90E+00
LPC 22:6 sn-1	3.73E-01	5.45E-02	3.83E-01	3.44E-02	7.91E-01	-1.58E-02	3.28E-01	-3.59E-02	9.71E-01	2.52E-03	7.24E-01	-1.76E-02	7.01E-01	-2.01E-02	7.12E+00
LPC 22:6 sn-2	4.31E-01	5.50E-02	4.54E-01	4.06E-02	3.15E-01	6.05E-02	2.63E-01	4.61E-02	6.09E-01	4.28E-02	6.88E-01	2.84E-02	8.13E-01	-1.44E-02	1.85E+00
LPE 16:0 sn-1	5.45E-01	2.77E-02	4.90E-01	2.79E-02	4.51E-01	3.33E-02	3.85E-01	3.35E-02	6.49E-01	2.09E-02	6.03E-01	2.11E-02	9.97E-01	1.90E-04	4.27E+00
LPE 16:0 sn-2	8.52E-01	-1.30E-02	6.64E-01	4.23E-02	8.75E-01	-9.02E-03	6.02E-01	4.62E-02	6.31E-01	3.39E-02	3.64E-01	8.91E-02	5.69E-01	5.52E-02	1.83E+00
LPE 16:1 sn-1	2.95E-01	1.21E-01	7.33E-01	3.36E-02	4.66E-01	7.78E-02	9.15E-01	-9.50E-03	4.93E-01	8.60E-02	9.91E-01	-1.27E-03	4.78E-01	-8.73E-02	4.66E+00
LPE 16:1 sn-2	7.76E-01	-2.68E-02	5.55E-02	-2.24E-01	7.91E-01	2.22E-02	1.02E-01	-1.75E-01	4.24E-01	1.05E-01	5.08E-01	-9.21E-02	8.74E-02	-1.98E-01	3.25E+00
LPE 18:0 sn-1	9.65E-01	2.11E-03	1.36E-01	6.40E-02	9.34E-01	3.51E-03	6.79E-02	6.54E-02	8.55E-01	8.14E-03	7.28E-02	7.01E-02	1.95E-01	6.19E-02	2.01E+00
LPE 18:0 sn-2	6.50E-01	3.90E-02	1.60E-01	1.19E-01	6.73E-01	3.41E-02	1.49E-01	1.14E-01	7.44E-01	2.78E-02	1.98E-01	1.08E-01	4.58E-01	8.01E-02	1.98E+00
LPE 18:1 sn-1	5.42E-01	3.48E-02	9.26E-01	-5.21E-03	6.89E-01	2.20E-02	7.42E-01	-1.79E-02	6.64E-01	2.73E-02	8.37E-01	-1.27E-02	5.64E-01	-4.00E-02	2.08E+00
LPE 18:1 sn-2	3.33E-01	-5.45E-02	1.97E-01	-1.03E-01	2.67E-01	-5.40E-02	1.71E-01	-1.02E-01	6.25E-01	3.14E-02	8.38E-01	-1.69E-02	5.20E-01	-4.84E-02	1.26E+00

LPE 18:2 sn-1	6.64E-01	4.14E-02	3.83E-01	7.63E-02	4.89E-01	5.74E-02	2.10E-01	9.22E-02	4.93E-01	6.64E-02	2.59E-01	1.01E-01	7.19E-01	3.49E-02	3.64E+00
LPE 18:2 sn-2	9.81E-01	-1.77E-03	7.94E-01	2.36E-02	2.97E-01	6.19E-02	2.64E-01	8.72E-02	2.74E-01	8.78E-02	2.34E-01	1.13E-01	7.65E-01	2.54E-02	3.06E+00
LPE 20:1 sn-1	6.54E-01	7.49E-02	4.47E-01	-1.14E-01	6.99E-01	6.16E-02	3.71E-01	-1.27E-01	8.33E-01	3.71E-02	3.42E-01	-1.52E-01	2.78E-01	-1.89E-01	2.16E+00
LPE 20:1 sn-2	3.35E-01	-7.96E-02	1.89E-01	-1.50E-01	2.04E-01	-1.02E-01	1.29E-01	-1.72E-01	8.33E-01	1.75E-02	6.35E-01	-5.26E-02	5.02E-01	-7.01E-02	1.32E+00
LPE 20:2 sn-1	1.81E-01	-1.41E-01	1.67E-01	-1.90E-01	2.01E-01	-1.31E-01	1.82E-01	-1.81E-01	9.27E-01	1.21E-02	8.09E-01	-3.73E-02	7.07E-01	-4.94E-02	3.34E+00
LPE 20:2 sn-2	2.86E-01	-1.15E-01	3.45E-02	3.27E-01	1.69E-01	-1.33E-01	3.12E-02	3.09E-01	1.70E-01	-1.63E-01	8.56E-02	2.79E-01	2.73E-03	4.42E-01	3.75E+00
LPE 20:4 sn-1	5.49E-01	3.36E-02	1.46E-01	9.72E-02	5.04E-01	3.24E-02	1.11E-01	9.61E-02	5.18E-01	3.56E-02	1.33E-01	9.93E-02	3.86E-01	6.36E-02	1.63E+00
LPE 20:4 sn-2	7.81E-01	-1.33E-02	6.77E-01	2.68E-02	9.87E-01	-6.66E-04	4.96E-01	3.95E-02	5.38E-01	3.39E-02	2.92E-01	7.41E-02	5.22E-01	4.02E-02	1.08E+00
LPE 22:4 sn-1	3.76E-01	1.15E-01	2.38E-01	1.47E-01	4.44E-01	9.64E-02	2.85E-01	1.28E-01	6.12E-01	7.22E-02	4.49E-01	1.04E-01	8.40E-01	3.15E-02	3.14E+00
LPE 22:4 sn-2	6.95E-01	-2.60E-02	9.08E-01	-9.94E-03	6.28E-01	-3.11E-02	8.58E-01	-1.51E-02	7.84E-01	2.02E-02	6.92E-01	3.62E-02	8.55E-01	1.60E-02	9.96E-01
LPE 22:6 sn-1	1.93E-01	7.34E-02	5.35E-01	3.44E-02	8.35E-01	1.03E-02	5.67E-01	-2.87E-02	3.31E-01	5.97E-02	7.33E-01	2.07E-02	5.34E-01	-3.90E-02	8.05E+00
LPE 22:6 sn-2	4.96E-01	-2.91E-02	6.44E-01	-3.61E-02	3.58E-01	4.37E-02	6.43E-01	3.67E-02	3.25E-01	3.46E-02	7.04E-01	2.77E-02	9.23E-01	-6.98E-03	1.26E+01
LPE P-16:0	4.92E-01	5.64E-02	4.22E-02	1.70E-01	5.39E-01	4.98E-02	4.74E-02	1.64E-01	9.32E-01	7.07E-03	1.43E-01	1.21E-01	2.85E-01	1.14E-01	1.99E+00
LPE P-18:0	5.53E-01	4.22E-02	5.60E-02	1.42E-01	5.98E-01	3.66E-02	5.95E-02	1.36E-01	9.52E-01	4.44E-03	1.68E-01	1.04E-01	2.88E-01	9.96E-02	1.90E+00
LPE P-18:1	7.78E-01	2.44E-02	6.17E-01	-3.68E-02	8.07E-01	2.08E-02	5.75E-01	-4.04E-02	5.25E-01	5.79E-02	9.66E-01	-3.31E-03	5.36E-01	-6.12E-02	2.09E+00
LPE P-20:0	4.70E-01	8.98E-02	2.22E-02	3.19E-01	7.36E-01	3.86E-02	3.52E-02	2.68E-01	8.21E-01	-2.98E-02	1.61E-01	1.99E-01	1.34E-01	2.29E-01	5.50E+00
LPE P-20:1	1.00E+00	6.97E-05	6.60E-01	-5.41E-02	8.01E-01	-3.13E-02	4.82E-01	-8.55E-02	9.01E-01	1.75E-02	7.90E-01	-3.67E-02	6.97E-01	-5.42E-02	6.34E+00
LPE P-22:1	9.92E-01	1.38E-03	5.51E-01	8.93E-02	4.61E-01	-9.19E-02	9.75E-01	-4.06E-03	5.23E-01	-8.35E-02	9.75E-01	4.35E-03	5.15E-01	8.79E-02	7.77E+00
LPG 16:0 sn-1	9.97E-01	-2.72E-04	1.04E-01	-1.29E-01	9.22E-01	5.16E-03	5.88E-02	-1.24E-01	4.62E-01	7.89E-02	6.42E-01	-5.02E-02	7.75E-02	-1.29E-01	1.49E+00
LPG 16:0 sn-2	9.39E-01	-5.53E-03	9.29E-02	-1.40E-01	6.02E-01	2.47E-02	8.06E-02	-1.09E-01	5.35E-01	5.96E-02	4.56E-01	-7.45E-02	4.57E-02	-1.34E-01	2.82E+00
LPG 18:0 sn-1	9.94E-01	4.81E-04	9.23E-02	-1.20E-01	8.88E-01	6.17E-03	4.03E-02	-1.14E-01	4.70E-01	7.38E-02	6.50E-01	-4.64E-02	5.94E-02	-1.20E-01	2.99E+00
LPG 18:0 sn-2	4.74E-01	-4.65E-02	6.42E-02	-1.67E-01	1.99E-01	6.20E-02	4.28E-01	-5.84E-02	4.86E-01	7.26E-02	6.77E-01	-4.78E-02	1.44E-01	-1.20E-01	7.21E+00
LPG 18:1 sn-1	4.54E-01	6.11E-02	2.52E-02	-1.78E-01	4.83E-01	4.17E-02	1.53E-03	-1.97E-01	9.72E-02	2.31E-01	9.49E-01	-7.92E-03	1.90E-03	-2.39E-01	2.17E+00
LPG 18:1 sn-2	5.62E-01	3.70E-02	5.31E-02	-2.03E-01	4.58E-01	3.70E-02	3.90E-02	-2.03E-01	3.68E-01	1.05E-01	3.16E-01	-1.35E-01	2.58E-02	-2.40E-01	3.45E+00
LPG 20:4 sn-1	2.04E-01	1.26E-01	3.90E-01	-7.37E-02	3.73E-01	6.56E-02	3.72E-02	-1.34E-01	1.28E-01	2.17E-01	8.87E-01	1.79E-02	1.67E-02	-1.99E-01	1.65E+00
LPG 20:4 sn-2	5.26E-01	6.68E-02	2.12E-01	-1.42E-01	1.51E-01	1.21E-01	3.46E-01	-8.87E-02	9.26E-02	2.28E-01	8.88E-01	1.86E-02	5.66E-02	-2.09E-01	7.00E+00
LPG 22:6 sn-1	2.87E-01	1.04E-01	8.50E-04	-2.92E-01	1.93E-01	1.10E-01	1.58E-04	-2.85E-01	2.91E-02	3.46E-01	6.90E-01	-4.97E-02	5.16E-05	-3.95E-01	2.56E+00
LPG 22:6 sn-2	2.33E-01	8.77E-02	2.87E-03	-2.83E-01	4.30E-01	5.44E-02	9.25E-04	-3.17E-01	4.97E-02	2.71E-01	4.32E-01	-9.97E-02	2.59E-04	-3.71E-01	5.04E+00
LPI 16:0 sn-1	1.25E-03	-2.16E-01	1.51E-02	-1.38E-01	8.61E-02	-1.17E-01	5.36E-01	-3.81E-02	3.45E-01	-5.65E-02	6.88E-01	2.20E-02	1.19E-01	7.85E-02	1.58E+00
LPI 16:0 sn-2	5.20E-02	-1.61E-01	8.99E-02	-1.34E-01	1.18E-01	-1.17E-01	2.04E-01	-9.08E-02	8.98E-01	-9.87E-03	8.23E-01	1.67E-02	7.18E-01	2.66E-02	3.42E+00
LPI 18:0 sn-1	1.05E-02	-1.41E-01	4.88E-01	-4.00E-02	1.55E-01	-7.81E-02	7.03E-01	2.30E-02	1.79E-01	-5.82E-02	3.93E-01	4.29E-02	6.92E-02	1.01E-01	1.57E+00
LPI 18:0 sn-2	2.55E-02	-2.00E-01	4.75E-01	-5.98E-02	1.76E-01	-1.17E-01	7.88E-01	2.28E-02	2.68E-01	-8.58E-02	4.75E-01	5.43E-02	1.28E-01	1.40E-01	1.26E+00
LPI 18:1 sn-1	6.55E-02	-1.31E-01	2.75E-01	-6.19E-02	3.79E-01	-6.52E-02	9.49E-01	4.05E-03	9.88E-01	-1.18E-03	3.54E-01	6.81E-02	2.44E-01	6.92E-02	1.02E+00
LPI 18:1 sn-2	5.68E-02	-1.45E-01	2.04E-01	-8.69E-02	3.95E-01	-6.64E-02	9.05E-01	-8.67E-03	6.45E-01	-3.54E-02	7.59E-01	2.23E-02	4.07E-01	5.77E-02	1.71E+00

LPI 20:4 sn-1	7.07E-01	-2.69E-02	6.62E-01	2.35E-02	8.56E-01	-1.15E-02	3.59E-01	3.89E-02	7.33E-01	2.60E-02	2.18E-01	7.64E-02	4.15E-01	5.04E-02	5.59E-01
LPI 20:4 sn-2	9.59E-02	-1.08E-01	9.61E-01	-3.49E-03	6.33E-01	-3.40E-02	3.84E-01	7.02E-02	6.96E-01	-2.69E-02	3.27E-01	7.73E-02	1.38E-01	1.04E-01	2.15E+00
LPI 22:6 sn-1	1.44E-01	-1.35E-01	7.32E-03	-1.99E-01	3.11E-01	-8.95E-02	2.63E-02	-1.54E-01	9.42E-01	7.79E-03	5.30E-01	-5.70E-02	4.28E-01	-6.48E-02	1.78E+00
LPI 22:6 sn-2	9.13E-03	-2.03E-01	2.42E-02	-1.94E-01	3.41E-02	-1.57E-01	7.26E-02	-1.48E-01	4.51E-01	-5.78E-02	5.62E-01	-4.91E-02	9.01E-01	8.74E-03	3.61E+00
LPS 18:0 sn-1	4.69E-01	-8.76E-02	7.25E-01	-4.80E-02	3.49E-01	-1.11E-01	5.95E-01	-7.14E-02	8.93E-01	-1.63E-02	8.65E-01	2.33E-02	7.92E-01	3.96E-02	6.84E+00
LPS 18:0 sn-2	6.21E-01	-9.16E-02	7.67E-01	-5.80E-02	2.94E-01	-1.96E-01	4.11E-01	-1.62E-01	7.73E-01	-5.24E-02	9.22E-01	-1.88E-02	8.78E-01	3.36E-02	1.06E+01
LPS 18:1 sn-1	3.27E-01	-1.22E-01	1.85E-01	-1.47E-01	2.64E-01	-1.46E-01	1.47E-01	-1.70E-01	9.36E-01	1.09E-02	9.13E-01	-1.34E-02	8.52E-01	-2.43E-02	3.94E+00
LPS 18:1 sn-2	2.54E-02	-1.84E-01	4.32E-02	-2.26E-01	5.29E-02	-1.61E-01	6.94E-02	-2.03E-01	6.37E-01	-3.52E-02	4.52E-01	-7.65E-02	6.86E-01	-4.13E-02	3.42E+00
Lysine	7.72E-01	3.02E-02	9.39E-03	-2.77E-01	9.62E-01	6.08E-03	1.72E-02	-3.01E-01	8.91E-01	1.20E-02	2.00E-03	-2.95E-01	4.91E-03	-3.07E-01	7.90E+00
Malic acid	8.96E-01	1.95E-02	2.73E-03	-4.18E-01	9.93E-01	1.25E-03	9.00E-04	-4.36E-01	2.66E-01	1.88E-01	8.40E-02	-2.49E-01	5.88E-03	-4.38E-01	4.68E+00
Malonic acid	4.74E-01	-8.98E-02	7.24E-03	-6.64E-01	2.09E-01	1.46E-01	4.90E-02	-4.28E-01	6.90E-01	5.12E-02	2.46E-02	-5.23E-01	1.19E-02	-5.74E-01	2.50E+01
Methionine	1.33E-01	4.83E-01	1.13E-03	-1.51E+00	9.31E-02	5.19E-01	1.22E-03	-1.47E+00	6.13E-02	9.23E-01	1.40E-02	-1.07E+00	2.66E-04	-1.99E+00	5.27E+00
Methylamine	3.41E-03	6.08E-01	1.78E-02	-7.92E-01	7.73E-03	6.05E-01	1.92E-02	-7.95E-01	2.89E-04	1.00E+00	1.98E-01	-3.99E-01	9.20E-04	-1.40E+00	1.32E+01
MG 14:0 (1)	2.05E-05	3.89E-01	9.26E-06	5.37E-01	2.19E-05	3.50E-01	1.14E-05	4.99E-01	1.75E-01	5.57E-02	1.04E-02	2.04E-01	5.23E-02	1.48E-01	3.27E+00
MG 16:0 (1)	6.65E-01	4.01E-02	7.73E-02	-1.79E-01	4.24E-01	5.56E-02	4.86E-02	-1.64E-01	4.01E-01	7.90E-02	1.62E-01	-1.40E-01	7.98E-03	-2.19E-01	5.31E+00
MG 16:0 (2)	6.37E-01	4.01E-02	1.67E-01	-1.33E-01	3.62E-01	5.97E-02	1.57E-01	-1.13E-01	6.55E-01	4.05E-02	1.87E-01	-1.32E-01	3.07E-02	-1.73E-01	4.09E+00
MG 18:0 (1)	3.44E-01	-1.19E-01	1.97E-01	-1.72E-01	7.87E-01	3.64E-02	9.06E-01	-1.64E-02	6.74E-01	7.53E-02	9.01E-01	2.24E-02	6.34E-01	-5.28E-02	3.02E+00
MG 18:0 (2)	1.03E-01	1.12E-01	5.08E-03	2.17E-01	1.18E-02	2.71E-01	1.48E-03	3.76E-01	2.86E-01	1.19E-01	6.16E-02	2.24E-01	2.26E-01	1.05E-01	1.31E+01
MG 18:1 (1)	3.58E-05	3.06E-01	2.02E-06	4.20E-01	1.38E-03	3.22E-01	1.35E-04	4.37E-01	1.83E-01	1.39E-01	2.81E-02	2.54E-01	1.38E-01	1.15E-01	7.70E+00
MG 18:1 (2)	9.40E-01	-7.43E-03	5.19E-02	-1.57E-01	8.45E-01	-2.09E-02	5.92E-02	-1.71E-01	5.53E-01	9.71E-02	7.19E-01	-5.27E-02	1.40E-01	-1.50E-01	3.76E+00
MG 18:2 (2)	8.64E-01	1.97E-02	9.85E-01	-1.22E-03	2.18E-01	1.79E-01	1.65E-01	1.58E-01	2.90E-01	1.91E-01	2.75E-01	1.70E-01	8.51E-01	-2.09E-02	2.08E+01
MG 20:4 (1)	1.24E-01	2.50E-01	4.09E-01	1.33E-01	3.89E-01	1.39E-01	8.91E-01	2.24E-02	2.13E-01	3.72E-01	3.77E-01	2.55E-01	3.26E-01	-1.17E-01	2.92E+00
MG 20:4 (2)	5.54E-02	3.44E-01	2.81E-01	1.79E-01	3.43E-01	1.70E-01	9.77E-01	4.92E-03	1.52E-01	4.24E-01	3.49E-01	2.59E-01	2.10E-01	-1.65E-01	2.73E+00
MG 22:6 (1)	1.77E-01	1.21E-01	8.23E-01	-2.16E-02	1.85E-01	1.17E-01	7.90E-01	-2.55E-02	1.52E-01	2.09E-01	6.44E-01	6.64E-02	1.25E-01	-1.43E-01	5.03E+00
MG 22:6 (2)	5.61E-02	2.11E-01	8.40E-01	-2.06E-02	7.29E-02	1.92E-01	6.97E-01	-3.91E-02	1.38E-01	2.47E-01	9.16E-01	1.61E-02	4.79E-02	-2.31E-01	2.55E+00
Monoisoamylamine	2.67E-03	-5.18E-01	1.54E-02	-7.57E-01	4.12E-01	-1.16E-01	2.15E-01	-3.55E-01	5.32E-01	-1.55E-01	2.46E-01	-3.94E-01	3.22E-01	-2.39E-01	2.00E+01
Myo-Inositol	2.63E-01	3.22E-02	8.81E-01	3.92E-03	6.53E-01	-2.37E-02	3.15E-01	-5.20E-02	4.46E-01	-4.50E-02	2.09E-01	-7.32E-02	1.98E-01	-2.83E-02	2.56E+00
NAA (N-acetylaspartate)	4.10E-01	5.50E-02	1.49E-05	6.73E-01	1.57E-01	1.12E-01	1.31E-05	7.30E-01	2.42E-02	1.94E-01	3.77E-06	8.12E-01	1.30E-04	6.18E-01	3.94E+00
NAAG (N-acetyl-aspartyl-glutamate)	3.05E-01	6.93E-02	8.99E-01	1.03E-02	2.28E-02	-1.59E-01	1.41E-02	-2.18E-01	3.76E-01	1.19E-01	6.66E-01	5.97E-02	3.57E-01	-5.91E-02	2.31E+00
N-Acetyl-L-glutamic acid	1.52E-02	2.03E-01	3.44E-01	7.20E-02	8.99E-02	2.01E-01	5.26E-01	7.01E-02	7.52E-01	3.28E-02	3.35E-01	-9.82E-02	2.80E-02	-1.31E-01	9.87E+00
N-Acetylneuraminic Acid	8.49E-01	1.11E-02	5.86E-02	-1.08E-01	3.76E-01	-4.38E-02	1.72E-03	-1.63E-01	2.53E-01	6.65E-02	3.26E-01	-5.30E-02	2.88E-02	-1.19E-01	3.43E+00
Nervonic acid	6.62E-01	2.11E-02	2.22E-01	5.45E-02	1.03E-02	2.07E-01	3.06E-03	2.41E-01	4.59E-01	4.72E-02	1.92E-01	8.06E-02	5.09E-01	3.34E-02	8.95E+00
Nicotinamide	6.63E-05	1.46E-01	5.76E-03	1.23E-01	1.85E-01	6.46E-02	4.59E-01	4.11E-02	6.29E-02	1.39E-01	1.40E-01	1.15E-01	4.95E-01	-2.35E-02	5.90E+00
Nicotinic acid	6.86E-02	1.20E+00	4.50E-02	-3.38E+00	3.36E-01	9.43E-01	4.21E-02	-3.63E+00	7.63E-01	1.78E-01	3.55E-02	-4.40E+00	4.51E-02	-4.57E+00	2.33E+01

N-oleoyl taurine	6.02E-01	4.91E-02	6.97E-02	-1.65E-01	7.45E-02	1.39E-01	2.85E-01	-7.53E-02	7.47E-01	-3.33E-02	1.73E-02	-2.48E-01	7.42E-03	-2.14E-01	1.74E+00
Nonanoic acid	8.77E-02	1.26E-01	1.33E-02	2.07E-01	5.61E-02	2.09E-01	1.48E-02	2.90E-01	4.14E-01	6.66E-02	1.01E-01	1.47E-01	2.11E-01	8.09E-02	1.16E+01
Octadecadienoic acid	2.76E-02	1.95E-01	5.00E-01	5.30E-02	1.51E-02	2.66E-01	2.05E-01	1.23E-01	1.38E-01	1.34E-01	9.19E-01	-8.43E-03	3.90E-02	-1.42E-01	3.41E+00
Oleic acid	3.68E-02	1.80E-01	3.17E-01	-7.97E-02	2.62E-01	7.29E-02	8.76E-03	-1.87E-01	2.21E-01	9.15E-02	2.95E-02	-1.68E-01	1.80E-03	-2.60E-01	1.68E+00
Orthophosphate	2.76E-01	-1.18E-01	3.17E-01	-7.93E-02	7.05E-01	-3.99E-02	9.86E-01	-1.39E-03	4.66E-01	-7.15E-02	6.19E-01	-3.30E-02	7.21E-01	3.85E-02	9.40E+00
Palmitic acid	1.22E-03	3.66E-01	1.43E-01	-1.61E-01	1.12E-02	2.63E-01	2.16E-02	-2.63E-01	2.27E-01	1.48E-01	6.31E-03	-3.79E-01	4.83E-05	-5.27E-01	5.15E+00
Palmitoylcarnitine	4.51E-02	-1.73E-01	6.35E-04	-3.15E-01	3.27E-01	-7.37E-02	6.15E-03	-2.16E-01	4.22E-01	1.07E-01	7.82E-01	-3.49E-02	1.07E-01	-1.42E-01	2.34E+00
Pantothenate	7.00E-01	-3.16E-02	8.83E-01	-1.40E-02	1.82E-02	-1.62E-01	8.80E-02	-1.44E-01	2.12E-01	1.16E-01	2.02E-01	1.34E-01	7.82E-01	1.76E-02	3.44E+00
Phenylalanine	4.60E-02	6.69E-01	2.72E-03	-1.39E+00	4.25E-02	7.40E-01	3.91E-03	-1.32E+00	2.63E-02	1.03E+00	1.78E-02	-1.02E+00	3.51E-04	-2.06E+00	3.19E+00
Phosphocholine	4.65E-03	1.22E-01	3.36E-03	1.42E-01	9.67E-02	6.56E-02	5.58E-02	8.58E-02	5.79E-03	1.31E-01	4.00E-03	1.51E-01	6.03E-01	2.02E-02	1.61E+00
Phosphorylethanolamine	4.27E-01	5.68E-02	3.02E-02	2.14E-01	1.82E-02	1.68E-01	1.28E-03	3.26E-01	2.86E-03	1.54E-01	3.02E-04	3.11E-01	7.47E-02	1.57E-01	8.16E+00
Pimelic acid	2.17E-02	5.11E-01	1.22E-01	4.08E-01	4.45E-02	5.56E-01	1.39E-01	4.53E-01	1.58E-01	3.89E-01	3.57E-01	2.86E-01	7.03E-01	-1.03E-01	3.09E+01
Pipecolic acid	2.06E-01	-1.24E-01	1.80E-03	-3.07E-01	8.84E-03	-2.20E-01	5.64E-06	-4.02E-01	9.30E-01	-8.10E-03	3.06E-02	-1.91E-01	3.50E-02	-1.83E-01	4.84E+00
Proline	9.34E-01	4.68E-03	2.69E-02	-1.25E-01	1.72E-01	-7.53E-02	6.10E-04	-2.05E-01	8.15E-01	-1.45E-02	2.17E-02	-1.45E-01	3.91E-02	-1.30E-01	3.68E+00
Putrescine	5.39E-01	-9.32E-02	2.08E-02	2.91E-01	1.71E-01	2.24E-01	3.82E-04	6.08E-01	7.95E-01	-4.08E-02	1.40E-02	3.43E-01	2.77E-02	3.84E-01	2.16E+01
Pyridoxine	9.81E-02	9.23E-01	3.76E-03	-1.55E+00	5.99E-02	1.04E+00	5.50E-03	-1.43E+00	1.31E-01	1.69E+00	1.89E-01	-7.86E-01	4.67E-04	-2.47E+00	1.60E+01
Pyroglutamic acid	1.07E-01	6.13E-01	1.62E-02	-9.58E-01	2.84E-02	8.17E-01	4.09E-02	-7.53E-01	5.63E-02	8.96E-01	8.76E-02	-6.74E-01	9.82E-04	-1.57E+00	7.07E+00
Pyruvic acid	2.29E-02	-4.30E-01	9.26E-03	-5.72E-01	4.97E-01	-1.12E-01	1.79E-01	-2.53E-01	9.71E-01	7.67E-03	5.42E-01	-1.34E-01	4.98E-01	-1.42E-01	9.73E+00
Riboflavine	1.16E-02	1.01E+00	7.80E-03	-9.79E-01	7.25E-02	8.08E-01	3.98E-03	-1.18E+00	1.49E-03	1.17E+00	1.45E-02	-8.22E-01	9.39E-05	-1.99E+00	6.95E+00
Ribose	7.70E-02	-3.63E-01	3.05E-04	-1.32E+00	4.53E-01	-1.59E-01	1.09E-03	-1.12E+00	7.85E-01	8.12E-02	1.12E-02	-8.76E-01	2.32E-03	-9.58E-01	2.52E+01
S-Adenosyl-L-homocysteine	8.54E-01	-1.73E-02	5.03E-03	6.11E-01	2.09E-01	-9.36E-02	8.49E-03	5.35E-01	7.40E-01	4.89E-02	9.46E-03	6.77E-01	1.61E-03	6.29E-01	3.67E+00
Serine	7.04E-02	6.80E-01	1.20E-02	-2.86E+00	4.48E-02	1.20E+00	1.91E-02	-2.34E+00	3.57E-02	1.83E+00	4.47E-02	-1.72E+00	8.24E-03	-3.54E+00	1.14E+01
Stearic acid	7.64E-01	2.08E-02	6.28E-01	-2.96E-02	7.86E-02	1.72E-01	1.76E-01	1.21E-01	2.23E-01	9.92E-02	5.04E-01	4.87E-02	4.12E-01	-5.05E-02	3.50E+00
Succinic acid	4.20E-01	1.43E-01	2.95E-02	4.18E-01	5.31E-01	1.03E-01	3.18E-02	3.78E-01	5.81E-01	1.02E-01	5.83E-02	3.77E-01	1.60E-01	2.76E-01	5.46E+00
Tartaric acid	7.20E-01	7.02E-02	1.46E-01	-2.68E-01	4.36E-01	1.29E-01	1.81E-01	-2.09E-01	8.51E-01	-3.74E-02	5.11E-02	-3.76E-01	5.96E-02	-3.38E-01	1.15E+01
Taurine	5.10E-02	7.28E-01	4.57E-03	-1.57E+00	1.72E-02	1.08E+00	1.55E-02	-1.22E+00	3.53E-02	1.29E+00	4.44E-02	-1.01E+00	8.59E-04	-2.30E+00	3.82E+00
Taurocholic acid	7.04E-01	5.30E-01	6.59E-01	5.68E-01	9.80E-01	-3.48E-02	9.98E-01	3.59E-03	4.11E-01	9.31E-01	3.54E-01	9.70E-01	9.79E-01	3.84E-02	4.82E+00
Thiamine	5.10E-01	1.42E-01	3.56E-03	-7.06E-01	5.14E-01	1.56E-01	6.10E-03	-6.92E-01	2.86E-01	3.11E-01	4.46E-02	-5.37E-01	1.12E-03	-8.48E-01	7.24E+00
Threonine	2.31E-02	1.08E+00	9.38E-03	-2.64E+00	2.04E-02	1.45E+00	1.39E-02	-2.27E+00	2.91E-02	2.13E+00	4.26E-02	-1.59E+00	4.78E-03	-3.72E+00	8.51E+00
Thymidine	7.84E-02	4.57E-01	2.47E-01	-3.35E-01	1.32E-01	4.19E-01	2.19E-01	-3.73E-01	7.19E-02	6.54E-01	6.79E-01	-1.38E-01	2.17E-02	-7.92E-01	5.33E+00
Thymine	2.18E-01	3.00E-01	4.48E-02	-5.52E-01	2.79E-01	3.20E-01	7.67E-02	-5.31E-01	1.60E-01	5.01E-01	2.73E-01	-3.50E-01	7.84E-03	-8.51E-01	6.83E+00
Trimethylamine	3.50E-01	4.21E-01	1.99E-01	-5.93E-01	6.22E-01	1.61E-01	5.39E-02	-8.52E-01	6.73E-01	1.72E-01	8.50E-02	-8.41E-01	2.77E-02	-1.01E+00	6.69E+00
Trimethylamine-n-oxide	2.06E-03	-1.15E+00	2.17E-01	-4.48E-01	8.09E-04	-1.27E+00	1.13E-01	-5.62E-01	5.92E-01	1.87E-01	4.13E-02	8.92E-01	3.36E-02	7.05E-01	7.76E+00
Trimethyllysine N6,N6,N6	7.76E-01	-1.76E-02	6.42E-01	2.16E-02	7.80E-01	-1.72E-02	6.33E-01	2.20E-02	1.33E-01	1.27E-01	3.23E-02	1.66E-01	4.82E-01	3.92E-02	4.05E+00

Tryptamine	9.11E-01	-3.45E-02	2.28E-02	-1.09E+00	9.85E-01	6.00E-03	2.75E-02	-1.05E+00	3.38E-01	3.05E-01	8.15E-02	-7.54E-01	2.29E-02	-1.06E+00	1.32E+01
Tryptophan	2.51E-02	8.94E-01	4.62E-03	-1.48E+00	2.83E-02	1.02E+00	7.90E-03	-1.35E+00	1.50E-02	1.35E+00	3.18E-02	-1.02E+00	5.47E-04	-2.37E+00	3.72E+00
Tyrosine	4.08E-02	9.79E-01	2.63E-03	-2.05E+00	1.89E-02	1.24E+00	4.71E-03	-1.79E+00	8.24E-02	1.44E+00	1.23E-02	-1.59E+00	6.52E-04	-3.03E+00	3.39E+00
Uracil	1.14E-02	-3.44E-01	1.31E-04	-5.24E-01	2.54E-01	-1.43E-01	8.15E-03	-3.23E-01	6.73E-01	-3.56E-02	6.61E-03	-2.15E-01	6.96E-02	-1.80E-01	8.56E+00
Uridine	1.77E-03	1.53E-01	4.60E-06	3.06E-01	3.69E-01	4.23E-02	1.41E-03	1.95E-01	4.24E-01	6.88E-02	2.51E-02	2.21E-01	4.17E-04	1.52E-01	4.23E+00
Valine	1.41E-01	-2.07E-01	5.47E-03	-3.72E-01	1.66E-01	-2.20E-01	1.10E-02	-3.85E-01	2.78E-01	-1.41E-01	1.24E-02	-3.06E-01	2.00E-01	-1.65E-01	1.91E+01
Xanthine	4.85E-02	7.05E-01	5.76E-03	-1.25E+00	2.25E-02	9.96E-01	2.43E-02	-9.57E-01	8.58E-03	1.23E+00	6.94E-02	-7.27E-01	7.09E-04	-1.95E+00	4.24E+00
γ-Aminobutyric acid	8.63E-02	4.09E-01	3.47E-03	-1.52E+00	2.02E-02	8.20E-01	1.69E-02	-1.11E+00	3.58E-02	1.08E+00	6.69E-02	-8.47E-01	1.18E-03	-1.93E+00	8.05E+00

Table S3. Extracted p(corr) and VIP values from each statistically significant pairwise comparison based on OPLS discriminant analysis. VIP values ≥1.50 are marked in red.

Comparisons	1v4		1v5		2v4		2v5		3v5		4v5	
**Compounds**	**p(corr)**	**VIP**	**p(corr)**	**VIP**	**p(corr)**	**VIP**	**p(corr)**	**VIP**	**p(corr)**	**VIP**	**p(corr)**	**VIP**
1,5-Anhydroglucitol	-2.30E-01	6.50E-01	1.50E-01	3.10E-01	-3.70E-01	1.07E+00	1.00E-02	2.00E-02	1.00E-01	2.30E-01	3.90E-01	8.90E-01
2,3-Butanediol	-5.20E-01	1.36E+00	-5.50E-01	1.20E+00	-8.00E-02	2.50E-01	-4.00E-02	2.00E-01	-1.80E-01	4.20E-01	-3.00E-02	1.00E-02
2-Hydroxybutyric acid	3.80E-01	1.08E+00	6.90E-01	1.57E+00	2.90E-01	8.40E-01	6.00E-01	1.48E+00	-3.00E-02	1.30E-01	3.10E-01	6.90E-01
2-Ketoglutaric acid	-3.60E-01	9.10E-01	-2.20E-01	4.60E-01	-2.40E-01	6.30E-01	-1.60E-01	3.70E-01	-3.60E-01	9.90E-01	-4.00E-02	0.00E+00
2-Hydroxyisovaleric acid	-3.10E-01	8.40E-01	4.00E-01	9.00E-01	-4.30E-01	1.16E+00	2.50E-01	6.70E-01	6.80E-01	1.92E+00	6.00E-01	1.43E+00
3,4-Dihydroxyphenylacetic acid	7.00E-02	2.20E-01	7.30E-01	1.63E+00	-4.20E-01	1.22E+00	2.00E-01	4.30E-01	1.30E-01	2.30E-01	5.10E-01	1.27E+00
3-Hydroxy-3-methylglutaric acid	4.60E-01	1.27E+00	4.40E-01	1.01E+00	-1.10E-01	2.40E-01	5.00E-02	3.00E-02	-4.00E-02	1.50E-01	9.00E-02	2.00E-01
3-Hydroxybutyric acid	7.70E-01	2.01E+00	8.30E-01	1.87E+00	5.00E-01	1.47E+00	5.60E-01	1.29E+00	2.00E-02	1.00E-01	-1.20E-01	3.00E-01
3-Methyl-2-oxovaleric acid	6.10E-01	1.62E+00	4.40E-01	1.04E+00	1.60E-01	5.50E-01	9.00E-02	2.10E-01	4.00E-02	1.10E-01	-1.10E-01	1.40E-01
4-Hydroxybenzoic acid	-7.30E-01	1.97E+00	-4.40E-01	9.90E-01	-6.70E-01	1.93E+00	-3.90E-01	1.00E+00	-3.00E-02	1.40E-01	4.30E-01	1.00E+00
4-Hydroxyphenyllactic acid	-5.60E-01	1.46E+00	-5.00E-01	1.17E+00	-3.60E-01	1.00E+00	-2.50E-01	6.80E-01	-1.50E-01	5.50E-01	1.40E-01	3.10E-01
5-Hydroxyindole-3-acetic acid	3.30E-01	9.80E-01	7.10E-01	1.63E+00	-2.20E-01	6.00E-01	8.00E-02	1.10E-01	-3.50E-01	1.08E+00	2.80E-01	6.80E-01
5-Methylthioadenosine	-6.60E-01	1.68E+00	-8.90E-01	1.97E+00	-3.10E-01	8.80E-01	-8.00E-01	1.93E+00	-7.20E-01	2.17E+00	-7.60E-01	1.72E+00
Acetylcarnitine	8.60E-01	2.31E+00	4.80E-01	1.08E+00	7.00E-01	2.13E+00	2.00E-02	1.10E-01	-3.80E-01	1.17E+00	-7.30E-01	1.66E+00
Aconitic acid	-3.00E-01	6.90E-01	1.00E-02	5.00E-02	-4.90E-01	1.41E+00	-2.60E-01	6.80E-01	2.00E-02	7.00E-02	2.00E-01	5.40E-01
Adenosine	4.90E-01	1.36E+00	3.00E-02	1.10E-01	-7.00E-02	1.20E-01	-4.90E-01	1.15E+00	-1.10E-01	5.50E-01	-4.90E-01	1.02E+00
Adenosine monophosphate	-2.60E-01	7.00E-01	-5.50E-01	1.18E+00	1.00E-02	5.00E-02	-2.50E-01	6.40E-01	-2.20E-01	5.00E-01	-4.10E-01	9.60E-01
Adrenic acid	-3.10E-01	8.60E-01	2.60E-01	5.50E-01	-2.00E-01	6.00E-01	4.60E-01	1.09E+00	3.10E-01	9.70E-01	5.20E-01	1.17E+00
Alanine	-5.50E-01	1.42E+00	6.30E-01	1.42E+00	-6.30E-01	1.86E+00	5.60E-01	1.34E+00	5.00E-01	1.34E+00	7.60E-01	1.79E+00
alpha-D-Lyxopyranose	5.60E-01	1.44E+00	5.60E-01	1.33E+00	2.00E-02	1.00E-02	1.10E-01	2.40E-01	8.00E-02	8.00E-02	2.20E-01	5.60E-01
alpha-Linolenic acid	-2.80E-01	8.00E-01	5.00E-02	1.50E-01	-4.70E-01	1.40E+00	-2.30E-01	6.40E-01	1.20E-01	2.40E-01	4.30E-01	9.80E-01

alpha-Tocopherol	3.20E-01	7.70E-01	1.60E-01	3.90E-01	-1.60E-01	4.90E-01	-2.40E-01	6.40E-01	-2.30E-01	8.10E-01	-1.60E-01	3.30E-01
Arabinitol	-2.00E-02	4.00E-02	1.00E-01	2.60E-01	-1.40E-01	4.00E-01	-1.00E-02	1.30E-01	-1.00E-02	2.30E-01	1.60E-01	4.00E-01
Arachidonic acid	7.80E-01	2.04E+00	8.70E-01	1.97E+00	-6.00E-02	2.10E-01	-3.00E-02	1.90E-01	-1.80E-01	5.30E-01	-3.00E-02	1.00E-02
Arachidonoylcarnitine	-7.40E-01	1.98E+00	-3.60E-01	8.30E-01	-6.00E-01	1.67E+00	-5.00E-02	1.30E-01	-7.00E-02	1.90E-01	5.00E-01	1.18E+00
Arginine	3.80E-01	9.90E-01	8.00E-01	1.76E+00	4.90E-01	1.41E+00	8.40E-01	1.96E+00	8.10E-01	2.41E+00	6.30E-01	1.34E+00
Asparagine	-6.10E-01	1.58E+00	5.80E-01	1.30E+00	-6.70E-01	1.96E+00	5.00E-01	1.18E+00	4.40E-01	1.12E+00	7.00E-01	1.65E+00
Aspartic acid	4.70E-01	1.29E+00	8.40E-01	1.83E+00	3.10E-01	9.70E-01	7.20E-01	1.66E+00	6.80E-01	2.04E+00	5.70E-01	1.21E+00
Azelaic acid	-1.30E-01	2.20E-01	-3.20E-01	7.30E-01	-4.40E-01	1.17E+00	-6.10E-01	1.52E+00	-6.70E-01	1.97E+00	-1.90E-01	3.60E-01
Benzoic acid	3.60E-01	9.40E-01	5.80E-01	1.29E+00	-6.00E-02	1.40E-01	9.00E-02	1.80E-01	4.00E-02	1.00E-02	1.00E-01	2.70E-01
beta-Arabinopyranose	-4.40E-01	1.15E+00	-1.40E-01	1.80E-01	-2.20E-01	6.90E-01	-3.00E-02	1.10E-01	2.20E-01	7.10E-01	2.30E-01	6.80E-01
Betaine	-9.00E-02	1.40E-01	6.50E-01	1.48E+00	1.90E-01	5.80E-01	7.50E-01	1.83E+00	3.10E-01	9.00E-01	6.60E-01	1.56E+00
Carnitine	-8.40E-01	2.24E+00	-8.00E-01	1.78E+00	-2.90E-01	7.00E-01	-6.00E-02	1.60E-01	-2.00E-01	5.80E-01	2.40E-01	5.70E-01
Cholesterol	-3.20E-01	9.30E-01	-5.00E-01	1.11E+00	-2.90E-01	8.50E-01	-4.30E-01	1.08E+00	-7.00E-02	3.60E-01	-2.20E-01	5.00E-01
Choline	-2.00E-01	4.50E-01	4.20E-01	9.70E-01	-2.90E-01	8.60E-01	3.30E-01	8.00E-01	2.50E-01	7.10E-01	4.80E-01	1.16E+00
Citramalic acid	-4.00E-02	2.00E-02	4.50E-01	1.11E+00	-5.40E-01	1.57E+00	-2.00E-02	1.00E-02	1.80E-01	5.80E-01	4.70E-01	1.20E+00
Citric acid	-1.10E-01	2.30E-01	5.70E-01	1.28E+00	-5.10E-01	1.43E+00	1.50E-01	2.90E-01	4.10E-01	1.06E+00	5.40E-01	1.32E+00
Citrulline	2.80E-01	7.30E-01	5.60E-01	1.27E+00	5.60E-01	1.62E+00	7.50E-01	1.80E+00	4.80E-01	1.52E+00	3.60E-01	7.60E-01
Creatine	-8.20E-01	2.14E+00	4.50E-01	1.00E+00	-7.90E-01	2.29E+00	4.20E-01	1.01E+00	4.10E-01	1.06E+00	7.90E-01	1.87E+00
Creatinine	-2.30E-01	5.30E-01	6.00E-01	1.38E+00	-2.80E-01	8.40E-01	5.30E-01	1.30E+00	3.00E-01	8.60E-01	6.20E-01	1.52E+00
Cysteine	4.00E-02	9.00E-02	-1.10E-01	1.90E-01	-2.50E-01	7.80E-01	-3.20E-01	8.00E-01	-1.90E-01	5.60E-01	-1.60E-01	3.50E-01
Cytidine	-2.70E-01	8.30E-01	-7.20E-01	1.65E+00	-7.00E-02	2.20E-01	-6.10E-01	1.51E+00	-7.40E-01	2.10E+00	-5.30E-01	1.28E+00
Cytidine monophosphate	-3.00E-02	9.00E-02	4.50E-01	9.90E-01	4.00E-02	2.40E-01	5.80E-01	1.45E+00	3.30E-01	1.12E+00	4.50E-01	1.06E+00
Cytosine	-2.20E-01	6.80E-01	-7.00E-01	1.60E+00	-5.00E-02	1.40E-01	-6.10E-01	1.49E+00	-7.20E-01	2.06E+00	-5.60E-01	1.34E+00
D-Erythrose	6.10E-01	1.60E+00	-1.30E-01	2.60E-01	5.30E-01	1.60E+00	-2.40E-01	5.70E-01	-2.30E-01	7.50E-01	-4.40E-01	9.60E-01
D-Fructose	-6.40E-01	1.70E+00	-2.00E-01	5.00E-01	-3.60E-01	1.08E+00	1.30E-01	2.50E-01	2.50E-01	8.40E-01	5.00E-01	1.11E+00
D-Fucitol	2.60E-01	6.90E-01	5.80E-01	1.29E+00	-1.10E-01	3.60E-01	4.20E-01	9.30E-01	4.10E-01	1.26E+00	5.20E-01	1.17E+00
D-Glucose	4.00E-02	1.10E-01	2.00E-01	5.10E-01	1.00E-02	3.00E-02	1.20E-01	2.50E-01	1.50E-01	3.50E-01	2.30E-01	5.10E-01
Dimethylamine	6.90E-01	1.92E+00	7.70E-01	1.75E+00	5.90E-01	1.70E+00	7.00E-01	1.68E+00	2.20E-01	6.60E-01	3.30E-01	8.00E-01
D-Lyxose	-2.10E-01	5.90E-01	3.80E-01	8.10E-01	0.00E+00	2.00E-02	4.40E-01	9.90E-01	3.80E-01	1.09E+00	4.30E-01	9.60E-01
D-Mannitol	-1.30E-01	3.50E-01	4.00E-02	1.10E-01	-3.70E-01	1.03E+00	-1.40E-01	4.30E-01	8.00E-02	7.00E-02	1.10E-01	3.10E-01
Docosahexaenoic acid	-2.40E-01	7.10E-01	5.30E-01	1.15E+00	-3.50E-01	1.04E+00	6.00E-01	1.43E+00	5.50E-01	1.62E+00	6.90E-01	1.56E+00
Docosapentaenoic acid	-6.50E-01	1.77E+00	3.10E-01	6.60E-01	-6.50E-01	1.86E+00	5.40E-01	1.31E+00	7.60E-01	2.17E+00	8.30E-01	1.90E+00
Docosenamide	-4.80E-01	1.32E+00	-2.30E-01	5.80E-01	-1.10E-01	4.00E-01	1.70E-01	4.10E-01	-1.00E-01	2.20E-01	3.10E-01	7.90E-01
Eicosapentaenoic acid	-7.20E-01	1.90E+00	-5.00E-02	1.60E-01	-4.80E-01	1.36E+00	3.00E-01	7.70E-01	6.80E-01	1.96E+00	7.20E-01	1.63E+00

Eicosatrienoic acid	-6.20E-01	1.65E+00	2.40E-01	5.10E-01	-5.40E-01	1.53E+00	4.10E-01	1.00E+00	6.70E-01	1.97E+00	8.30E-01	1.89E+00
Ethanolamine	2.00E-02	1.00E-02	1.30E-01	3.10E-01	-6.00E-02	1.80E-01	5.00E-02	9.00E-02	2.60E-01	9.30E-01	1.50E-01	3.50E-01
Fumaric acid	3.00E-02	1.00E-01	4.70E-01	1.09E+00	9.00E-02	3.10E-01	5.20E-01	1.26E+00	2.90E-01	9.20E-01	3.60E-01	9.20E-01
Galactose	-5.20E-01	1.42E+00	-1.30E-01	3.10E-01	-2.40E-01	7.40E-01	2.30E-01	5.30E-01	2.00E-02	2.60E-01	5.20E-01	1.16E+00
Glutamic acid	-5.20E-01	1.26E+00	-6.60E-01	1.49E+00	-6.30E-01	1.69E+00	-7.60E-01	1.86E+00	-6.10E-01	1.86E+00	-2.20E-01	4.90E-01
Glutamine	-5.70E-01	1.47E+00	5.30E-01	1.20E+00	-7.20E-01	2.12E+00	3.90E-01	9.20E-01	3.60E-01	9.40E-01	7.30E-01	1.72E+00
Glutaric acid	2.70E-01	7.30E-01	-1.90E-01	4.40E-01	1.00E-01	2.90E-01	-1.40E-01	4.50E-01	-4.10E-01	1.18E+00	-4.50E-01	1.04E+00
Glyceric acid	-3.80E-01	1.05E+00	5.40E-01	1.17E+00	-6.00E-01	1.75E+00	4.70E-01	1.10E+00	3.30E-01	1.05E+00	6.60E-01	1.45E+00
Glycerol	1.10E-01	3.70E-01	6.30E-01	1.42E+00	1.90E-01	5.30E-01	6.20E-01	1.44E+00	5.80E-01	1.81E+00	5.50E-01	1.27E+00
Glycine	-5.40E-01	1.37E+00	5.90E-01	1.34E+00	-6.10E-01	1.76E+00	5.20E-01	1.24E+00	5.10E-01	1.35E+00	7.30E-01	1.73E+00
Glycolic acid	2.80E-01	7.20E-01	6.30E-01	1.39E+00	-3.00E-02	3.00E-02	5.60E-01	1.33E+00	4.70E-01	1.44E+00	5.40E-01	1.20E+00
Guanosine	-5.10E-01	1.27E+00	-6.60E-01	1.51E+00	-2.10E-01	5.10E-01	-3.00E-01	7.70E-01	-4.70E-01	1.33E+00	-1.00E-01	2.10E-01
Histamine	2.30E-01	6.70E-01	6.80E-01	1.58E+00	-2.40E-01	7.20E-01	5.20E-01	1.25E+00	4.20E-01	1.18E+00	6.20E-01	1.48E+00
Histidine	-7.00E-02	2.30E-01	6.10E-01	1.37E+00	3.30E-01	9.20E-01	7.60E-01	1.78E+00	6.00E-01	1.75E+00	6.50E-01	1.43E+00
Homovallinic acid	3.10E-01	9.20E-01	5.00E-01	1.14E+00	-2.70E-01	7.10E-01	-1.10E-01	3.40E-01	-2.60E-01	8.50E-01	1.60E-01	3.90E-01
Hypoxanthine	-4.00E-01	1.00E+00	3.90E-01	9.00E-01	-4.90E-01	1.44E+00	3.10E-01	7.50E-01	2.60E-01	7.30E-01	5.50E-01	1.32E+00
Inosine	-6.90E-01	1.80E+00	-8.90E-01	1.99E+00	-4.00E-01	1.10E+00	-7.80E-01	1.88E+00	-7.70E-01	2.22E+00	-6.40E-01	1.42E+00
Isoleucine	-3.70E-01	9.00E-01	5.90E-01	1.36E+00	-4.30E-01	1.26E+00	5.80E-01	1.39E+00	4.30E-01	1.19E+00	7.20E-01	1.69E+00
Lactic acid	3.40E-01	8.80E-01	7.80E-01	1.77E+00	1.70E-01	6.00E-01	7.50E-01	1.81E+00	4.60E-01	1.40E+00	4.90E-01	1.15E+00
Leucine	-3.70E-01	9.10E-01	5.90E-01	1.35E+00	-4.20E-01	1.23E+00	5.90E-01	1.41E+00	3.80E-01	1.05E+00	7.20E-01	1.69E+00
LPC 14:0 sn-1	-1.70E-01	4.50E-01	-4.30E-01	9.90E-01	-1.10E-01	3.50E-01	-4.90E-01	1.25E+00	-2.60E-01	8.60E-01	-1.90E-01	4.60E-01
LPC 14:0 sn-2	-1.30E-01	3.50E-01	-4.20E-01	1.01E+00	-1.10E-01	3.00E-01	-4.90E-01	1.28E+00	-8.00E-02	4.80E-01	-2.80E-01	6.90E-01
LPC 16:0 sn-1	-1.20E-01	2.70E-01	-3.30E-01	7.70E-01	1.20E-01	3.40E-01	-1.10E-01	2.80E-01	-2.80E-01	6.80E-01	-2.30E-01	5.10E-01
LPC 16:0 sn-2	4.00E-02	3.00E-02	-6.00E-02	1.00E-01	-2.60E-01	7.60E-01	-4.40E-01	1.10E+00	2.00E-02	5.00E-02	-5.00E-02	1.10E-01
LPC 16:1 sn-1	-2.50E-01	7.10E-01	1.00E-01	2.00E-01	-2.00E-01	5.90E-01	2.60E-01	6.30E-01	2.90E-01	7.30E-01	3.40E-01	7.80E-01
LPC 16:1 sn-2	-3.20E-01	9.20E-01	9.00E-02	1.80E-01	-3.40E-01	1.03E+00	2.30E-01	5.70E-01	1.70E-01	4.50E-01	4.60E-01	1.10E+00
LPC 18:0 sn-1	8.00E-02	1.70E-01	-2.80E-01	5.90E-01	2.90E-01	8.40E-01	-7.00E-02	1.90E-01	3.00E-02	6.00E-02	-3.10E-01	6.90E-01
LPC 18:0 sn-2	-8.00E-02	2.50E-01	-3.00E-02	8.00E-02	-1.30E-01	3.90E-01	-7.00E-02	2.30E-01	-6.00E-02	1.80E-01	7.00E-02	1.70E-01
LPC 18:1 sn-1	-2.00E-02	4.00E-02	-1.20E-01	2.50E-01	3.00E-02	5.00E-02	-6.00E-02	1.90E-01	-4.00E-02	5.00E-02	-1.20E-01	2.00E-01
LPC 18:1 sn-2	-1.40E-01	3.90E-01	-1.80E-01	4.00E-01	-1.60E-01	4.80E-01	-2.10E-01	5.70E-01	-6.00E-02	2.40E-01	1.00E-02	3.00E-02
LPC 18:2 sn-1	-1.10E-01	3.30E-01	-1.10E-01	2.90E-01	-1.40E-01	4.30E-01	-1.60E-01	4.90E-01	-1.40E-01	4.00E-01	4.00E-02	9.00E-02
LPC 18:2 sn-2	-5.00E-02	2.10E-01	-1.20E-01	3.30E-01	-2.40E-01	6.90E-01	-3.50E-01	9.10E-01	-1.60E-01	4.80E-01	-6.00E-02	1.50E-01
LPC 20:0 sn-1	-8.00E-02	2.60E-01	-1.60E-01	3.80E-01	1.20E-01	3.40E-01	1.10E-01	2.10E-01	2.30E-01	6.50E-01	-5.00E-02	1.30E-01
LPC 20:0 sn-2	-6.00E-02	2.00E-01	-2.50E-01	5.90E-01	1.00E-02	1.10E-01	-1.30E-01	4.20E-01	1.80E-01	3.40E-01	-2.00E-01	5.30E-01

LPC 20:1 sn-1	-1.00E-02	3.00E-02	5.00E-02	8.00E-02	5.00E-02	1.40E-01	1.60E-01	3.00E-01	9.00E-02	2.20E-01	4.00E-02	9.00E-02
LPC 20:1 sn-2	1.00E-02	3.00E-02	2.30E-01	5.00E-01	7.00E-02	2.00E-01	3.90E-01	8.40E-01	2.30E-01	5.80E-01	2.60E-01	6.00E-01
LPC 20:4 sn-1	-2.00E-01	5.20E-01	-3.40E-01	7.80E-01	-1.60E-01	4.60E-01	-3.60E-01	9.20E-01	-3.40E-01	9.40E-01	-5.00E-02	1.30E-01
LPC 20:4 sn-2	-2.10E-01	6.00E-01	-4.10E-01	9.30E-01	-2.70E-01	7.70E-01	-5.20E-01	1.28E+00	-3.40E-01	9.80E-01	-2.00E-01	4.50E-01
LPC 20:5 sn-1	-5.10E-01	1.37E+00	-2.10E-01	5.30E-01	-3.00E-01	8.30E-01	2.30E-01	5.40E-01	5.10E-01	1.45E+00	4.30E-01	9.60E-01
LPC 20:5 sn-2	-6.70E-01	1.82E+00	-3.30E-01	7.60E-01	-3.60E-01	1.02E+00	1.90E-01	5.00E-01	4.50E-01	1.32E+00	6.00E-01	1.41E+00
LPC 22:4 sn-1	-1.60E-01	4.30E-01	-2.40E-01	5.50E-01	-4.00E-02	1.50E-01	-1.10E-01	3.20E-01	-2.70E-01	7.10E-01	-3.00E-02	8.00E-02
LPC 22:4 sn-2	-2.00E-02	1.10E-01	-2.00E-01	4.60E-01	-1.00E-02	7.00E-02	-2.30E-01	6.10E-01	-2.70E-01	7.20E-01	-1.60E-01	3.70E-01
LPC 22:6 sn-1	-2.10E-01	5.80E-01	-1.90E-01	4.40E-01	4.00E-02	1.20E-01	2.60E-01	5.90E-01	1.50E-01	3.40E-01	1.10E-01	2.70E-01
LPC 22:6 sn-2	-1.80E-01	5.10E-01	-1.60E-01	3.80E-01	-2.50E-01	7.30E-01	-2.40E-01	6.40E-01	-4.00E-02	2.00E-01	8.00E-02	1.80E-01
LPE 16:0 sn-1	-1.70E-01	4.20E-01	-1.70E-01	3.90E-01	-1.90E-01	5.50E-01	-1.90E-01	5.20E-01	-1.10E-01	4.00E-01	1.00E-02	2.00E-02
LPE 16:0 sn-2	5.00E-02	1.10E-01	-1.40E-01	2.90E-01	1.00E-02	5.00E-02	-1.40E-01	3.80E-01	-1.90E-01	6.50E-01	-1.50E-01	3.60E-01
LPE 16:1 sn-1	-2.40E-01	6.60E-01	-9.00E-02	2.10E-01	-1.90E-01	5.70E-01	1.00E-02	4.00E-02	3.00E-02	1.00E-02	1.60E-01	3.70E-01
LPE 16:1 sn-2	8.00E-02	1.80E-01	3.80E-01	8.60E-01	-9.00E-02	2.30E-01	3.30E-01	7.50E-01	2.10E-01	4.70E-01	3.40E-01	7.90E-01
LPE 18:0 sn-1	-1.00E-02	5.00E-02	-3.40E-01	7.60E-01	-4.00E-02	1.10E-01	-3.70E-01	9.80E-01	-3.80E-01	1.19E+00	-2.90E-01	6.70E-01
LPE 18:0 sn-2	-1.40E-01	3.60E-01	-3.20E-01	7.00E-01	-1.30E-01	3.90E-01	-3.00E-01	7.70E-01	-2.70E-01	8.60E-01	-1.40E-01	3.30E-01
LPE 18:1 sn-1	-1.50E-01	4.20E-01	-1.00E-02	1.00E-02	-1.00E-01	3.30E-01	8.00E-02	1.10E-01	6.00E-02	1.00E-01	1.30E-01	2.90E-01
LPE 18:1 sn-2	2.50E-01	6.20E-01	2.40E-01	5.60E-01	2.50E-01	7.50E-01	2.70E-01	6.20E-01	6.00E-02	7.00E-02	1.10E-01	2.50E-01
LPE 18:2 sn-1	-1.00E-01	3.00E-01	-2.00E-01	4.50E-01	-1.70E-01	5.30E-01	-2.50E-01	6.90E-01	-2.20E-01	7.40E-01	-6.00E-02	1.40E-01
LPE 18:2 sn-2	3.00E-02	4.00E-02	-9.00E-02	1.70E-01	-2.60E-01	7.40E-01	-2.60E-01	6.70E-01	-2.60E-01	8.10E-01	-9.00E-02	2.10E-01
LPE 20:1 sn-1	-1.20E-01	3.40E-01	1.00E-01	2.50E-01	-1.10E-01	3.70E-01	1.50E-01	3.00E-01	1.80E-01	4.80E-01	2.00E-01	4.80E-01
LPE 20:1 sn-2	2.40E-01	6.20E-01	2.20E-01	5.10E-01	2.90E-01	8.60E-01	2.90E-01	6.40E-01	8.00E-02	1.70E-01	8.00E-02	2.00E-01
LPE 20:2 sn-1	3.20E-01	8.40E-01	2.20E-01	5.30E-01	2.90E-01	8.70E-01	2.30E-01	5.40E-01	6.00E-02	8.00E-02	3.00E-02	6.00E-02
LPE 20:2 sn-2	2.60E-01	7.00E-01	-4.70E-01	1.04E+00	3.00E-01	9.10E-01	-4.50E-01	1.12E+00	-3.50E-01	1.12E+00	-5.70E-01	1.36E+00
LPE 20:4 sn-1	-1.50E-01	4.00E-01	-3.40E-01	7.40E-01	-1.70E-01	5.10E-01	-3.30E-01	8.60E-01	-3.20E-01	1.00E+00	-1.90E-01	4.60E-01
LPE 20:4 sn-2	8.00E-02	1.90E-01	-1.30E-01	2.60E-01	-1.00E-02	2.00E-02	-1.70E-01	4.30E-01	-2.40E-01	7.40E-01	-1.60E-01	3.80E-01
LPE 22:4 sn-1	-2.20E-01	6.20E-01	-3.00E-01	6.40E-01	-2.00E-01	6.20E-01	-2.40E-01	6.40E-01	-1.90E-01	5.60E-01	-4.00E-02	7.00E-02
LPE 22:4 sn-2	1.00E-01	2.30E-01	-2.00E-02	1.00E-02	1.00E-01	3.10E-01	2.00E-02	1.00E-02	-1.20E-01	3.30E-01	-7.00E-02	1.60E-01
LPE 22:6 sn-1	-3.30E-01	8.30E-01	-1.80E-01	3.50E-01	-9.00E-02	2.10E-01	1.00E-01	2.60E-01	-9.00E-02	2.50E-01	1.50E-01	3.30E-01
LPE 22:6 sn-2	1.50E-01	4.70E-01	6.00E-02	1.70E-01	-2.20E-01	6.00E-01	-1.20E-01	3.30E-01	-1.00E-01	3.80E-01	-1.00E-02	5.00E-02
LPE P-16:0	-1.80E-01	4.80E-01	-4.30E-01	9.50E-01	-1.60E-01	4.80E-01	-3.90E-01	1.00E+00	-3.10E-01	9.60E-01	-2.00E-01	4.80E-01
LPE P-18:0	-1.60E-01	4.30E-01	-4.10E-01	9.00E-01	-1.40E-01	4.30E-01	-3.70E-01	9.60E-01	-2.90E-01	9.10E-01	-2.10E-01	5.00E-01
LPE P-18:1	-1.10E-01	2.70E-01	7.00E-02	1.70E-01	-9.00E-02	2.80E-01	1.20E-01	2.10E-01	1.00E-02	3.00E-02	1.40E-01	3.20E-01
LPE P-20:0	-1.80E-01	5.00E-01	-4.60E-01	1.03E+00	-1.10E-01	3.30E-01	-3.90E-01	1.01E+00	-2.80E-01	9.30E-01	-2.80E-01	6.80E-01

LPE P-20:1	-4.00E-02	1.10E-01	5.00E-02	1.20E-01	1.00E-02	2.00E-02	1.30E-01	2.50E-01	6.00E-02	1.10E-01	8.00E-02	1.80E-01
LPE P-22:1	-1.00E-02	1.00E-02	-1.40E-01	3.10E-01	1.50E-01	4.20E-01	1.00E-02	6.00E-02	-1.00E-02	1.10E-01	-1.40E-01	3.30E-01
LPG 16:0 sn-1	-1.00E-02	3.00E-02	3.90E-01	8.50E-01	-4.00E-02	1.10E-01	4.30E-01	1.00E+00	1.70E-01	4.50E-01	3.90E-01	9.10E-01
LPG 16:0 sn-2	3.00E-02	1.20E-01	4.10E-01	9.00E-01	-1.40E-01	3.90E-01	4.00E-01	9.30E-01	2.20E-01	6.40E-01	4.50E-01	1.03E+00
LPG 18:0 sn-1	-2.00E-02	2.00E-02	4.10E-01	9.00E-01	-5.00E-02	1.50E-01	4.50E-01	1.10E+00	1.40E-01	4.60E-01	4.10E-01	9.80E-01
LPG 18:0 sn-2	1.40E-01	4.80E-01	4.50E-01	9.90E-01	-3.10E-01	9.00E-01	1.70E-01	4.00E-01	1.30E-01	3.90E-01	3.20E-01	7.90E-01
LPG 18:1 sn-1	-1.80E-01	4.60E-01	5.30E-01	1.17E+00	-1.70E-01	5.20E-01	6.90E-01	1.61E+00	8.00E-02	2.40E-01	6.30E-01	1.47E+00
LPG 18:1 sn-2	-1.30E-01	3.80E-01	4.50E-01	1.00E+00	-1.90E-01	5.90E-01	4.80E-01	1.13E+00	2.70E-01	8.00E-01	5.10E-01	1.19E+00
LPG 20:4 sn-1	-2.90E-01	7.50E-01	2.40E-01	5.00E-01	-2.30E-01	6.50E-01	4.90E-01	1.12E+00	1.00E-02	6.00E-02	5.30E-01	1.19E+00
LPG 20:4 sn-2	-1.40E-01	3.90E-01	3.00E-01	6.70E-01	-3.20E-01	1.00E+00	1.80E-01	4.70E-01	-3.00E-02	4.00E-02	4.40E-01	9.90E-01
LPG 22:6 sn-1	-2.30E-01	6.50E-01	6.80E-01	1.51E+00	-3.00E-01	9.10E-01	7.50E-01	1.80E+00	1.70E-01	5.00E-01	7.50E-01	1.76E+00
LPG 22:6 sn-2	-2.30E-01	7.30E-01	6.50E-01	1.46E+00	-1.70E-01	5.40E-01	6.90E-01	1.70E+00	2.30E-01	7.20E-01	7.30E-01	1.73E+00
LPI 16:0 sn-1	6.60E-01	1.78E+00	5.30E-01	1.20E+00	4.00E-01	1.19E+00	1.70E-01	4.10E-01	-3.00E-02	2.40E-01	-3.70E-01	8.30E-01
LPI 16:0 sn-2	4.20E-01	1.14E+00	3.70E-01	8.50E-01	3.30E-01	1.02E+00	2.90E-01	6.90E-01	0.00E+00	1.40E-01	-9.00E-02	1.80E-01
LPI 18:0 sn-1	5.50E-01	1.48E+00	1.40E-01	3.40E-01	3.30E-01	9.90E-01	-9.00E-02	2.20E-01	-1.70E-01	6.10E-01	-4.00E-01	9.30E-01
LPI 18:0 sn-2	4.70E-01	1.26E+00	1.30E-01	3.10E-01	2.90E-01	8.60E-01	-7.00E-02	1.80E-01	-1.50E-01	5.30E-01	-3.20E-01	7.40E-01
LPI 18:1 sn-1	4.10E-01	1.10E+00	2.60E-01	6.00E-01	2.00E-01	6.20E-01	1.00E-02	2.00E-02	-1.50E-01	5.50E-01	-2.70E-01	5.90E-01
LPI 18:1 sn-2	4.20E-01	1.12E+00	2.80E-01	6.50E-01	1.90E-01	5.90E-01	5.00E-02	9.00E-02	-1.00E-02	1.80E-01	-1.90E-01	4.20E-01
LPI 20:4 sn-1	5.00E-02	1.90E-01	-1.00E-01	2.00E-01	-1.00E-02	4.00E-02	-2.30E-01	5.30E-01	-2.40E-01	7.70E-01	-1.70E-01	3.50E-01
LPI 20:4 sn-2	3.80E-01	1.01E+00	-2.00E-02	0.00E+00	1.20E-01	3.80E-01	-2.00E-01	4.90E-01	-1.90E-01	6.50E-01	-3.60E-01	7.90E-01
LPI 22:6 sn-1	3.00E-01	8.20E-01	5.80E-01	1.30E+00	2.00E-01	6.10E-01	5.20E-01	1.22E+00	2.40E-01	5.40E-01	1.90E-01	4.70E-01
LPI 22:6 sn-2	5.70E-01	1.52E+00	4.80E-01	1.11E+00	5.00E-01	1.45E+00	4.30E-01	9.90E-01	2.00E-01	4.20E-01	-6.00E-02	1.00E-01
LPS 18:0 sn-1	1.10E-01	3.00E-01	2.00E-02	4.00E-02	1.40E-01	4.40E-01	7.00E-02	1.20E-01	-8.00E-02	2.60E-01	-7.00E-02	1.60E-01
LPS 18:0 sn-2	-3.00E-02	4.00E-02	1.00E-02	0.00E+00	5.00E-02	1.80E-01	1.30E-01	2.50E-01	-5.00E-02	1.70E-01	2.00E-02	3.00E-02
LPS 18:1 sn-1	1.60E-01	4.40E-01	2.70E-01	5.80E-01	2.10E-01	6.00E-01	3.30E-01	7.20E-01	6.00E-02	7.00E-02	6.00E-02	1.30E-01
LPS 18:1 sn-2	4.90E-01	1.31E+00	3.50E-01	8.20E-01	4.30E-01	1.26E+00	3.50E-01	7.90E-01	1.30E-01	3.00E-01	4.00E-02	8.00E-02
Lysine	-5.00E-02	1.90E-01	5.90E-01	1.31E+00	2.00E-02	7.00E-02	5.60E-01	1.27E+00	6.30E-01	1.97E+00	6.20E-01	1.43E+00
Malic acid	-9.00E-02	1.70E-01	6.00E-01	1.40E+00	-9.00E-02	1.70E-01	6.80E-01	1.64E+00	3.50E-01	1.19E+00	5.50E-01	1.35E+00
Malonic acid	1.90E-01	5.50E-01	6.50E-01	1.46E+00	-3.10E-01	8.00E-01	4.80E-01	1.14E+00	5.40E-01	1.62E+00	6.20E-01	1.42E+00
Methionine	-3.70E-01	9.00E-01	6.00E-01	1.37E+00	-4.40E-01	1.26E+00	6.10E-01	1.47E+00	4.00E-01	1.11E+00	7.30E-01	1.71E+00
Methylamine	-6.30E-01	1.60E+00	5.00E-01	1.17E+00	-5.90E-01	1.70E+00	5.10E-01	1.26E+00	2.90E-01	8.90E-01	7.50E-01	1.76E+00
MG 14:0 (1)	-8.00E-01	2.15E+00	-8.00E-01	1.82E+00	-8.40E-01	2.42E+00	-8.10E-01	1.97E+00	-5.20E-01	1.65E+00	-4.80E-01	1.04E+00
MG 16:0 (1)	-8.00E-02	2.00E-01	4.40E-01	9.60E-01	-1.90E-01	5.20E-01	4.60E-01	1.09E+00	4.20E-01	1.04E+00	5.60E-01	1.34E+00
MG 16:0 (2)	-8.00E-02	2.30E-01	3.50E-01	7.60E-01	-2.00E-01	6.00E-01	3.40E-01	7.90E-01	4.00E-01	9.80E-01	4.70E-01	1.13E+00

MG 18:0 (1)	2.50E-01	6.80E-01	3.40E-01	7.50E-01	-3.00E-02	8.00E-02	6.00E-02	1.50E-01	1.30E-01	1.20E-01	1.00E-01	2.60E-01
MG 18:0 (2)	-3.40E-01	1.03E+00	-6.10E-01	1.36E+00	-5.40E-01	1.62E+00	-6.60E-01	1.63E+00	-3.60E-01	1.20E+00	-3.20E-01	6.70E-01
MG 18:1 (1)	-6.90E-01	1.87E+00	-7.20E-01	1.61E+00	-6.70E-01	1.97E+00	-7.10E-01	1.71E+00	-3.80E-01	1.25E+00	-3.80E-01	7.70E-01
MG 18:1 (2)	-3.00E-02	6.00E-02	4.00E-01	9.30E-01	2.00E-02	4.00E-02	4.30E-01	9.90E-01	1.60E-01	4.60E-01	3.10E-01	7.40E-01
MG 18:2 (2)	-7.00E-02	2.40E-01	0.00E+00	3.00E-02	-2.80E-01	8.50E-01	-2.40E-01	6.80E-01	-1.70E-01	5.30E-01	7.00E-02	2.30E-01
MG 20:4 (1)	-3.10E-01	8.10E-01	-1.40E-01	3.30E-01	-1.70E-01	4.80E-01	4.00E-02	2.00E-02	-1.80E-01	4.40E-01	2.40E-01	5.10E-01
MG 20:4 (2)	-4.00E-01	1.05E+00	-2.10E-01	4.60E-01	-2.00E-01	6.00E-01	5.00E-02	9.00E-02	-2.00E-01	5.00E-01	3.00E-01	6.40E-01
MG 22:6 (1)	-2.80E-01	8.20E-01	5.00E-02	1.20E-01	-3.10E-01	9.10E-01	7.00E-02	1.50E-01	-9.00E-02	2.30E-01	3.80E-01	8.20E-01
MG 22:6 (2)	-4.20E-01	1.16E+00	4.00E-02	1.00E-01	-4.10E-01	1.23E+00	1.10E-01	2.10E-01	0.00E+00	5.00E-02	4.70E-01	1.05E+00
Monoisoamylamine	6.80E-01	1.88E+00	6.10E-01	1.44E+00	2.00E-01	8.30E-01	3.00E-01	7.70E-01	3.60E-01	9.00E-01	1.80E-01	4.40E-01
Myo-Inositol	-2.70E-01	6.80E-01	-3.00E-02	6.00E-02	1.30E-01	3.90E-01	2.90E-01	6.20E-01	2.70E-01	8.60E-01	2.90E-01	7.10E-01
NAA (N-acetylaspartate)	-2.50E-01	5.70E-01	-7.60E-01	1.65E+00	-3.50E-01	9.70E-01	-7.70E-01	1.81E+00	-7.80E-01	2.31E+00	-7.30E-01	1.61E+00
NAAG (N-acetyl-aspartyl-glutamate)	-2.00E-01	6.10E-01	-3.00E-02	7.00E-02	5.10E-01	1.46E+00	5.60E-01	1.29E+00	-8.00E-02	1.80E-01	2.20E-01	4.50E-01
N-Acetyl-L-glutamic acid	-5.50E-01	1.49E+00	-2.50E-01	4.90E-01	-3.50E-01	1.05E+00	-6.00E-02	2.30E-01	3.30E-01	8.30E-01	4.90E-01	1.13E+00
N-Acetylneuraminic Acid	0.00E+00	1.10E-01	4.60E-01	9.80E-01	2.50E-01	6.00E-01	6.80E-01	1.60E+00	2.30E-01	7.20E-01	5.50E-01	1.15E+00
Nervonic acid	-1.00E-01	3.00E-01	-3.00E-01	6.50E-01	-5.60E-01	1.66E+00	-6.10E-01	1.55E+00	-2.60E-01	8.60E-01	-1.90E-01	3.60E-01
Nicotinamide	-7.80E-01	2.08E+00	-6.10E-01	1.34E+00	-3.20E-01	8.70E-01	-2.00E-01	4.10E-01	-3.90E-01	1.05E+00	1.60E-01	3.40E-01
Nicotinic acid	-4.80E-01	1.22E+00	6.00E-01	1.32E+00	-2.10E-01	6.80E-01	6.60E-01	1.59E+00	7.50E-01	2.13E+00	7.50E-01	1.64E+00
N-oleoyl taurine	-1.20E-01	3.20E-01	4.70E-01	1.02E+00	-4.30E-01	1.21E+00	2.40E-01	6.20E-01	5.90E-01	1.69E+00	6.10E-01	1.39E+00
Nonanoic acid	-3.50E-01	1.03E+00	-5.60E-01	1.24E+00	-4.00E-01	1.20E+00	-5.00E-01	1.29E+00	-3.40E-01	1.08E+00	-3.30E-01	7.00E-01
Octadecadienoic acid	-4.60E-01	1.27E+00	-1.40E-01	3.00E-01	-5.20E-01	1.56E+00	-2.40E-01	6.60E-01	1.30E-01	1.60E-01	4.70E-01	1.08E+00
Oleic acid	-4.90E-01	1.32E+00	2.60E-01	5.70E-01	-2.90E-01	8.20E-01	5.80E-01	1.39E+00	4.90E-01	1.50E+00	6.80E-01	1.56E+00
Orthophosphate	2.20E-01	6.30E-01	2.40E-01	5.30E-01	4.00E-02	1.70E-01	2.00E-02	0.00E+00	1.10E-01	2.90E-01	-2.00E-02	1.30E-01
Palmitic acid	-6.90E-01	1.83E+00	3.70E-01	7.90E-01	-5.60E-01	1.60E+00	5.40E-01	1.26E+00	6.50E-01	1.84E+00	8.30E-01	1.88E+00
Palmitoylcarnitine	4.60E-01	1.20E+00	7.20E-01	1.61E+00	1.90E-01	6.10E-01	6.00E-01	1.46E+00	1.10E-01	3.60E-01	3.50E-01	8.90E-01
Pantothenate	1.60E-01	3.60E-01	7.00E-02	1.30E-01	5.30E-01	1.57E+00	4.10E-01	9.60E-01	-3.10E-01	8.70E-01	-5.00E-02	2.20E-01
Phenylalanine	-4.40E-01	1.10E+00	5.50E-01	1.27E+00	-4.80E-01	1.41E+00	5.50E-01	1.33E+00	4.20E-01	1.14E+00	7.30E-01	1.72E+00
Phosphocholine	-6.10E-01	1.65E+00	-6.50E-01	1.43E+00	-3.90E-01	1.10E+00	-4.50E-01	1.05E+00	-6.50E-01	1.87E+00	-1.10E-01	3.10E-01
Phosphorylethanolamine	-1.90E-01	5.00E-01	-5.10E-01	1.11E+00	-5.20E-01	1.54E+00	-6.70E-01	1.59E+00	-6.90E-01	2.08E+00	-4.20E-01	9.70E-01
Pimelic acid	-5.30E-01	1.44E+00	-4.20E-01	9.60E-01	-4.30E-01	1.24E+00	-3.20E-01	8.50E-01	-1.30E-01	6.10E-01	5.00E-02	4.00E-02
Pipecolic acid	2.80E-01	7.80E-01	6.70E-01	1.48E+00	5.70E-01	1.62E+00	8.80E-01	2.08E+00	5.00E-01	1.52E+00	5.10E-01	1.11E+00
Proline	-2.00E-02	8.00E-02	5.20E-01	1.14E+00	3.30E-01	9.30E-01	7.40E-01	1.73E+00	5.20E-01	1.57E+00	5.30E-01	1.12E+00
Putrescine	7.00E-02	2.90E-01	-5.10E-01	1.11E+00	-4.00E-01	1.06E+00	-7.60E-01	1.80E+00	-5.70E-01	1.62E+00	-5.10E-01	1.14E+00
Pyridoxine	-3.50E-01	9.50E-01	4.80E-01	1.08E+00	-4.80E-01	1.43E+00	4.60E-01	1.09E+00	3.90E-01	1.23E+00	7.00E-01	1.59E+00

Pyroglutamic acid	-4.40E-01	1.08E+00	4.90E-01	1.11E+00	-5.70E-01	1.63E+00	4.00E-01	9.70E-01	4.40E-01	1.11E+00	6.80E-01	1.64E+00
Pyruvic acid	5.30E-01	1.37E+00	5.20E-01	1.21E+00	8.00E-02	3.40E-01	2.20E-01	5.60E-01	1.70E-01	3.70E-01	9.00E-02	2.90E-01
Riboflavine	-5.60E-01	1.44E+00	5.60E-01	1.32E+00	-4.20E-01	1.22E+00	6.40E-01	1.59E+00	3.20E-01	9.60E-01	8.30E-01	1.99E+00
Ribose	3.70E-01	1.10E+00	7.20E-01	1.65E+00	2.10E-01	6.20E-01	6.60E-01	1.62E+00	3.80E-01	1.09E+00	6.00E-01	1.44E+00
S-Adenosyl-L-homocysteine	1.10E-01	2.20E-01	-5.30E-01	1.13E+00	2.90E-01	8.90E-01	-5.00E-01	1.15E+00	-5.00E-01	1.51E+00	-5.60E-01	1.22E+00
Serine	-4.10E-01	1.03E+00	5.80E-01	1.31E+00	-5.70E-01	1.64E+00	5.00E-01	1.19E+00	4.50E-01	1.18E+00	6.80E-01	1.60E+00
Stearic acid	-4.00E-02	1.90E-01	1.00E-01	2.90E-01	-3.80E-01	1.15E+00	-2.70E-01	7.10E-01	-9.00E-02	3.90E-01	1.90E-01	4.70E-01
Succinic acid	-1.70E-01	4.70E-01	-4.50E-01	1.03E+00	-1.50E-01	4.70E-01	-4.30E-01	1.13E+00	-4.30E-01	1.20E+00	-2.60E-01	7.30E-01
Tartaric acid	-7.00E-02	1.90E-01	3.60E-01	8.10E-01	-2.30E-01	6.00E-01	3.10E-01	7.30E-01	4.90E-01	1.37E+00	4.20E-01	1.02E+00
Taurine	-4.90E-01	1.22E+00	5.80E-01	1.32E+00	-6.30E-01	1.83E+00	4.90E-01	1.17E+00	4.00E-01	1.09E+00	7.40E-01	1.76E+00
Taurocholic acid	-1.80E-01	4.80E-01	-2.00E-02	9.00E-02	6.00E-02	1.50E-01	1.90E-01	4.30E-01	-3.00E-01	1.04E+00	1.70E-01	3.50E-01
Thiamine	-1.70E-01	3.90E-01	5.60E-01	1.29E+00	-1.40E-01	4.00E-01	5.50E-01	1.37E+00	3.20E-01	9.20E-01	6.40E-01	1.54E+00
Threonine	-5.80E-01	1.46E+00	5.90E-01	1.35E+00	-6.70E-01	1.94E+00	5.40E-01	1.29E+00	4.70E-01	1.26E+00	7.50E-01	1.76E+00
Thymidine	-4.20E-01	1.07E+00	1.50E-01	3.60E-01	-3.80E-01	1.12E+00	1.90E-01	4.50E-01	2.00E-01	5.50E-01	4.30E-01	1.03E+00
Thymine	-3.20E-01	7.90E-01	3.60E-01	8.40E-01	-2.60E-01	7.80E-01	3.70E-01	8.90E-01	2.80E-01	7.70E-01	5.30E-01	1.28E+00
Trimethylamine	-1.60E-01	3.10E-01	2.80E-01	6.60E-01	-1.00E-01	2.50E-01	3.50E-01	8.90E-01	4.20E-01	1.21E+00	4.00E-01	1.02E+00
Trimethylamine-n-oxide	6.90E-01	1.85E+00	3.00E-01	7.00E-01	7.80E-01	2.26E+00	3.80E-01	9.20E-01	-1.00E-02	3.00E-02	-5.50E-01	1.26E+00
Trimethyllysine N6,N6,N6	1.00E-01	1.60E-01	-9.00E-02	2.30E-01	7.00E-02	1.60E-01	-1.10E-01	2.40E-01	-5.10E-01	1.51E+00	-1.60E-01	3.40E-01
Tryptamine	1.10E-01	2.70E-01	4.70E-01	1.06E+00	1.00E-01	2.30E-01	4.70E-01	1.10E+00	2.90E-01	8.70E-01	4.80E-01	1.09E+00
Tryptophan	-5.10E-01	1.28E+00	5.30E-01	1.20E+00	-5.40E-01	1.59E+00	5.10E-01	1.23E+00	3.70E-01	1.02E+00	7.30E-01	1.73E+00
Tyrosine	-4.80E-01	1.21E+00	6.00E-01	1.37E+00	-6.10E-01	1.78E+00	5.60E-01	1.35E+00	4.60E-01	1.33E+00	7.70E-01	1.81E+00
Uracil	5.70E-01	1.44E+00	7.10E-01	1.58E+00	3.00E-01	8.20E-01	5.80E-01	1.31E+00	6.00E-01	1.74E+00	4.50E-01	1.03E+00
Uridine	-6.60E-01	1.76E+00	-8.40E-01	1.90E+00	-2.20E-01	5.70E-01	-6.80E-01	1.61E+00	-5.00E-01	1.52E+00	-7.10E-01	1.64E+00
Valine	3.40E-01	8.90E-01	6.30E-01	1.38E+00	3.40E-01	9.70E-01	6.00E-01	1.37E+00	5.60E-01	1.62E+00	3.60E-01	7.70E-01
Xanthine	-4.90E-01	1.24E+00	5.60E-01	1.29E+00	-5.90E-01	1.74E+00	4.70E-01	1.15E+00	3.20E-01	8.90E-01	7.50E-01	1.78E+00
γ-Aminobutyric acid	-4.20E-01	1.06E+00	6.30E-01	1.42E+00	-6.00E-01	1.76E+00	5.00E-01	1.19E+00	3.70E-01	1.01E+00	7.30E-01	1.72E+00

Table S4 (a and b). Statistically significant metabolites that met the criteria of *p* value ≤ 0.05 and VIP value ≥1.5 from univariate and multivariate statistics for pairwise comparisons of the studied groups. In bold are compounds in common (table a. comparisons 1v4, 2v4, 4v5 and table b. comparisons 1v5, 2v5, 3v5).

**a.**	**1v4**		**2v4**			**4v5**	
**Compounds**	***p***	**VIP**	**Compounds**	***p***	**VIP**	**Compounds**	***p***	**VIP**
3-Hydroxybutyric acid	4.55E-04	2.01	4-Hydroxybenzoic acid	4.89E-03	1.93	5-Methylthioadenosine	1.90E-04	1.72
3-Methyl-2-oxovaleric acid	6.29E-03	1.62	**Acetylcarnitine**	2.58E-04	2.13	**Acetylcarnitine**	3.74E-04	1.66
4-Hydroxybenzoic acid	4.13E-04	1.97	Alanine	1.96E-02	1.86	Alanine	1.89E-03	1.79
4-Hydroxyphenyllactic acid	1.08E-02	1.46	Arachidonoylcarnitine	7.64E-03	1.67	**Asparagine**	2.18E-02	1.65
5-Methylthioadenosine	3.91E-03	1.68	**Asparagine**	2.72E-02	1.96	Betaine	1.42E-03	1.56
**Acetylcarnitine**	1.83E-06	2.31	Citramalic acid	1.31E-02	1.57	**Creatine**	7.91E-04	1.87
Arachidonic acid	3.34E-03	2.04	Citrulline	1.14E-02	1.62	Creatinine	3.70E-03	1.52
Arachidonoylcarnitine	9.19E-05	1.98	**Creatine**	5.26E-04	2.29	**Docosahexaenoic acid**	1.38E-03	1.56
**Asparagine**	1.69E-02	1.58	D-Erythrose	8.76E-03	1.60	Docosapentaenoic acid	2.40E-05	1.90
Carnitine	4.22E-06	2.24	Dimethylamine	1.65E-02	1.70	Eicosapentaenoic acid	6.21E-04	1.63
**Creatine**	1.77E-04	2.14	**Docosapentaenoic acid**	3.18E-03	1.86	**Eicosatrienoic acid**	2.62E-05	1.89
D-Erythrose	3.56E-03	1.60	**Eicosatrienoic acid**	2.10E-02	1.53	**Glutamine**	6.58E-03	1.72
D-Fructose	3.70E-03	1.70	Glutamic acid	7.73E-03	1.69	Glyceric acid	4.89E-02	1.45
Dimethylamine	1.33E-03	1.92	**Glutamine**	1.14E-02	2.12	Glycine	4.42E-03	1.73
**Docosapentaenoic acid**	2.02E-03	1.77	Glyceric acid	8.88E-03	1.75	Histamine	8.56E-03	1.48
Eicosapentaenoic acid	6.91E-04	1.90	Glycine	3.18E-02	1.76	Isoleucine	9.57E-04	1.69
**Eicosatrienoic acid**	6.17E-03	1.65	**Methylamine**	7.73E-03	1.70	Leucine	5.18E-04	1.69
**Glutamine**	1.93E-02	1.47	MG 14:0 (1)	2.19E-05	2.42	LPG 18:1 sn-1	1.90E-03	1.47
Inosine	1.07E-03	1.80	MG 18:0 (2)	1.18E-02	1.62	LPG 22:6 sn-1	5.16E-05	1.76
LPC 20:5 sn-2	9.00E-04	1.82	MG 18:1 (1)	1.38E-03	1.99	LPG 22:6 sn-2	2.59E-04	1.73
LPI 16:0 sn-1	1.25E-03	1.78	Nervonic acid	1.03E-02	1.66	Methionine	2.66E-04	1.71
LPI 18:0 sn-1	1.05E-02	1.48	Octadecadienoic acid	1.51E-02	1.56	**Methylamine**	9.20E-04	1.76
LPI 22:6 sn-2	9.13E-03	1.52	**Palmitic acid**	1.12E-02	1.60	NAA (N-acetylaspartate)	1.30E-04	1.61
**Methylamine**	3.41E-03	1.60	Pantothenate	1.82E-02	1.57	Nicotinic acid	4.51E-02	1.64
MG 14:0 (1)	2.05E-05	2.15	Phosphorylethanolamine	1.82E-02	1.54	Oleic acid	1.80E-03	1.56
MG 18:1 (1)	3.58E-05	2.11	Pipecolic acid	8.84E-03	1.62	**Palmitic acid**	4.83E-05	1.88
Monoisoamylamine	2.67E-03	1.88	Pyroglutamic acid	2.84E-02	1.63	Phenylalanine	3.51E-04	1.72
N-Acetyl-L-glutamic acid	1.52E-02	1.49	Serine	4.48E-02	1.64	Pyridoxine	4.67E-04	1.59
Nicotinamide	6.63E-05	2.08	Taurine	1.72E-02	1.83	Pyroglutamic acid	9.82E-04	1.64
**Palmitic acid**	1.22E-03	1.83	**Threonine**	2.04E-02	1.94	Riboflavine	9.39E-05	1.99
Phosphocholine	4.65E-03	1.65	Trimethylamine-n-oxide	8.09E-04	2.26	Serine	8.24E-03	1.60

**Threonine**	2.31E-02	1.46	Tryptophan	2.83E-02	1.59	Taurine	8.59E-04	1.76
Trimethylamine-n-oxide	2.06E-03	1.85	Tyrosine	1.89E-02	1.78	Thiamine	1.12E-03	1.54
Uridine	1.77E-03	1.76	Xanthine	2.25E-02	1.74	Threonine	4.78E-03	1.76
			γ-Aminobutyric acid	2.02E-02	1.76	Tryptophan	5.47E-04	1.73
						Tyrosine	6.52E-04	1.81
						Uridine	4.17E-04	1.64
						Xanthine	7.09E-04	1.78
						γ-Aminobutyric acid	1.18E-03	1.72

**b.**	**1v5**			**2v5**			**3v5**	
**Compounds**	***p***	**VIP**	**Compounds**	***p***	**VIP**	**Compounds**	***p***	**VIP**
2-Hydroxybutyric acid	1.87E-03	1.57	2-Hydroxybutyric acid	7.54E-03	1.48	2-Hydroxyisovaleric acid	2.95E-03	1.92
3.4-Dihydroxyphenylacetic acid	5.11E-04	1.63	**5-Methylthioadenosine**	1.55E-05	1.93	**5-Methylthioadenosine**	8.31E-04	2.17
3-Hydroxybutyric acid	5.69E-06	1.87	**Arginine**	1.08E-05	1.96	**Arginine**	1.36E-05	2.41
5-Hydroxy indole-3-acetic acid	3.91E-04	1.63	**Aspartic acid**	7.73E-04	1.66	**Aspartic acid**	6.35E-04	2.04
**5-Methylthioadenosine**	3.31E-07	1.97	Azelaic acid	2.55E-03	1.52	Azelaic acid	5.54E-04	1.97
Arachidonic acid	5.48E-04	1.97	Betaine	2.79E-04	1.83	Citrulline	1.97E-02	1.52
**Arginine**	4.17E-05	1.76	Citrulline	2.43E-04	1.80	**Cytidine**	3.44E-04	2.10
**Aspartic acid**	2.18E-05	1.83	**Cytidine**	2.22E-03	1.51	**Cytosine**	5.05E-04	2.06
Carnitine	3.88E-05	1.78	**Cytosine**	3.60E-03	1.49	Docosahexaenoic acid	1.43E-02	1.62
**Cytidine**	2.58E-04	1.65	Dimethylamine	7.13E-04	1.68	Docosapentaenoic acid	5.01E-04	2.17
**Cytosine**	6.44E-04	1.60	Glutamic acid	8.86E-05	1.86	Eicosapentaenoic acid	1.95E-03	1.96
Dimethylamine	8.02E-05	1.75	Histidine	2.02E-04	1.78	Eicosatrienoic acid	2.87E-03	1.97
Guanosine	1.30E-03	1.51	**Inosine**	4.74E-05	1.88	Glutamic acid	3.21E-03	1.86
Histamine	4.32E-03	1.58	Lactic acid	2.41E-04	1.81	Glycerol	4.72E-03	1.81
**Inosine**	1.84E-07	1.99	LPG 18:1 sn-1	4.03E-02	1.61	Histidine	7.00E-03	1.75
Lactic acid	7.66E-05	1.77	LPG 22:6 sn-1	1.58E-04	1.80	**Inosine**	3.06E-04	2.22
LPG 22:6 sn-1	8.50E-04	1.51	LPG 22:6 sn-2	9.25E-04	1.70	LPC 20:5 sn-1	2.99E-02	1.45
**MG 14:0 (1)**	9.26E-06	1.82	Malic acid	9.00E-04	1.64	Lysine	2.00E-03	1.97
MG 18:1 (1)	2.02E-06	1.90	Methionine	1.22E-03	1.47	Malonic acid	2.46E-02	1.62
**NAA (N-acetylaspartate)**	1.49E-05	1.65	**MG 14:0 (1)**	1.14E-05	1.97	**MG 14:0 (1)**	1.04E-02	1.65
Palmitoylcarnitine	6.35E-04	1.61	MG 18:0 (2)	1.48E-03	1.63	**NAA (N-acetylaspartate)**	3.77E-06	2.31

Ribose	3.05E-04	1.65	MG 18:1 (1)	1.35E-04	1.86	Nicotinic acid	3.55E-02	2.13
Uracil	1.31E-04	1.58	**NAA (N-acetylaspartate**)	1.31E-05	1.81	N-oleoyl taurine	1.73E-02	1.69
**Uridine**	4.60E-06	1.90	N-Acetylneuraminic Acid	1.72E-03	1.60	Oleic acid	2.95E-02	1.50
			Nervonic acid	3.06E-03	1.55	Palmitic acid	6.31E-03	1.84
			Nicotinic acid	4.21E-02	1.59	Phosphocholine	4.00E-03	1.87
			Palmitoylcarnitine	6.15E-03	1.46	Phosphorylethanolamine	3.02E-04	2.08
			Phosphorylethanolamine	1.28E-03	1.59	Pipecolic acid	3.06E-02	1.52
			Pipecolic acid	5.64E-06	2.08	Proline	2.17E-02	1.57
			Proline	6.10E-04	1.73	Putrescine	1.40E-02	1.62
			Putrescine	3.82E-04	1.80	S-Adenosyl-L-homocysteine	9.46E-03	1.51
			Ribose	1.09E-03	1.62	Trimethyllysine N6,N6,N6	3.23E-02	1.51
			Rivoflavine	3.98E-03	1.59	Uracil	6.61E-03	1.74
			**Uridine**	1.41E-03	1.61	**Uridine**	2.51E-02	1.52
						Valine	1.24E-02	1.62

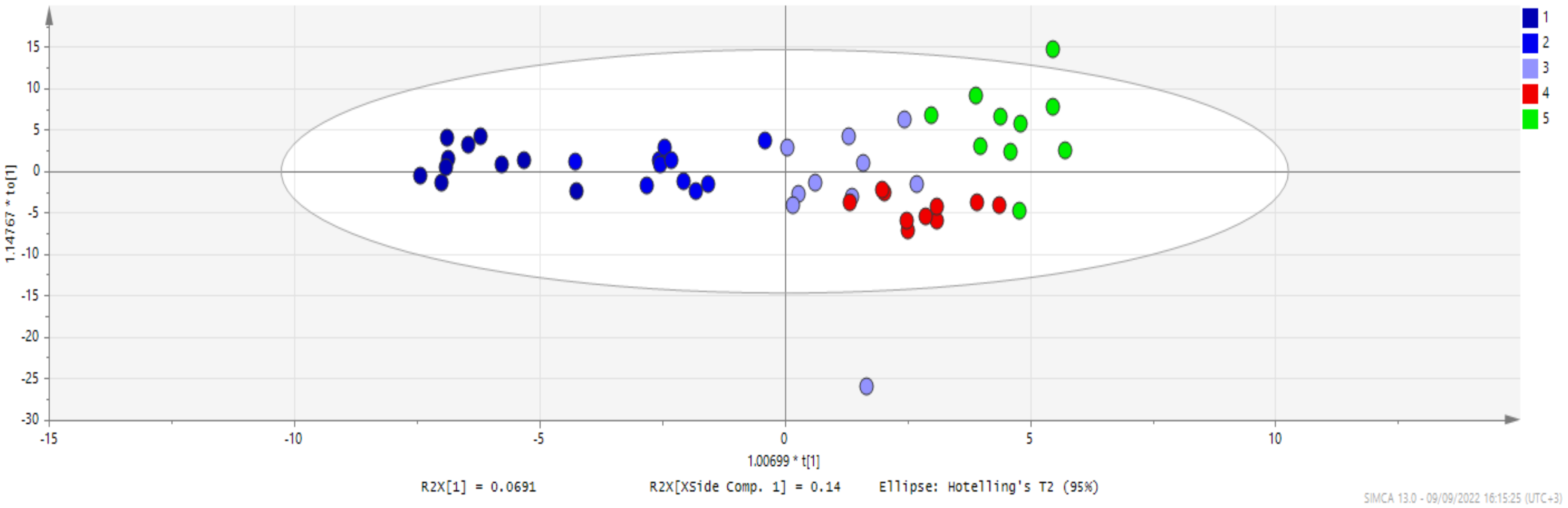

Figure S1. *OPLS score plot of the model constructed for the five studied groups. Infected mice (G4) are colored in red and controls (G5) are colored in green. The three treatment groups are shown in dark blue (G1), in blue (G2) and in light blue (G3).*

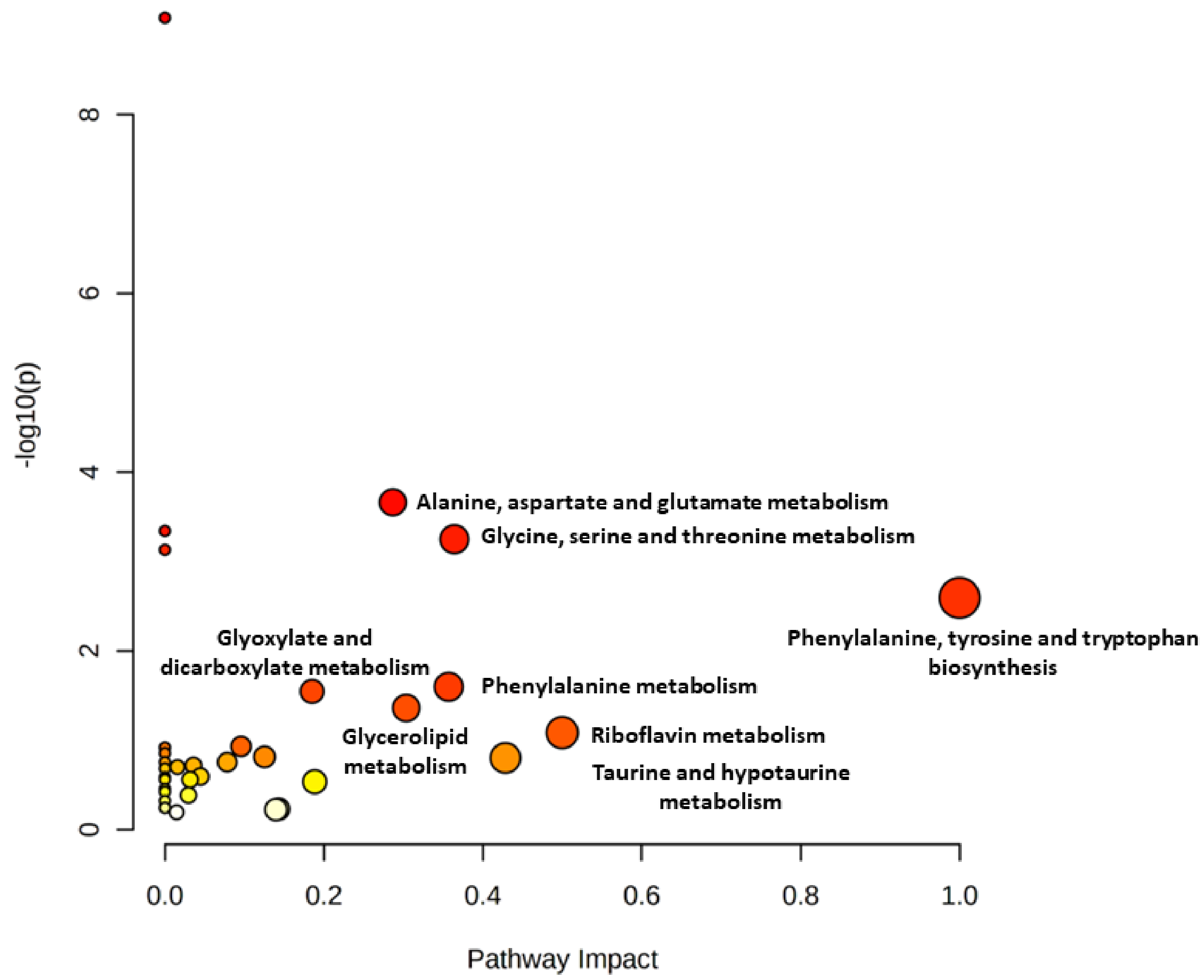

Figure S2*. Metabolic pathways highlighted by MetaboAnalyst 5.0 to have been affected by CDI based on the differentiated metabolites in brain. The x-axis represents the values of pathway impact extracted from the pathway topological analysis. The y-axis represents the -log of the p-value derived from the pathway enrichment analysis. The pathways with higher scores of both values were illustrated on top right region of the contracted plot.*
